# Integrative Generalized Convex Clustering Optimization and Feature Selection for Mixed Multi-View Data

**Published:** 2021-01

**Authors:** Minjie Wang, Genevera I. Allen

**Affiliations:** Department of Statistics, Rice University, Houston, TX 77005, USA; Departments of Electrical and Computer Engineering, Statistics, and Computer Science, Rice University, Houston, TX 77005, USA; Jan and Dan Duncan Neurological Research Institute, Baylor College of Medicine, Houston, TX 77030, USA

**Keywords:** Integrative clustering, convex clustering, feature selection, convex optimization, sparse clustering, GLM deviance, Bregman divergences

## Abstract

In mixed multi-view data, multiple sets of diverse features are measured on the same set of samples. By integrating all available data sources, we seek to discover common group structure among the samples that may be hidden in individualistic cluster analyses of a single data view. While several techniques for such integrative clustering have been explored, we propose and develop a convex formalization that enjoys strong empirical performance and inherits the mathematical properties of increasingly popular convex clustering methods. Specifically, our Integrative Generalized Convex Clustering Optimization (iGecco) method employs different convex distances, losses, or divergences for each of the different data views with a joint convex fusion penalty that leads to common groups. Additionally, integrating mixed multi-view data is often challenging when each data source is high-dimensional. To perform feature selection in such scenarios, we develop an adaptive shifted group-lasso penalty that selects features by shrinking them towards their loss-specific centers. Our so-called iGecco+ approach selects features from each data view that are best for determining the groups, often leading to improved integrative clustering. To solve our problem, we develop a new type of generalized multi-block ADMM algorithm using sub-problem approximations that more efficiently fits our model for big data sets. Through a series of numerical experiments and real data examples on text mining and genomics, we show that iGecco+ achieves superior empirical performance for high-dimensional mixed multi-view data.

## 1. Introduction

As the volume and complexity of data grows, statistical data integration has gained increasing attention as it can lead to discoveries which are not evident in analyses of a single data set. We study a specific data-integration problem where we seek to leverage common samples measured across multiple diverse sets of features that are of different types (e.g., continuous, count-valued, categorical, skewed continuous and etc.). This type of data is often called mixed, multi-view data (Hall and Llinas, 1997; Acar et al., 2011; Lock et al., 2013; Tang and Allen, 2018; Baker et al., 2019). While many techniques have been developed to analyze each individual data type separately, there are currently few methods that can directly analyze mixed multi-view data jointly. Yet, such data is common in many areas such as electronic health records, integrative genomics, multi-modal imaging, remote sensing, national security, online advertising, and environmental studies. For example in genomics, scientists often study gene regulation by exploring only gene expression data, but other data types, such as short RNA expression and DNA methylation, are all part of the same gene regulatory system. Joint analysis of such data can give scientists a more holistic view of the problem they study. But, this presents a major challenge as each individual data type is high-dimensional (i.e., a larger number of features than samples) with many uninformative features. Further, each data view can be of a different data type: expression of genes or short RNAs measured via sequencing is typically count-valued or zero-inflated plus skewed continuous data whereas DNA methylation data is typically proportion-valued. In this paper, we seek to leverage multiple sources of mixed data to better cluster the common samples as well as select relevant features that distinguish the inherent group structure.

We propose a convex formulation which integrates mixed types of data with different data-specific losses, clusters common samples with a joint fusion penalty and selects informative features that separate groups. Due to the convex formulation, our methods enjoy strong mathematical and empirical properties. We make several methodological contributions. First, we consider employing different types of losses for better handling non-Gaussian data with Generalized Convex Clustering Optimization (Gecco), which replaces Euclidean distances in convex clustering with more general convex losses. We show that for different losses, Gecco’s fusion penalty forms different types of centroids which we call loss-specific centers. To integrate mixed multi-view data and perform clustering, we incorporate different convex distances, losses, or divergences for each of the different data views with a joint convex fusion penalty that leads to common groups; this gives rise to Integrative Generalized Convex Clustering (iGecco). Further, when dealing with high-dimensional data, practitioners seek interpretability by identifying important features which can separate the groups. To facilitate feature selection in Gecco and iGecco, we develop an adaptive shifted group-lasso penalty that selects features by shrinking them towards their loss-specific centers, leading to Gecco+ and iGecco+ which performs clustering and variable selection simultaneously. To solve our problems in a computationally efficient manner, we develop a new general multi-block ADMM algorithm using sub-problem approximations, and make an optimization contribution by proving that this new class of algorithms converge to the global solution.

### 1.1 Related Literature

Our goal is to develop a unified, convex formulation of integrative clustering with feature selection based on increasingly popular convex clustering methods. Pelckmans et al. (2005); Lindsten et al. (2011); Hocking et al. (2011) proposed convex clustering which uses a fusion penalty to achieve agglomerative clustering like hierarchical clustering. This convex formulation guarantees a global optimal solution, enjoys strong statistical and mathematical theoretical properties, and often demonstrates superior empirical performance to competing approaches. Specifically, in literature, Pelckmans et al. (2005); Chi et al. (2017) showed it yields stable solutions to small perturbations on the data or tuning parameters; Radchenko and Mukherjee (2017) established clustering consistency by proving the clustering tree produced by convex clustering consistently estimates the clustering tree produced by the population procedure for the $\ell_{1}$ penalty case; Tan and Witten (2015) established its link to hierarchical clustering as well as prediction consistency (finite sample bound for the prediction error); the perfect recovery properties of convex clustering with uniform weights have been proved by Zhu et al. (2014) for the two-clusters case and Panahi et al. (2017) for the general *k*-clusters case while Sun et al. (2021) proved results for general weighted convex clustering model; and many others have studied other appealing theoretical properties (Wu et al., 2016; Chi and Steinerberger, 2019). Despite these advantages, convex clustering has not yet gained widespread popularity due to its intensive computation. Recently, some proposed fast and efficient algorithms to solve convex clustering and estimate its regularization paths (Chi and Lange, 2015; Weylandt et al., 2020). Meanwhile, convex clustering has been extended to biclustering (Chi et al., 2017) and many other applications (Chi et al., 2018; Choi et al., 2019).

One potential drawback to convex clustering however, is that thus far, it has only been well-studied employing Euclidean distances between data points and their corresponding cluster centers. As is well known, the Euclidean metric suffers from poor performance with data that is highly non-Gaussian such as binary, count-valued, skewed data, or with data that has outliers. To alleviate the impact of outliers, Wang et al. (2016) studied robust convex clustering, Sui et al. (2018) investigated convex clustering with metric learning and Wu et al. (2016) mentioned replacing $\ell_{2}$-norm with $\ell_{1}$-norm loss function as extensions. Despite these, however, no one has conducted a general investigation of convex clustering for non-Gaussian data, let alone studied data integration on mixed data, to the best of our knowledge. But, many others have proposed clustering methods for non-Gaussian data in other contexts. One approach is to perform standard clustering procedures on transformed data (Anders and Huber, 2010; Bullard et al., 2010; Marioni et al., 2008; Robinson and Oshlack, 2010). But, choosing an appropriate transformation that retains the original cluster signal is a challenging problem. Another popular approach is to use hierarchical clustering with specified distance metrics for non-Gaussian data (Choi et al., 2010; Fowlkes and Mallows, 1983). Closely related to this, Banerjee et al. (2005) studied different clustering algorithms utilizing a large class of loss functions via Bregman divergences. Yet, the proposed methods are all extensions of existing clustering approaches and hence inherit both good and bad properties of those approaches. There has also been work on model-based clustering, which assumes that data are generated by a finite mixture model; for example Banfield and Raftery (1993); Si et al. (2013) proposed such a model for the Poisson and negative binomial distributions. Still these methods have a non-convex formulation and local solutions like all model-based clustering methods. We propose to adopt the method similar to Banerjee et al. (2005) and study convex clustering using different loss functions; hence our method inherits the desirable properties of convex clustering and handles non-Gaussian data as well. More importantly, there is currently no literature on data integration using convex clustering and we achieve this by integrating different types of general convex losses with a joint fusion penalty.

Integrative clustering, however, has been well-studied in the literature. The most popular approach is to use latent variables to capture the inherent structure of multiple types of data. This achieves a joint dimension reduction and then clustering is performed on the joint latent variables (Shen et al., 2009, 2012, 2013; Mo et al., 2013, 2017; Meng et al., 2015). Similar in nature to the latent variables approach, matrix factorization methods assume that the data has an intrinsic low-dimensional representation, with the dimension often corresponding to the number of clusters (Lock et al., 2013; Hellton and Thoresen, 2016; Zhang et al., 2012; Chalise and Fridley, 2017; Zhang et al., 2011; Yang and Michailidis, 2015). There are a few major drawbacks of latent variable or dimension reduction approaches, however. First it is often hard to directly interpret latent factors or low-dimensional projections. Second, many of these approaches are based on non-convex formulations yielding local solutions. And third, choosing the rank of factors or projections is known to be very challenging in practice and will often impact resulting clustering solutions. Another approach to integrative clustering is clustering of clusters (COC) which performs cluster analysis on every single data set and then integrates the primary clustering results into final group assignments using consensus clustering (Hoadley et al., 2014; Lock and Dunson, 2013; Kirk et al., 2012; Savage et al., 2013; Wang et al., 2014). This, however, has several potential limitations as each individual data set might not have enough signal to discern joint clusters or the individual cluster assignments are too disparate to reach a meaningful consensus. Finally, others have proposed to use distance-based clustering for mixed types of data by first defining an appropriate distance metric for mixed data (for example, the Gower distance by Gower, 1971) and then applying an existing distance-based clustering algorithm such as hierarchical clustering (Ahmad and Dey, 2007; Ji et al., 2012). Consequently, this method inherits both good and bad properties of distance-based clustering approaches. Notice that all of these approaches are either two-step approaches or are algorithmic or non-convex problems that yield local solutions. In practice, such approaches often lead to unreliable and unstable results.

Clustering is known to perform poorly for high-dimensional data as most techniques are highly sensitive to uninformative features. One common approach is to reduce the dimensionality of the data via PCA, NMF, or t-SNE before clustering (Ghosh and Chinnaiyan, 2002; Bernardo et al., 2003; Tamayo et al., 2007). A major limitation of such approaches is that the resulting clusters are not directly interpretable in terms of feature importance. To address this, several have proposed sparse clustering for high-dimensional data. This performs clustering and feature selection simultaneously by iteratively applying clustering techniques to subsets of features selected via regularization (Witten and Tibshirani, 2010; Sun et al., 2012; Chang et al., 2014). The approach, however, is non-convex and is highly susceptible to poor local solutions. Others have proposed penalized model-based clustering that selects features (Raftery and Dean, 2006; Wang and Zhu, 2008; Pan and Shen, 2007). Still, these methods inherit several advantages and disadvantages of model-based clustering approaches. Moreover, sparse integrative clustering is relatively under-studied. Shen et al. (2013); Mo et al. (2013) extended iCluster using a penalized latent variable approach to jointly model multiple omics data types. They induced sparsity on the latent variable coefficients via regularization. As feature selection is performed on the latent variables, however, this is less interpretable in terms of selecting features directly responsible for distinguishing clusters. Recently, and most closely related to our own work, Wang et al. (2018) proposed sparse convex clustering which adds a group-lasso penalty term on the cluster centers to shrink them towards zero, thus selecting relevant features. This penalty, however, is only appropriate for Euclidean distances when the data is centered; otherwise, the penalty term shrinks towards the incorrect cluster centers. For feature selection using different distances and losses, we propose an adaptive shifted group-lasso penalty that will select features by shrinking them towards their appropriate centroid.

## 2. Integrative Generalized Convex Clustering with Feature Selection

In this section, we introduce our new methods, beginning with the Gecco and iGecco and then show how to achieve feature selection via regularization. We also discuss some practical considerations for applying our methods and develop an adaptive version of our approaches.

### 2.1 Generalized Convex Clustering Optimization (Gecco)

In many applications, we seek to cluster data that is non-Gaussian. In the literature, most do this using different distance metrics other than Euclidean distances (Choi et al., 2010; Fowlkes and Mallows, 1983; de Souza and De Carvalho, 2004). Some use losses based on exponential family or deviances closely related to Bregman divergences (Banerjee et al., 2005).

To account for different types of losses for non-Gaussian data, we propose to replace the Euclidean distances in convex clustering with more general convex losses; this gives rise to Generalized Convex Clustering Optimization (Gecco):

$$\underset{U}{\mathrm{minimize}}\sum_{i=1}^{n}\ell \left(X_{i.},U_{i.}\right)+\gamma \sum_{i<i^{\prime }}w_{ii^{\prime }}{\left\|U_{i.}-U_{i^{\prime }.}\right\|}_{q}.$$

Here, our data **X** is an *n* × *p* matrix consisting of *n* observations and *p* features; **U** is an *n* × *p* centroid matrix with the *i^th^* row, $U_{i.}$, the cluster centroid attached to point $X_{i.}$ The general loss $\ell \left(X_{i.},U_{i.}\right)$ refers to a general loss metric that measures dissimilarity between the data point $X_{i.}$ and assigned centroids $U_{i.}$. We choose one loss type as *ℓ* that is appropriate based on the data type of **X**. For example, we use *ℓ*_1_ loss in the presence of outliers. ${\left\|\cdot \right\|}_{q}$ is the *ℓ_q_*-norm of a vector and usually q∈{1,2,∞} is considered (Hocking et al., 2011). Here we prefer using the *ℓ*_2_-norm in the fusion penalty (*q* = 2) as it encourages the entire rows of similar observations to be fused together simultaneously and is also rotation-invariant; but one could use *ℓ*_1_ or *ℓ*_∞_-norm as well. *γ* is a positive tuning constant and *w_ij_* is a nonnegative weight. When *γ* equals zero, each data point occupies a unique cluster. As *γ* increases, the fusion penalty encourages some of the rows of the cluster center **U** to be exactly fused, forming clusters. When *γ* becomes sufficiently large, all centroids eventually coalesce to a single cluster centroid, which we define as the loss-specific center associated with ℓ(⋅). Hence *γ* regulates both the cluster assignment and number of clusters, providing a family of clustering solutions. The weight *w_ij_* should be specified by the user in advance and is not a tuning parameter; we discuss choices of weights for various convex losses in Section 2.5.

Going beyond Euclidean distances, we propose to employ convex distance metrics as well as deviances associated with exponential family distributions and Bregman divergences, which are always convex. Interestingly, we show that each of these possible loss functions shrinks the cluster centers, **U**, to different loss-specific centers, instead of the mean-based centroid as in convex clustering with Euclidean distances. For example, one may want to use least absolute deviations (*ℓ*_1_-norm or Manhattan distances) for skewed data or for data with outliers; with this loss, we show that all observations fuse to the median when *γ* is sufficiently large. We emphasize loss-specific centers here as they will be important in feature selection in the next section. For completeness, we list common distances and deviance-based losses, as well as their loss-specific centers ${\tilde{x}}_{j}$ respectively in Table 1. (See Appendix F for all calculations associated with loss-specific centers, and we provide a formal proof when studying the properties of our approaches in Section 2.4.)

### 2.2 Integrative Generalized Convex Clustering (iGecco)

In data integration problems, we observe data from multiple sources and would like to get a holistic understanding of the problem by analyzing all the data simultaneously. In our framework, we integrate mixed multi-view data and perform clustering by employing different convex losses for each of the different data views with a joint convex fusion penalty that leads to common groups. Hence we propose Integrative Generalized Convex Clustering (iGecco) which can be formulated as follows:

$$\underset{U^{\left(k\right)}}{\mathrm{minimize}}\sum_{k=1}^{K}\pi_{k}\ell_{k}\left(X^{\left(k\right)},U^{\left(k\right)}\right)+\gamma \sum_{i<i^{\prime }}w_{ii^{\prime }}\sqrt{\sum_{k=1}^{K}{\left\|U_{i.}^{\left(k\right)}-U_{i^{\prime }.}^{\left(k\right)}\right\|}_{2}^{2}}.$$

Here, we have *K* data sources. The *k^th^* data view **X**^(*k*)^ is an *n* × *p_k_* matrix consisting of *n* observations and *p_k_* features; **U**^(*k*)^ is also an *n* × *p_k_* matrix and the *i^th^* row, $U_{i.}^{\left(k\right)}$, is the cluster center associated with the point $X_{i.}^{\left(k\right)}$. And, $\ell_{k}\left(X_{i.}^{\left(k\right)},U_{i.}^{\left(k\right)}\right)$ is the loss function associated with the *k^th^* data view. Still, we choose one loss type as $\ell_{k}$ that is appropriate based on the data type of each view. Each loss function is weighted by $\pi_{k}$, which is fixed by the user in advance. We have found that setting $\pi_{k}$ to be inversely proportional to the null deviance evaluated at the loss-specific center, i.e., $\pi_{k}=\frac{1}{\ell_{k}\left(X^{\left(k\right)},{\tilde{X}}^{\left(k\right)}\right)}$, performs well in practice. The null deviance, $\ell_{k}\left(X^{\left(k\right)},{\tilde{X}}^{\left(k\right)}\right)$ refers to the loss function evaluated at ${\tilde{X}}^{\left(k\right)}$ where each *j^th^* column of ${\tilde{X}}^{\left(k\right)}$ denotes the loss-specific center ${\tilde{x}}_{j}^{\left(k\right)}$. We employ this loss function weighting scheme to ensure equal scaling across data sets of different types. Recall that in generalized linear model (GLM), the likelihood-ratio test statistic, or the difference between the log-likelihoods, $2\left(\ell_{k}\left(X^{\left(k\right)},{\hat{U}}^{\left(k\right)}\right)-\ell_{k}\left(X^{\left(k\right)},U_{0}^{\left(k\right)}\right)\right)$, follows a $\chi^{2}$-distribution. Here ${\hat{U}}^{\left(k\right)}$ is our iGecco estimate while $U_{0}^{\left(k\right)}$ is the loss-specific center ${\tilde{X}}^{\left(k\right)}$ (cluster centroid when there is only one cluster). Therefore, the ratio of the two quantities, i.e., $\frac{\ell_{k}\left(X^{\left(k\right)},U^{\left(k\right)}\right)}{\ell_{k}\left(X^{\left(k\right)},{\tilde{X}}^{\left(k\right)}\right)}=\pi_{k}\ell_{k}\left(X^{\left(k\right)},U^{\left(k\right)}\right)$ should be the same scale for each data view *k*. Finally, notice that we employ a joint convex fusion penalty on all of the **U**^(*k*)^’s; this incorporates information from each of the data sources and enforces the same group structure amongst the shared observations. Similar to convex clustering, our joint convex fusion penalty encourages the differences in the rows of concatenated centroids $\left(U^{\left(1\right)}\ldots U^{\left(K\right)}\right)$ to be shrunk towards zero, inducing a clustering behavior. Specifically, it forces the group structure of the *i^th^* row of **U**^(*k*)^ to be the same for all *k* data views. Note the joint convex fusion penalty can be also written as ${\left\|\left[\begin{array}{c} U_{i.}^{\left(1\right)}\\ \vdots \\ U_{i.}^{\left(K\right)} \end{array}\right]-\left[\begin{array}{c} U_{i^{\prime }.}^{\left(1\right)}\\ \vdots \\ U_{i^{\prime }.}^{\left(K\right)} \end{array}\right]\right\|}_{2}$. We say that subject *i* and *i′* belong to the same cluster if $U_{i.}^{\left(K\right)}=U_{i^{\prime }.}^{\left(K\right)}$, for all *k*. Hence, due to this joint convex fusion penalty, the common group structure property always holds. We study our methods further and prove some properties in Section 2.4.

### 2.3 Feature Selection: Gecco+ and iGecco+

In high dimensions, it is important to perform feature selection both for clustering purity and interpretability. Recently, Wang et al. (2018) proposed sparse convex clustering by imposing a group-lasso-type penalty on the cluster centers which achieves feature selection by shrinking noise features towards zero. This penalty, however, is only appropriate for Euclidean distances when the data is centered; otherwise, the penalty term shrinks towards the incorrect cluster centers. For example, the median is the cluster center with the *ℓ*_1_ or Manhattan distances. Thus, to select features in this scenario, we need to shrink them towards the median, and we should enforce “sparsity” with respect to the median and not the origin. To address this, we propose adding a shifted group-lasso-type penalty which forces cluster center **U**_·*j*_ to shrink towards the appropriate loss-specific center ${\tilde{x}}_{j}$ for each feature. Both the cluster fusion penalty and this new shifted-group-lasso-type feature selection penalty will shrink towards the same loss-specific center.

To facilitate feature selection with the adaptive shifted group-lasso penalty for one data type, our Generalized Convex Clustering Optimization with Feature Selection (Gecco+) is formulated as follows:

$$\underset{U}{\mathrm{minimize}}\sum_{i=1}^{n}\ell \left(X_{i.},U_{i.}\right)+\gamma \sum_{i<i^{\prime }}^{n}\omega_{ii^{\prime }}{\left\|U_{i.}-U_{i^{\prime }.}\right\|}_{2}+\alpha \sum_{j=1}^{p}\zeta_{j}{\left\|U_{.j}-{\tilde{x}}_{j}\cdot 1_{n}\right\|}_{2}.$$

Again, **U** is an *n* × *p* matrix and ${\tilde{x}}_{j}$ is the loss-specific center for the *j^th^* feature introduced in Table 1. The tuning parameter *α* controls the number of informative features and the feature weight $\zeta_{j}$ is a user input which plays an important role to adaptively penalize the features. (We discuss choices of $\zeta_{j}$ in Section 2.5.2 when we introduce the adaptive version of our method.) When *α* is small, all features are selected and contribute to defining the cluster centers. When *α* grows sufficiently large, all features coalesce at the same value, the loss-specific center ${\tilde{x}}_{j}$, and hence no features are selected and contribute towards determining the clusters. Another way of interpreting this is that the fusion penalty exactly fuses some of the rows of the cluster center **U**, hence determining groups of rows. On the other hand, the shifted group-lasso penalty shrinks whole columns of **U** towards their loss-specific centers, thereby essentially removing the effect of uninformative features. Selected features are then columns of **U** that are not shrunken to their loss-specific centers, $U_{.j}\neq {\tilde{x}}_{j}\cdot 1_{n}$. These selected features, then, exhibit differences across the clusters determined by the fusion penalty. Clearly, sparse convex clustering of Wang et al. (2018) is a special case of Gecco+ using Euclidean distances with centered data. Our approach using both a row and column penalty is also reminiscent of convex biclustering (Chi et al., 2017) which uses fusion penalties on both the rows and columns to achieve checker-board-like biclusters.

Building upon integrative generalized convex clustering in Section 2.2 and our proposed feature selection penalty above, our Integrative Generalized Convex Clustering Optimization with Feature Selection (iGecco+) is formulated as follows:

(1) $$\begin{array}{l} \underset{U^{\left(k\right)}}{\mathrm{minimize}}\sum_{k=1}^{K}\pi_{k}\ell_{k}\left(X^{\left(k\right)},U^{\left(k\right)}\right)+\gamma \sum_{i<i^{\prime }}w_{ii^{\prime }}\sqrt{\sum_{k=1}^{K}{\left\|U_{i.}^{\left(k\right)}-U_{i^{\prime }.}^{\left(k\right)}\right\|}_{2}^{2}}\\ \;\;\;\;+\alpha \sum_{k=1}^{K}\sum_{j=1}^{p_{k}}\zeta_{j}^{\left(k\right)}{\left\|U_{.j}^{\left(k\right)}-{\tilde{x}}_{j}^{\left(k\right)}\cdot 1_{n}\right\|}_{2}. \end{array}$$

Again, $U^{\left(k\right)}$ is an $n\times p_{k}$ matrix and ${\tilde{x}}_{j}^{\left(k\right)}$ is the loss-specific center for the *j^th^* feature for *k^th^* data view. By construction, iGecco+ directly clusters mixed multi-view data and selects features from each data view simultaneously. Similarly, adaptive choice of $\zeta_{j}^{\left(k\right)}$ gives rise to adaptive iGecco+ which will be discussed in Section 2.5.2. Detailed discussions on practical choices of tuning parameters and weights can be also found in Section 2.5.

### 2.4 Properties

In this section, we develop some properties of our methods, highlighting several advantages of our convex formulation. Corresponding proofs can be found in Section A of the Appendix.

Define the objective function in (1) as $F_{\gamma ,\alpha }\left(U\right)$ where $U=\left(U^{\left(1\right)}\ldots U^{\left(K\right)}\right)$. Then due to convexity, we have the following properties. First, any minimizer achieves a global solution.

**Proposition 1** *(Global solution) If ℓ_k_ is convex for all k, then any minimizer of* $F_{\gamma ,\alpha }\left(U\right)$, $U^{*}$, *is a global minimizer. If* $\ell_{k}$ *is strictly convex for all k, then* $U^{*}$ *is unique*.

Our method is continuous with respect to data, tuning parameters and input parameters.

**Proposition 2** *(Continuity with respect to data, tuning and input parameters) The global minimizer* $U_{w,\pi ,\zeta ,X}^{*}\left(\gamma ,\alpha \right)$ *of iGecco+ exists and depends continuously on the data*, **X**, *tuning parameters γ and α, the weight matrix w, the loss weight π_k_, and the feature weight* $\zeta_{j}^{\left(k\right)}$.

When tuning parameters are sufficiently large, all **U**’s coalesce to the loss-specific centers.

**Proposition 3** *(Loss-specific center) Define* $\tilde{X}=\left({\tilde{X}}^{\left(1\right)}\cdots {\tilde{X}}^{\left(K\right)}\right)$ *where each j^th^ column of* ${\tilde{X}}^{\left(k\right)}$ *equals the loss-specific center* ${\tilde{x}}_{j}^{\left(k\right)}$. *Suppose each observation corresponds to a node in a graph with an edge between nodes i and j whenever* $w_{ij}>0$. *If this graph is fully connected, then* $F_{\gamma ,\alpha }\left(U\right)$ *is minimized by the loss-specific center* $\tilde{X}$ *when γ is sufficiently large or α is sufficiently large*.

**Remark.** As Gecco, Gecco+ and iGecco are special cases of iGecco+, it is easy to show that all of our properties hold for these methods as well.

These properties illustrate some important advantages of our convex clustering approaches. Specifically, many other widely used clustering methods are known to suffer from poor local solutions, but any minimizer of our problem will achieve a global solution. Additionally, we show that iGecco+ is continuous with respect to the data, tuning parameters, and other input parameters. Together, these two properties are very important in practice and illustrate that the global solution of our method remains stable to small perturbations in the data and input parameters. Stability is a desirable property in practice as one would question the validity of a clustering result that can change dramatically with small changes to the data or parameters. Importantly, most popular clustering methods such as k-means, hierarchical clustering, model-based clustering, or low-rank based clustering, do not enjoy these same stability properties.

**Figure 1:**
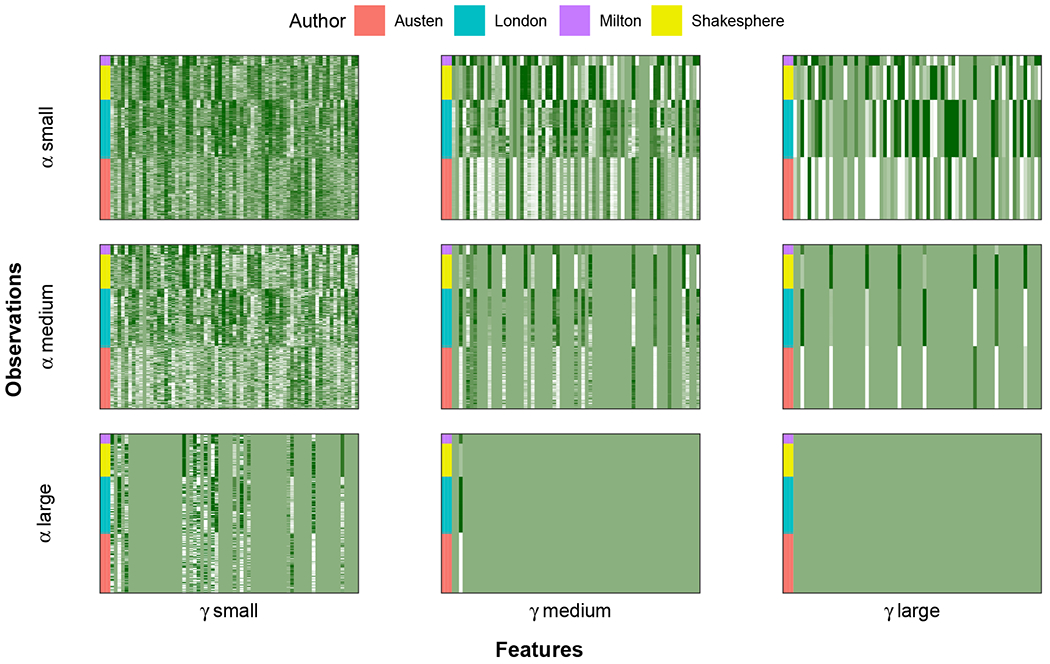
Regularization path of Gecco+ solutions $\hat{U}(\gamma ,\alpha )$ for authors data. From left to right, we increase the parameter for fusion penalty *γ*. From top to bottom, we increase the parameter for feature penalty *α*. The interpretation of regularization path is discussed in more detail in Section 2.4.

Finally in Proposition 3, we verify that when the tuning parameters are sufficiently large, full fusion of all observations to the loss-specific centers is achieved. Hence, our methods indeed behave as intended, achieving joint clustering of observations. We illustrate this property in Figure 1 where we apply Gecco+ to the authors data set (described fully in Section 4). Here, we illustrate how our solution, $\hat{U}\left(\gamma ,\alpha \right)$, changes as a function of *γ* and *α*. This so-called “cluster solution path” begins with each observation as its own cluster center when *γ* is small and stops when all observations are fused to the loss-specific center when *γ* is sufficiently large. In between, we see that observations are fusing together as *γ* increases. Similarly, when *α* is small, all features are selected and as *α* increases, some of the features get fused to their loss-specific center.

### 2.5 Practical Considerations and Adaptive iGecco+

In this section, we discuss some practical considerations for applying our method to real data. In the iGecco+ problem, $\pi_{k}$, *w* and $\zeta_{j}$ are user-specific fixed inputs while *γ* and *α* are tuning parameters; *γ* controls the number of clusters while *α* controls the number of features selected. We discuss choosing user-specific inputs such as weights as well as how to select tuning parameters. In doing so, we introduce an adaptive version of our method as well.

#### 2.5.1 Choice of Weights and Tuning Parameters

In practice, a good choice of fusion weights *w_ij_* has been shown to enhance both computational efficiency and clustering quality of convex clustering (Chi and Lange, 2015). It has been empirically demonstrated that using weights inversely proportional to the distances yields superior clustering performance; this approach is widely adopted in practice. Further, setting many of the weights to zero helps reduce computation cost. Considering these two, the most common weights choice for convex clustering is to use *K*-nearest-neighbors method with a Gaussian kernel. Specifically, the weight between the sample pair (*i, j*) is set as $w_{ij}=I_{ij}^{k}\exp\left(-\phi d\left(X_{i.},X_{j.}\right)\right)$, where $I_{ij}^{k}$ equals 1 if observation *j* is among observation *i*’s *κ* nearest neighbors or vice versa, and 0 otherwise. However, this choice of weights based on Euclidean distances may not work well for non-Gaussian data in Gecco(+) or for mixed data in iGecco(+). To account for different data types and better measure the similarity between observations, we still adopt *K*-nearest-neighbors method with an exponential kernel, but further extend this by employing appropriate distance metrics for specific data types in the exponential kernel. In particular, for weights in Gecco and Gecco+, we suggest using the same distance functions or deviances in the loss function of Gecco and Gecco+. For weights in iGecco and iGecco+, the challenge is that we need to employ a distance metric which measures mixed types of data. In this case, the Gower distance, which is a distance metric used to measure the dissimilarity of two observations measured in different data types (Gower, 1971), can address our problem. To be specific, the Gower distance between observation *i* and *i′* overall is defined as $d\left(X_{i.},X_{i^{\prime }.}\right)=\sum_{k=1}^{K}\sum_{j=1}^{p_{k}}d_{ii^{\prime }j}^{\left(k\right)}/\sum_{k=1}^{K}p_{k}$ where $d_{ii^{\prime }j}^{\left(k\right)}=\frac{\left|X_{ij}^{\left(k\right)}-X_{i^{\prime }j}^{\left(k\right)}\right|}{R_{j}^{\left(k\right)}}$ refers to the Gower distance between observation *i* and *i′* for feature *j* in data view *k* and $R_{j}^{\left(k\right)}=\max_{i,i^{\prime }}\left|X_{ij}^{\left(k\right)}-X_{i^{\prime }j}^{\left(k\right)}\right|$ is the range of feature *j* in data view *k*. In the literature, Gower distance has been commonly used as distance metrics for clustering mixed types of data (Wangchamhan et al., 2017; Hummel et al., 2017; Akay and Yüksel, 2018) and shown to yield superior performance than other distance metrics (Ali and Massmoudi, 2013; dos Santos and Zárate, 2015).

Alternatively, we also propose and explore using stochastic neighbor embedding weights based on symmetrized conditional probabilities (Maaten and Hinton, 2008). These have been shown to yield superior performance in high-dimensions and if there are potential outliers. Specifically, the symmetrized conditional probabilities are defined as $p_{ij}=\frac{p_{j|i}+p_{i|j}}{2n}$, where $p_{j|i}=\frac{\exp\left(-\phi d\left(X_{i.},X_{j.}\right)\right)}{\sum_{k\neq i}\exp\left(-\phi d\left(X_{i.},X_{k.}\right)\right)}.$ We propose to use the weights $w_{ij}=I_{ij}^{k}\cdot p_{ij}$ where $I_{ij}^{k}$ still equals 1 if observation *j* is among observation *i*’s *κ* nearest neighbors or vice versa, and 0 otherwise. Again, we suggest using distance metrics appropriate for specific data types or the Gower distance for mixed data. In empirical studies, we experimented with both weight choices and found that stochastic neighbor embedding weights tend to work better in high-dimensional settings and if there are outliers. Hence, we recommend these and employed them in our empirical investigations in Section 4 and 5.

Estimating the number of clusters in a data set is always challenging. Going beyond, we have two tuning parameters in our iGecco+ problem; *γ* controls the number of clusters while *α* controls the number of features selected. Current literature for tuning parameter selection for convex clustering mainly focuses on stability selection (Wang, 2010; Fang and Wang, 2012), hold-out validation (Chi et al., 2017) and information criterion (Tan and Witten, 2015). We first adopt the stability selection based approach for tuning parameter selection and follow closely the approach described in the work of Wang (2010); Fang and Wang (2012). We choose stability selection based approach because i) its selection consistency has been established and ii) Wang et al. (2018) adopted similar approach for tuning parameter selection and demonstrated strong empirical performance. However, stability selection is often computationally intensive in practice. To address this, we further explore information criterion based approaches like the Bayesian information criterion (BIC). We explain full details of both approaches in Appendix J and demonstrate empirical results when the number of clusters and features are not fixed but estimated based on the data.

#### 2.5.2 Adaptive Gecco+ and iGecco+ to Weight Features

Finally, we consider how to specify the feature weights, $\zeta_{j}$ used in the shifted group-lasso penalty. While employing these weights are not strictly necessary, we have found, as did Wang et al. (2018), that like the fusion weights, well-specified $\zeta_{j}’s$ can both improve performance and speed up computation. But unlike the fusion weights where we can use the pairwise distances, we don’t have prior information on which features may be relevant in clustering. Thus, we propose to use an adaptive scheme that first fits the iGecco+ with no feature weights and uses this initial estimate to define feature importance for use in weights. This is similar to many adaptive approaches in the literature (Zou, 2006; Wang et al., 2018).

Our adaptive iGecco+ approach is given in Algorithm 1; this applies to adaptive Gecco+ as a special case as well. We assume that the number of clusters (or a range of the number of clusters) is known a priori. We begin by fitting iGecco+ with *α* = 1 and uniform feature weights $\zeta_{j}^{\left(k\right)}=1$. We then find the *γ* which gives the desired number of clusters, yielding the initial estimate, ${\hat{U}}^{\left(k\right)}$. (We provide alternative adaptive iGecco+ Algorithm 17 when the number of clusters is not known in Appendix J.) Next, similar to the adaptive approaches by Zou (2006); Wang et al. (2018), we use this initial estimate to adaptively weight features by proposing the following weights: $\zeta_{j}^{\left(k\right)}=1/{\left\|{\hat{U}}_{.j}^{\left(k\right)}-{\tilde{x}}_{j}^{\left(k\right)}\cdot 1_{n}\right\|}_{2}$. (To avoid numerical issues, we add *ϵ* = 0.01 to the denominator.) These weights place a large penalty on noise features as ${\left\|{\hat{U}}_{.j}^{\left(k\right)}-{\tilde{x}}_{j}^{\left(k\right)}\cdot 1_{n}\right\|}_{2}$ is close to zero in this case. Note, compared with sparse convex clustering where the authors defined feature weights $\zeta_{j}$ by solving a convex clustering problem with feature penalty *α* = 0, we propose to fit iGecco+ with feature penalty *α* = 1 first and then update the feature weights adaptively. We find this weighting scheme works well in practice as it shrinks noise features more and hence penalizes more on those features. Such with-penalty initialization for adaptive weights has also been proposed in literature (Zhou et al., 2009; Fan et al., 2009; van de Geer et al., 2011). We also notice that noise features impact the distances used in the fusion weights as well. Hence, we suggest updating the distances adaptively by using the selected features to better measure the similarities between observations. To this end, we propose a new scheme to compute weighted Gower distances. First, we scale the features within each data view so that informative features in different data views contribute equally and on the same scale. Then, we employ the inverse of $\pi_{k}$, i.e., the null deviance, to weight the distances from different data types, resulting in an aggregated and weighted Gower distance, $\hat{d}\left(X_{i.},X_{i^{\prime }.}\right)$ as further detailed in Algorithm 1. Note that if the clustering signal from one particular data type is weak and there are few informative features for this data type, then our weighting scheme will down-weight this entire data type in the weighted Gower distance. In practice, our adaptive iGecco+ scheme works well as evidenced in our empirical investigations in the next sections.

**Algorithm 1:** Adaptive iGecco+

1. Fit iGecco+ with α=1, $\zeta_{j}^{\left(k\right)}=1$ and a sequence of *γ*.
2. Find *γ* which gives desired number of clusters; Get the estimate ${\hat{U}}^{\left(k\right)}$.
3. Update the feature weights ${\hat{\zeta }}_{j}^{\left(k\right)}=1/{\left\|{\hat{U}}_{.j}^{\left(k\right)}-{\tilde{x}}_{j}^{\left(k\right)}\cdot 1_{n}\right\|}_{2}$ and fusion weights ${\hat{w}}_{\mathrm{ij}}=I_{ij}^{\kappa }\exp\left(-\phi \hat{d}\left(X_{i.},X_{i^{\prime }.}\right)\right)$, where $\hat{d}\left(X_{i.},X_{i^{\prime }.}\right)=\sum_{k=1}^{K}\sum_{j=1}^{p_{k}}\frac{{\left\|{\hat{U}}_{.j}^{\left(k\right)}-{\tilde{x}}_{j}^{\left(k\right)}\cdot 1_{n}\right\|}_{2}}{\max_{j}{\left\|{\hat{U}}_{.j}^{\left(k\right)}-{\tilde{x}}_{j}^{\left(k\right)}\cdot 1_{n}\right\|}_{2}}\cdot \frac{1}{\pi_{k}}\cdot d_{ii^{\prime }j}^{\left(k\right)}.$
4. Fit adaptive iGecco+ with $\hat{\zeta }$, $\hat{\text{w}}$ and a sequence of *γ* and *α*; Find optimal *γ* and *α* which give desired number of clusters and features.

Note that Algorithm 1 for adaptive iGecco+ assumes desired number of clusters and features. (Panahi et al. (2017); Sun et al. (2021) proved that perfect recovery can be guaranteed for the general case of *q*-clusters for convex clustering. To yield exact *q* desired number of clusters, Weylandt et al. (2020) suggested back-tracking in practice.) We provide the alternative adaptive iGecco+ (Algorithm 17) in Appendix J when the number of clusters or features are not known a priori but estimated from the data.

## 3. iGecco+ Algorithm

In this section, we introduce our algorithm to solve iGecco+, which can be easily extended to Gecco, Gecco+ and iGecco. We first propose a simple, but rather slow ADMM algorithm as a baseline approach. To save computation cost, we further develop a new multi-block ADMM-type procedure using inexact one-step approximation of the sub-problems. Our algorithm is novel from optimization perspective as we extend the multi-block ADMM to a higher number of blocks and combine it with the literature related to inexact-solve ADMM with sub-problem approximations, which often results in major computational savings.

### 3.1 Full ADMM to Solve iGecco+ (Naive Algorithm)

Given the shifted group-lasso and fusion penalties along with general losses, developing an optimization routine for iGecco+ method is less straight-forward than convex clustering or sparse convex clustering. In this section, we propose a simple ADMM algorithm to solve iGecco+ as a baseline algorithm and point out its drawbacks.

The most common approach to solve problems with more than two non-smooth functions is via multi-block ADMM (Lin et al., 2015; Deng et al., 2017), which decomposes the original problem into several smaller sub-problems and solves them in parallel at each iteration. Chen et al. (2016) established a sufficient condition for the convergence of three-block ADMM. We develop a multi-block ADMM approach to fit our problem for certain types of losses and prove its convergence.

We first recast iGecco+ problem (1) as the equivalent constrained optimization problem:

$$\begin{array}{l} \underset{U^{\left(k\right)},V}{\mathrm{minimize}}\sum_{k=1}^{K}\pi_{k}\ell_{k}\left(X^{\left(k\right)},U^{\left(k\right)}\right)+\gamma \underset{P_{1}\left(V;w\right)}{\underbrace{\left(\sum_{l\in \varepsilon }w_{l}{\left\|V_{l.}\right\|}_{2}\right)}}+\alpha \sum_{k=1}^{K}\sum_{j=1}^{p_{k}}\zeta_{j}^{\left(k\right)}{\left\|U_{.j}^{\left(k\right)}-{\tilde{x}}_{j}^{\left(k\right)}\cdot 1_{n}\right\|}_{2}\\ \text{subject to}\;D\left[U^{\left(1\right)}\cdots U^{\left(K\right)}\right]-V=0. \end{array}$$

Recently, Weylandt et al. (2020) derived the ADMM for convex clustering in matrix form and we adopt similar approach. We index a centroid pair by $l=\left(l_{1},l_{2}\right)$ with $l_{1}<l_{2}$, define the set of edges over the non-zero weights $\varepsilon =\left\{l=\left(l_{1},l_{2}\right):w_{l}>0\right\}$, and introduce a new variable $V=\left[V^{\left(1\right)}\;\cdots \;V^{\left(K\right)}\right]\in R^{\left|\varepsilon \right|\times \Sigma p_{k}}$ where $V_{l.}^{\left(k\right)}=U_{l_{1.}}^{\left(k\right)}-U_{l_{2.}}^{\left(k\right)}$ to account for the difference between the two centroids. Hence $V^{\left(k\right)}$ is a matrix containing the pairwise differences between connected rows of $U^{\left(k\right)}$ and the constraint is equivalent to $DU^{\left(k\right)}-V^{\left(k\right)}=0$ for all *k*; $D\in R^{\left|\varepsilon \right|\times n}$ is the directed difference matrix corresponding to the non-zero fusion weights. We give general-form multi-block ADMM (Algorithm 2) to solve iGecco+. Here $prox_{h\left(\cdot \right)}\left(x\right)=\arg\min_{z}\frac{1}{2}{\left\|x-z\right\|}_{2}^{2}+h\left(z\right)$ is the proximal mapping of function *h*. Also, the superscript in $U^{\left(k\right)}$ in Algorithm 2 refers to the *k^th^* data view; we omit iteration counter indices in all iGecco+ algorithm for notation purposes and use the most recent values of the updates. The dual variable is denoted by $\Lambda^{\left(k\right)}$.

**Algorithm 2:** General multi-block algorithm for iGecco+

**while** not converged **do**
**for** all k=1,⋯,K **do**
$U^{\left(k\right)}=\underset{U}{\mathrm{argmin}}\;\pi_{k}\ell_{k}\left(X^{\left(k\right)},U\right)+\frac{\rho }{2}{\left\|DU-V^{\left(k\right)}+\Lambda^{\left(k\right)}\right\|}_{F}^{2}+\alpha \sum_{j=1}^{p_{k}}\zeta_{j}^{\left(k\right)}{\left\|U_{.j}-{\tilde{x}}_{j}^{\left(k\right)}\cdot 1_{n}\right\|}_{2}$
**end for**
$V={\text{prox}}_{\gamma /\rho P_{1}\left(\cdot ;\text{w}\right)}\left(\left[DU^{\left(1\right)}+\Lambda^{\left(1\right)}\cdots DU^{\left(K\right)}+\Lambda^{\left(K\right)}\right]\right)$
$\Lambda^{\left(k\right)}=\Lambda^{\left(k\right)}+\left(DU^{\left(k\right)}-V^{\left(k\right)}\right)\;\mathrm{for all}k$
**end while**

Notice that, in Algorithm 2, there is no closed-form analytical solution for the **U**^(*k*)^ sub-problem for general losses. Typically, at each iteration of Algorithm 2, we need to apply an inner optimization routine, which requires a nested iterative solver, to solve the **U**^(*k*)^ sub-problem until full convergence. In the next section, we seek to speed up this algorithm by using **U**^(*k*)^ sub-problem approximations. But, first we propose two different approaches to fully solve the **U**^(*k*)^ sub-problem based on specific loss types and then use these to develop a one-step update to solve the sub-problem approximately with guaranteed convergence. For the **V** sub-problem, one can easily show that it has a closed-form analytical solution for each iteration, as given in Algorithm 2.

### 3.2 iGecco+ Algorithm

We have introduced Algorithm 2, a simple baseline ADMM approach to solve iGecco+. In this section, we consider different ways to solve the **U**^(*k*)^ sub-problem in Algorithm 2. First, based on specific loss types (differentiable or non-differentiable), we propose two different algorithms to solve the **U**^(*k*)^ sub-problem to full convergence. These approaches, however, are rather slow for general losses as there is no closed-form solution which requires another nested iterative solver. To address this and in connection with current literature on variants of ADMM with sub-problem approximations, we propose iGecco+ algorithm, a multi-block ADMM algorithm which solves the sub-problems approximately by taking a single one-step update. We prove convergence of this general class of algorithms, a novel result in the optimization literature.

#### 3.2.1 Differentiable Case

When the loss *ℓ_k_* is differentiable, we consider solving the **U**^(*k*)^ sub-problem with proximal gradient descent, which is often used when the objective function can be decomposed into a differentiable and a non-differentiable function. While there are many other possible optimization routines to solve the **U**^(*k*)^ sub-problem, we choose proximal gradient descent as there is existing literature proving convergence of ADMM algorithms with approximately solved sub-problems using proximal gradient descent (Liu et al., 2013; Lu et al., 2016). We will discuss in detail how to approximately solve the sub-problem by taking a one-step approximation in Section 3.2.3. Based upon this, we propose Algorithm 3, which solves the **U**^(*k*)^ sub-problem by running full iterative proximal gradient descent to convergence. Here $P_{2}\left({\tilde{U}}^{\left(k\right)};\zeta^{\left(k\right)}\right)=\sum_{j=1}^{p_{k}}\zeta_{j}^{\left(k\right)}{\left\|{\tilde{U}}_{.j}^{\left(k\right)}\right\|}_{2}$.

**Algorithm 3:** **U**^(*k*)^ sub-problem for differentiable loss *ℓ_k_* (proximal gradient):

**while** not converged **do**
$U^{\left(k\right)}={\text{prox}}_{s_{k}\cdot \alpha P_{2}\left(\cdot ;\zeta^{\left(k\right)}\right)}\left(U^{\left(k\right)}-{\tilde{X}}^{\left(k\right)}-s_{k}\cdot \left[\pi_{k}\nabla \ell_{k}\left(X^{\left(k\right)},U^{\left(k\right)}\right)+\rho D^{T}\left(DU^{\left(k\right)}-V^{\left(k\right)}+\Lambda^{\left(k\right)}\right)\right]\right)+{\tilde{X}}^{\left(k\right)}$
**end while**

In Algorithm 3 and typically in general (proximal gradient) descent algorithms, we need to choose an appropriate step size *s_k_* to ensure convergence. Usually we employ a fixed step size by computing the Lipschitz constant as in the squared error loss case; but in our method, it is hard to compute the Lipschitz constant for most of our general losses. Instead, we suggest using backtracking line search procedure proposed by Beck and Teboulle (2009); Parikh et al. (2014), which is a common way to determine step size with guaranteed convergence in optimization. Further, we find decomposing the **U**^(*k*)^ sub-problem to *p_k_* separate $U_{.j}^{\left(k\right)}$ sub-problems brings several advantages such as i) better convergence property (than updating **U**^(*k*)^’s all together) due to adaptive step size for each $U_{.j}^{\left(k\right)}$ sub-problem and ii) less computation cost by solving each in parallel. Hence, in this case, we propose to use proximal gradient for each separate $U_{.j}^{\left(k\right)}$ sub-problem. To achieve this, we assume that the loss is elementwise, which is satisfied by every deviance-based loss. Last, as mentioned, there are many other possible ways to solve the **U**^(*k*)^ sub-problem than proximal gradient, such as ADMM. We find that when the loss function is squared Euclidean distances or the loss function has a Hessian matrix that can be upper bounded by a fixed matrix, using ADMM approach saves more computation. We provide all implementation details discussed above in Section C of the Appendix.

#### 3.2.2 Non-differentiable Case

When the loss *ℓ_k_* is non-differentiable, we can no longer adopt the proximal gradient method to solve the **U**^(*k*)^ sub-problem as the objective is now a sum of more than one separable non-smooth function. To address this, as mentioned, we can use multi-block ADMM; in this case, we introduce new blocks for the non-smooth functions and hence develop a full three-block ADMM approach to fit our problem.

To augment the non-differentiable term, we assume that our loss function can be written as $\ell_{k}\left(X^{\left(k\right)},U^{\left(k\right)}\right)=f_{k}\left(g_{k}\left(X^{\left(k\right)},U^{\left(k\right)}\right)\right)$ where *f_k_* is convex but non-differentiable and *g_k_* is affine. This condition is satisfied by all distance-based losses with $g_{k}\left(X^{\left(k\right)},U^{\left(k\right)}\right)=X^{\left(k\right)}-U^{\left(k\right)}$; for example, for Manhattan distances, we have $f_{k}\left(Z\right)=\sum_{j=1}^{p}{\left\|z_{j}\right\|}_{1}={\left\|\text{vec}\left(Z\right)\right\|}_{1}$, and $g_{k}\left(X,U\right)=X-U$. The benefit of doing this is that now the **U**^(*k*)^ sub-problem has a closed-form solution. Particularly, we can rewrite the **U**^(*k*)^ sub-problem as:

$$\begin{array}{l} \underset{U^{\left(k\right)},V}{\mathrm{minimize}}\sum_{k=1}^{K}\pi_{k}f_{k}\left(Z^{\left(k\right)}\right)+\frac{\rho }{2}{\left\|DU-V^{\left(k\right)}+\Lambda^{\left(k\right)}\right\|}_{F}^{2}+\alpha \sum_{k=1}^{K}\underset{P_{2}\left(R^{\left(k\right)};\zeta^{\left(k\right)}\right)}{\underbrace{\left(\sum_{j=1}^{p_{k}}\zeta_{j}^{\left(k\right)}{\left\|r_{j}^{\left(k\right)}\right\|}_{2}\right)}}\\ \text{subject to}\;X^{\left(k\right)}-U^{\left(k\right)}=Z^{\left(k\right)},\;U^{\left(k\right)}-{\tilde{X}}^{\left(k\right)}=R^{\left(k\right)}\;, \end{array}$$

where ${\tilde{X}}^{\left(k\right)}$ is an *n* × *p_k_* matrix with the *j^th^* column equal to scalar ${\tilde{x}}_{j}^{\left(k\right)}$.

It is clear that we can use multi-block ADMM to solve the problem above and each primal variable has a simple update with a closed-form solution. We propose Algorithm 4, a full, iterative multi-block ADMM, to solve the **U**^(*k*)^ sub-problem when the loss is a non-differentiable distance-based function. Algorithm 4 applies to iGecco+ with various distances such as Manhattan, Minkowski and Chebychev distances and details are given in Section D of the Appendix.

**Algorithm 4:** **U**^(*k*)^ sub-problem for non-differentiable distance-based loss *ℓ_k_* (multi-block ADMM):

**Precompute:** Difference matrix $D,M={\left(D^{T}D+2I\right)}^{-1}$.
**while** not converged **do**
$U^{\left(k\right)}=M\left(D^{T}\left(V^{\left(k\right)}-\Lambda^{\left(k\right)}\right)+{\tilde{X}}^{\left(k\right)}+R^{\left(k\right)}-N^{\left(k\right)}+X^{\left(k\right)}-Z^{\left(k\right)}+\Psi^{\left(k\right)}\right)$
$Z^{\left(k\right)}={\text{prox}}_{\pi_{k}f_{k}/\rho }\left(X^{\left(k\right)}-U^{\left(k\right)}+\Psi^{\left(k\right)}\right)$
$R^{\left(k\right)}={\text{prox}}_{\alpha /\rho P_{2}\left(\cdot ;\zeta^{\left(k\right)}\right)}\left(U^{\left(k\right)}-{\tilde{X}}^{\left(k\right)}+N^{\left(k\right)}\right)$
$\Psi^{\left(k\right)}=\Psi^{\left(k\right)}+\left(X^{\left(k\right)}-U^{\left(k\right)}-Z^{\left(k\right)}\right)$
$N^{\left(k\right)}=N^{\left(k\right)}+\left(U^{\left(k\right)}-{\tilde{X}}^{\left(k\right)}-R^{\left(k\right)}\right)$
**end while**

#### 3.2.3 iGecco+ Algorithm: Fast ADMM with Inexact One-step Approximation to the Sub-problem

Notice that for both Algorithm 3 and 4, we need to run them iteratively to full convergence in order to solve the **U**^(*k*)^ sub-problem in Algorithm 2 for each iteration, which is dramatically slow in practice. To address this, in literature, many have proposed variants of ADMM with guaranteed convergence that find an inexact, one-step, approximate solution to the sub-problem (without fully solving it); these include the generalized ADMM (Deng and Yin, 2016), proximal ADMM (Shefi and Teboulle, 2014; Banert et al., 2016) and proximal linearized ADMM (Liu et al., 2013; Lu et al., 2016). Thus, we propose to solve the **U**^(*k*)^ sub-problem approximately by taking a single one-step update of the algorithm (Algorithm 3 or 4) for both types of losses and prove convergence. For the differentiable loss case, we propose to apply the proximal linearized ADMM approach while for the non-differentiable case, we show that taking a one-step update of Algorithm 4, along with **V** and **Λ** update in Algorithm 2, is equivalent to applying a four-block ADMM to the original iGecco+ problem and we provide a sufficient condition for the convergence of four-block ADMM. Our algorithm, to the best of our knowledge, is the first to incorporate higher-order multi-block ADMM and inexact ADMM with a one-step update to solve sub-problems for general loss functions.

When the loss is differentiable, as mentioned in Algorithm 3, one can use full iterative proximal gradient to solve the $U_{.j}^{\left(k\right)}$ sub-problem, which however, is computationally burdensome. To avoid this, many proposed variants of ADMM which find approximate solutions to the sub-problems. Specifically, closely related to our problem here, Liu et al. (2013); Lu et al. (2016) proposed proximal linearized ADMM which solves the sub-problems efficiently by linearizing the differentiable part and then applying proximal gradient due to the non-differentiable part. We find their approach fits into our problem and hence develop a proximal linearized 2-block ADMM to solve iGecco+ when the loss *ℓ_k_* is differentiable and gradient is Lipschitz continuous. It can be shown that applying proximal linearized 2-block ADMM to Algorithm 2 is equivalent to taking a one-step update of Algorithm 3 along with **V** and **Λ** update in Algorithm 2. In this way, we avoid running full iterative proximal gradient updates to convergence for the **U**^(*k*)^ sub-problem as in Algorithm 3 and hence save computation cost.

When the loss is non-differentiable, we still seek to take an one-step update to solve the **U**^(*k*)^ sub-problem. We achieve this by noticing that taking a one-step update of Algorithm 4 along with **V** and **Λ** update in Algorithm 2 is equivalent to applying multi-block ADMM to the original iGecco+ problem recast as follows (for non-differentiable distance-based loss):

$$\begin{array}{l} \underset{U^{\left(k\right)},V}{\mathrm{minimize}}\;\sum_{k=1}^{K}\pi_{k}f_{k}\left(Z^{\left(k\right)}\right)+\gamma \underset{P_{1}\left(V;w\right)}{\underbrace{\left(\sum_{l\in \varepsilon }w_{l}{\left\|V_{l.}\right\|}_{2}\right)}}+\alpha \sum_{k=1}^{K}\underset{P_{2}\left(R^{\left(k\right)};\zeta^{\left(k\right)}\right)}{\left(\underbrace{\sum_{j=1}^{p_{k}}\zeta_{j}^{\left(k\right)}{\left\|r_{j}^{\left(k\right)}\right\|}_{2}}\right)}\\ \text{subject to}\;X^{\left(k\right)}-U^{\left(k\right)}=Z^{\left(k\right)},\;D\;\left[U^{\left(1\right)}\;\cdots \;U^{\left(K\right)}\right]-V=0,\;U^{\left(k\right)}-{\tilde{X}}^{\left(k\right)}=R^{\left(k\right)}. \end{array}$$

Typically, general higher-order multi-block ADMM algorithms do not always converge, even for convex functions (Chen et al., 2016). We prove convergence of our algorithm and establish a novel convergence result by casting the iGecco+ with non-differentiable losses as a four-block ADMM, proposing a sufficient condition for convergence of higher-order multi-block ADMMs, and finally showing that our problem satisfies this condition. (Details are given in the proof of Theorem 4 in Appendix B.) Therefore, taking a one-step update of Algorithm 4 converges for iGecco+ with non-differentiable losses.

So far, we have proposed inexact-solve one-step update approach for both differentiable loss and non-differentiable loss case. For mixed type of losses, we combine these two algorithms and this gives Algorithm 5, a multi-block ADMM algorithm with inexact one-step approximation to the **U**^(*k*)^ sub-problem to solve iGecco+. We also establish the following convergence result.

**Algorithm 5:** iGecco+ algorithm

**while** not converged **do**
**for** all k=1,⋯,K **do**
Update $U^{\left(k\right)}$:
**if** *ℓ_k_* is differentiable **then**
Take a one-step update of Algorithm 3
**else if** *ℓ_k_* is non-differentiable **then**
Take a one-step update of Algorithm 4
**end if**
**end for**
$V={\text{prox}}_{\gamma /\rho P_{1}\left(\cdot ;w\right)}\left(\left[DU^{\left(1\right)}+\Lambda^{\left(1\right)}\;\cdots \;DU^{\left(K\right)}+\Lambda^{\left(K\right)}\right]\right)$
$\Lambda^{\left(k\right)}=\Lambda^{\left(k\right)}+\left(DU^{\left(k\right)}-V^{\left(k\right)}\right)$ for all *k*
**end while**

**Theorem 4** *(iGecco+ convergence) Consider the iGecco+ problem* (1) *with fixed inputs π_k_, w and ζ_j_. If ℓ_k_ is convex for all k*, Algorithm 5 *converges to a global solution. In addition, if each ℓ_k_ is strictly convex, it converges to the unique global solution*.

**Remark.** Our corresponding Theorem 4 establishes a novel convergence result as it is the first to show the convergence of four-block or higher ADMM using approximate sub-problems for both differentiable and non-differentiable losses.

It is easy to see that Algorithm 5 can be applied to solve other Gecco-related methods as special cases. When *K* = 1, Algorithm 5 gives the algorithm to solve Gecco+. When *α* = 0, Algorithm 5 gives the algorithm to solve iGecco+. When *K* = 1 and *α* = 0, Algorithm 5 gives the algorithm to solve Gecco.

**Figure 2:**
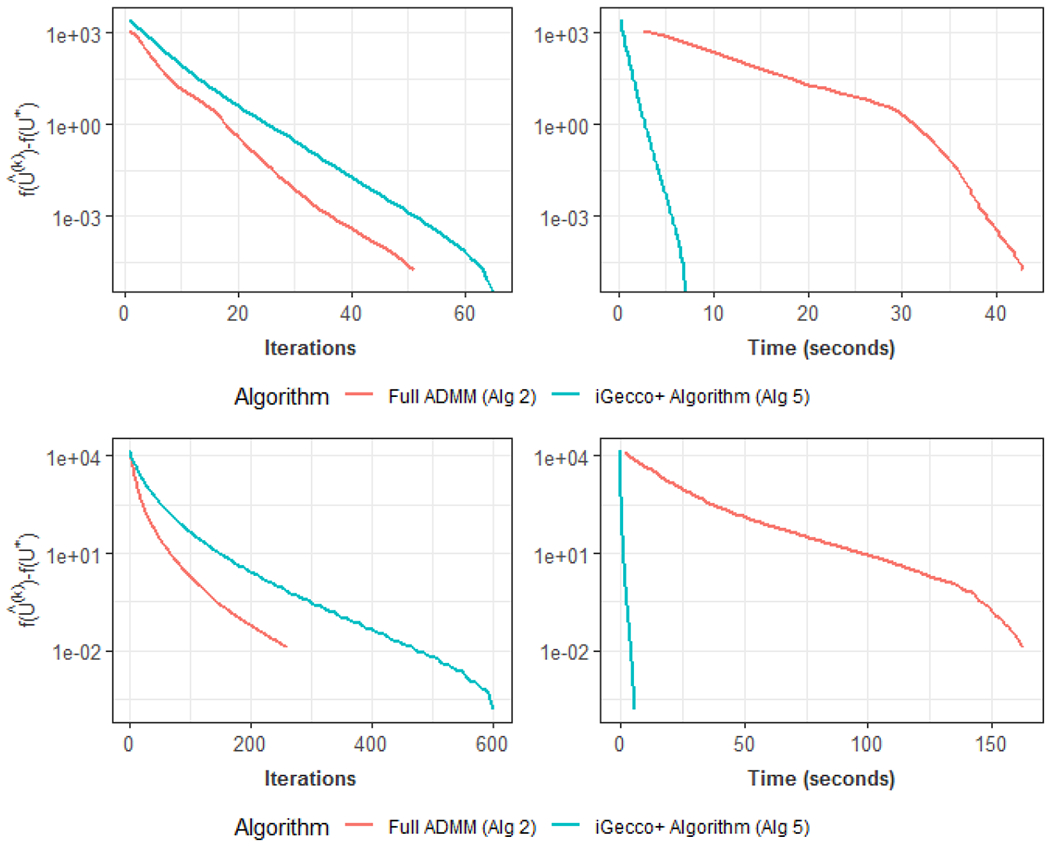
Comparisons of full ADMM and one-step ADMM to solve Gecco+ with Poisson log-likelihood (top panel, differentiable loss) and Gecco+ with Manhattan distances (bottom panel, non-differentiable loss). Left plots show the number of iterations needed to converge while right plots show computation time. Algorithm with one-step update to solve the sub-problem saves much more computational time.

To conclude this section, we compare the convergence results of using both full ADMM and inexact ADMM with one-step update in the sub-problem to solve Gecco+ (*n* = 120 and *p* = 210) in Figure 2. The left plots show the number of iterations needed to yield optimization convergence while the right plots show computation time. We see that Algorithm 5 (one-step update to solve the sub-problem) saves much more computational time than Algorithm 2 (full updates to solve the sub-problem). It should be pointed out that though Algorithm 5 takes more iterations to converge due to inexact approximation for each iteration, we still reduce computation time dramatically as the computation time per iteration is much less than the full-solve approach.

## 4. Simulation Studies

In this section, we first evaluate the performance of Gecco+ against existing methods on non-Gaussian data. Next we compare iGecco+ with other methods on mixed multi-view data.

### 4.1 Non-Gaussian Data

In this subsection, we evaluate the performance of Gecco and (adaptive) Gecco+ by comparing it with k-means, hierarchical clustering and sparse convex clustering. For simplicity, we have the following naming convention for all methods: loss type name + Gecco(+). For example, Poisson Deviance Gecco+ refers to Generalized Convex Clustering with Feature Selection using Poisson deviance. Sparse CC refers to sparse convex clustering using Euclidean distances where each feature is centered first. We measure the accuracy of clustering results using adjusted Rand index (Hubert and Arabie, 1985). The adjusted Rand index is the corrected-for-chance version of the Rand index, which is used to measure the agreement between the estimated clustering assignment and the true group label. A larger adjusted Rand index implies a better clustering result. For all methods we consider, we assume oracle number of clusters for fair comparisons.

Each simulated data set is comprised of *n* = 120 observations with 3 clusters. Each cluster has an equal number of observations. Only the first 10 features are informative while the rest are noise. We consider the following simulation scenarios.

- S1: Spherical data with outliers
  The first 10 informative features in each group are generated from a Gaussian distribution with different *μ_k_*’s for each class. Specifically, the first 10 features are generated from *N*(*μ_k_*, **I**_10_) where $\mu_{1}={\left(-2.5\cdot 1_{5}^{T},0_{5}^{T}\right)}^{T}$, $\mu_{2}={\left(0_{5}^{T},2.5\cdot 1_{5}^{T}\right)}^{T}$, $\mu_{3}={\left(2.5\cdot 1_{5}^{T},0_{5}^{T}\right)}^{T}$. The outliers in each class are generated from a Gaussian distribution with the same mean centroid *μ_k_* but with higher variance, i.e., *N*(*μ_k_*, 25 · **I**_10_). The remaining noise features are generated from *N*(0,1).
  In the first setting (S1A), number of noise features ranges in 25, 50, 75, ⋯ up to 225 with the proportion of number of outliers fixed ( = 5%). We also consider the setting when the variance of noise features increases with number of features fixed *p* = 200 and number of outliers fixed (S1B) and high-dimensional setting where *p* ranges from 250, 500, 750 to 1000 (S1C).
- S2: Non-spherical data with three half moons
  Here we consider the standard simulated data of three interlocking half moons as suggested by Chi and Lange (2015) and Wang et al. (2018). The first 10 features are informative in which each pair makes up two-dimensional three interlocking half moons. We randomly select 5% of the observations in each group and make them outliers. The remaining noise features are generated from *N*(0,1). The number of noise features ranges from 25, 50, 75, ⋯ up to 225. In both S1 and S2, we compare Manhattan Gecco+ with other existing methods.
- S3: Count-valued data
  The first 10 informative features in each group are generated from a Poisson distribution with different *μ_k_*’s (*i* = 1, 2, 3) for each class. Specifically, *μ*_1_ = 1 · **1**_10_, *μ*_2_ = 4 · **1**_10_, *μ*_3_ = 7 · **1**_10_. The remaining noise features are generated from a Poisson distribution with the same *μ*’s which are randomly generated integers from 1 to 10. The number of noise features ranges from 25, 50, 75, ⋯ up to 225.

**Figure 3:**
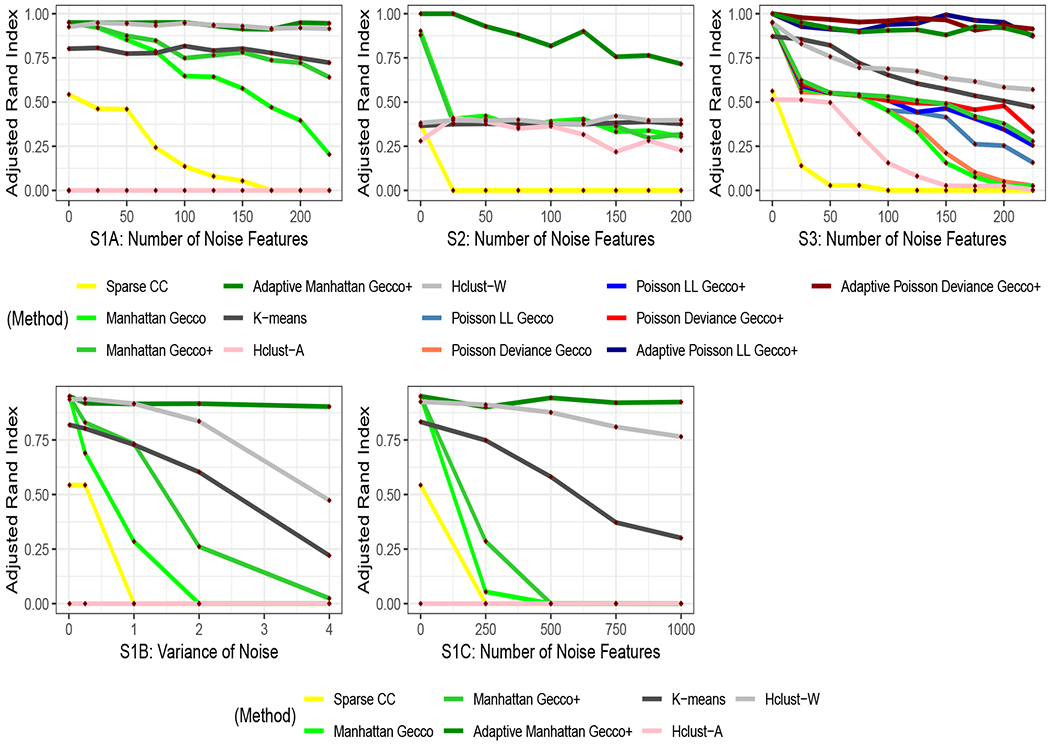
Simulation results of non-Gaussian data: (S1A) We increase number of noise features for spherical data with outliers; (S2) We increase number of noise features for non-spherical data with outliers; (S3) We increase number of noise features for count-valued data; (S1B) We increase noise level for spherical data with outliers; (S1C) We further increase number of noise features for spherical data with outliers in high dimensions. The adaptive Gecco+ outperforms existing methods in high dimensions.

We summarize simulation results in Figure 3. We find that for spherical data with outliers, adaptive Manhattan Gecco+ performs the best in high dimensions. Manhattan Gecco performs well in low dimensions but poorly as the number of noisy features increases. Manhattan Gecco+ performs well as the dimension increases, but adaptive Manhattan Gecco+ outperforms the former as it adaptively penalizes the features, meaning that noisy features quickly get zeroed out in the clustering path and that only the informative features perform important roles in clustering. We see that, without adaptive methods, we do not achieve the full benefit of performing feature selection. As we perform adaptive Gecco+, we show vast improvement in clustering purity as the number of noise features grows where regular Gecco performs poorly. Sparse convex clustering performs the worst as it tends to pick outliers as singleton clusters. In the presence of outliers, Manhattan Gecco+ performs much better than sparse convex clustering as we choose a loss function that is more robust to outliers. Interestingly, k-means performs better than sparse convex clustering. This is mainly because sparse convex clustering calculates pairwise distance in the weights, placing outliers in singleton clusters more likely than k-means which calculates within-cluster variances (where outliers could be closer to the cluster centroids than to other data points). Our simulation results also show that adaptive Manhattan Gecco+ works well for non-spherical data by selecting the correct features. For count data, all three adaptive Gecco+ methods perform better than k-means, hierarchical clustering and sparse convex clustering. We should point out that there are several linkage options for hierarchical clustering. For visualization purposes, we only show the linkage with the best and worst performance instead of all the linkages. Also we use the appropriate data-specific distance metrics in hierarchical clustering. For k-means, we use k-means++ (Arthur and Vassilvitskii, 2006) for initialization.

Table 2 shows the variable selection accuracy of sparse convex clustering and adaptive Gecco+ in terms of F_1_ score. In all scenarios, we fix *p* = 225. We see that adaptive Gecco+ selects the correct features, whereas sparse convex clustering performs poorly.

### 4.2 Multi-View Data

In this subsection, we evaluate the performance of iGecco and (adaptive) iGecco+ on mixed multi-view data by comparing it with hierarchical clustering, iClusterPlus (Mo et al., 2013) and Bayesian Consensus Clustering (Lock and Dunson, 2013). Again, we measure the accuracy of clustering results using the adjusted Rand index (Hubert and Arabie, 1985).

As before, each simulated data set is comprised of *n* = 120 observations with 3 clusters. Each cluster has an equal number of observations. Only the first 10 features are informative while the rest are noise. We have three data views consisting of continuous data, count-valued data and binary/proportion-valued data. We investigate different scenarios with different dimensions for each data view and consider the following simulation scenarios:

- S1: Spherical data with *p*_1_ = *p*_2_ = *p*_3_ = 10
- S2: Three half-moon data with *p*_1_ = *p*_2_ = *p*_3_ = 10
- S3: Spherical data with *p*_1_ = 200, *p*_2_ = 100, *p*_3_ = 50
- S4: Spherical data with *p*_1_ = 50, *p*_2_ = 200, *p*_3_ = 100
- S5: Three half-moon data with *p*_1_ = 200, *p*_2_ = 100, *p*_3_ = 50
- S6: Three half-moon data with *p*_1_ = 50, *p*_2_ = 200, *p*_3_ = 100

We employ a similar simulation setup as in Section 4.1 to generate each data view. The difference is that here for informative features, we increase the within-cluster variance for Gaussian data and decrease difference of cluster mean centroids *μ_k_*’s for binary and count data so that there is overlap between different clusters. Specifically, for spherical cases, Gaussian data is generated from *N*(*μ_k_*, 3 · **I**_10_); count data is generated from Poisson with different *μ_k_*’s (*μ*_1_ = 2, *μ*_2_ = 4, *μ*_3_ = 6, etc); binary data is generated from Bernoulli with different *μ_k_*’s (*μ*_1_ = 0.5, *μ*_2_ = 0.2, *μ*_3_ = 0.8, etc). For half-moon cases, continuous data is simulated with larger noise and the count and proportion-valued data is generated via a copula transform. In this manner, we have created a challenging simulation scenario where accurate clustering results cannot be achieved by considering only a single data view.

Again, for fair comparisons across methods, we assume oracle number of clusters. When applying iGecco(+) methods, we employ Euclidean distances for continuous data, Manhattan distances for count-valued data and Bernoulli log-likelihood for binary or proportion-valued data. We use the latter two losses as they perform well compared with counterpart losses in Gecco+ and demonstrate faster computation speed.

Simulation results in Table 3 and Table 4 show that our methods perform better than existing methods. In low dimensions, iGecco performs comparably with iCluster and Bayesian Consensus Clustering for spherical data. For non-spherical data, iGecco performs much better. For high dimensions, iGecco+ performs better than iGecco while adaptive iGecco+ performs the best as it achieves the full benefit of feature selection. We also applied k-means to the simulated data. The results of k-means are close to (or in some cases worse than) hierarchical clustering with the best-performing linkage; hence we only show the results of hierarchical clustering here for comparison.

Also we show the variable selection results in Table 5 and compare our method to that of iClusterPlus. For fair comparisons, we assume oracle number of features. For our method, we choose *α* that gives oracle number of features; for iClusterPlus, we select top features based on Lasso coefficient estimates. Our adaptive iGecco+ outperforms iClusterPlus for all scenarios.

Note in this section, we assume that the oracle number of clusters and features are known a priori for fair comparisons. Results when the number of clusters and features are not fixed but estimated based on the data using tuning parameter selection, are given in Appendix J.3.

## 5. Real Data Examples

In this section, we apply our methods to various real data sets and evaluate our methods against existing ones. We first evaluate the performance of Gecco+ for several real data sets and investigate the features selected by various Gecco+ methods.

### 5.1 Authors Data

The authors data set consists of word counts from *n* = 841 chapters written by four famous English-language authors (Austen, London, Shakespeare, and Milton). Each class contains an unbalanced number of observations with 69 features. The features are common “stop words” like “a”, “be” and “the” which are typically removed before text mining analysis. We use Gecco+ not only to cluster book chapters and compare the clustering assignments with true labels of authors, but also to identify which key words help distinguish the authors. We choose tuning parameters using BIC based approach; results for stability selection based approach are given in Table 15, Appendix J.3.

In Table 6, we compare Gecco+ with existing methods in terms of clustering quality. For hierarchical clustering, we only show the linkage with the best performance (in this whole section). Our method outperforms k-means and the best hierarchical clustering method. Secondly, we look at the word texts selected by Gecco+. As shown in Table 7, Jane Austen tended to use the word “her” more frequently than the other authors; this is expected as the subjects of her novels are typically females. The word “was” seems to separate Shakespeare and Jack London well. Shakespeare preferred to use present tense more while Jack London preferred to use past tense more. To summarize, our Gecco+ not only has superior clustering performance but also selects interpretable features.

### 5.2 TCGA Breast Cancer Data

The TCGA data set consists of log-transformed Level III RPKM gene expression levels for 445 breast-cancer patients with 353 features from The Cancer Genome Atlas Network (The Cancer Genome Atlas Network, 2012). Five PAM50 breast cancer subtypes are included, i.e., Basal-like, Luminal A, Luminal B, HER2-enriched, and Normal-like. Only 353 genes out of 50,000 with somatic mutations from COSMIC (Forbes et al., 2010) are retained. The data is Level III TCGA BRCA RNA-Sequencing gene expression data that have already been pre-processed according to the following steps: i) reads normalized by RPKM, and ii) corrected for overdispersion by a log-transformation. We remove 7 patients, who belong to the normal-like group and the number of subjects *n* becomes 438. We also combine Luminal A with Luminal B as they are often considered one aggregate group (Choi et al., 2014).

From Table 8, our method outperforms k-means and the best hierarchical clustering method. Next, we look at the genes selected by Gecco+ in Table 9. FOXA1 is known to be a key gene that characterizes luminal subtypes in DNA microarray analyses (Badve et al., 2007). GATA binding protein 3 (GATA3) is a transcriptional activator highly expressed by the luminal epithelial cells in the breast (Mehra et al., 2005). ERBB2 is known to be associated with HER2 subtype and has been well studied in breast cancer (Harari and Yarden, 2000). Hence our Gecco+ not only outperforms existing methods but also selects genes which are relevant to biology and have been implicated in previous scientific studies.

Next we evaluate the performance of iGecco+ for mixed multi-view data sets and investigate the features selected by iGecco+.

### 5.3 Multi-omics Data

One promising application for integrative clustering for multi-view data lies in integrative cancer genomics. Biologists seek to integrate data from multiple platforms of high-throughput genomic data to gain a more thorough understanding of disease mechanisms and detect cancer subtypes. In this case study, we seek to integrate four different types of genomic data to study how epigenetics and short RNAs influence the gene regulatory system in breast cancer.

We use the data set from The Cancer Genome Atlas Network (2012). Lock and Dunson (2013) analyzed this data set using integrative methods and we follow the same data pre-processing procedure: i) filter out genes in expression data whose standard deviation is less than 1.5, ii) take square root of methylation data, and iii) take log of miRNA data. We end up with a data set of 348 tumor samples including:

- RNAseq gene expression (GE) data for 645 genes,
- DNA methylation (ME) data for 574 probes,
- miRNA expression (miRNA) data for 423 miRNAs,
- Reverse phase protein array (RPPA) data for 171 proteins.

The data set contains samples used on each platform with associated subtype calls from each technology platform as well as integrated cluster labels from biologists. We use the integrated labels from biologists as true label to compare different methods. Also we merged the subtypes 3 and 4 in the integrated labels as those two subtypes correspond to Luminal A and Luminal B respectively from the predicted label given by gene expression data (PAM50 mRNA).

Figure 6 in Appendix H gives the distribution of data from different platforms. For our iGecco+ methods, we use Euclidean distances for gene expression data and protein data as the distributions of these two data sets appear Gaussian; binomial deviances for methylation data as the value is between [0, 1]; Manhattan distances for miRNA data as the data is highly-skewed.

We compare our adaptive iGecco+ with other existing methods. From Table 10, we see that our method outperforms all the existing methods.

We also investigate the features selected by adaptive iGecco+, shown in Table 11, and find that our method is validated as most are known in the breast cancer literature. For example, FOXA1 is known to segregate the luminal subtypes from the others (Badve et al., 2007), and AGR3 is a known biomarker for breast cancer prognosis and early breast cancer detection from blood (Garczyk et al., 2015). Several well-known miRNAs were selected including MIR-135b, which is upregulated in breast cancer and promotes cell growth (Hua et al., 2016) as well as MIR-190 which suppresses breast cancer metastasis (Yu et al., 2018). Several known proteins were also selected including ERalpha, which is overexpressed in early stages of breast cancer (Hayashi et al., 2003) and GATA3 which plays an integral role in breast luminal cell differentiation and breast cancer progression (Cimino-Mathews et al., 2013).

**Figure 4:**
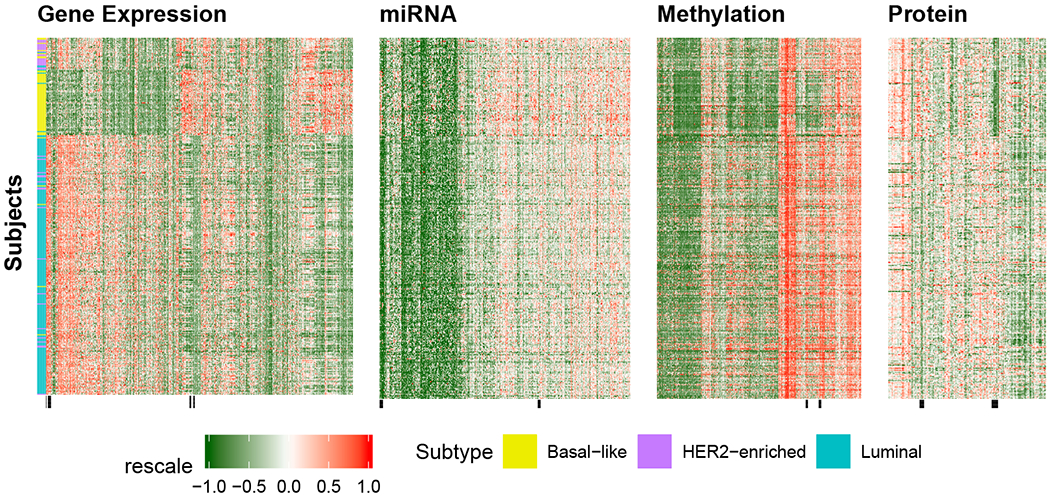
Cluster heatmap of multi-omics TCGA data with row orders determined by cluster assignments from iGecco+. The left bar refers to the integrated cluster labels from biologists. The black bars at the bottom of each data view correspond to the selected features. Our adaptive iGecco+ identifies meaningful subtypes.

**Figure 6:**
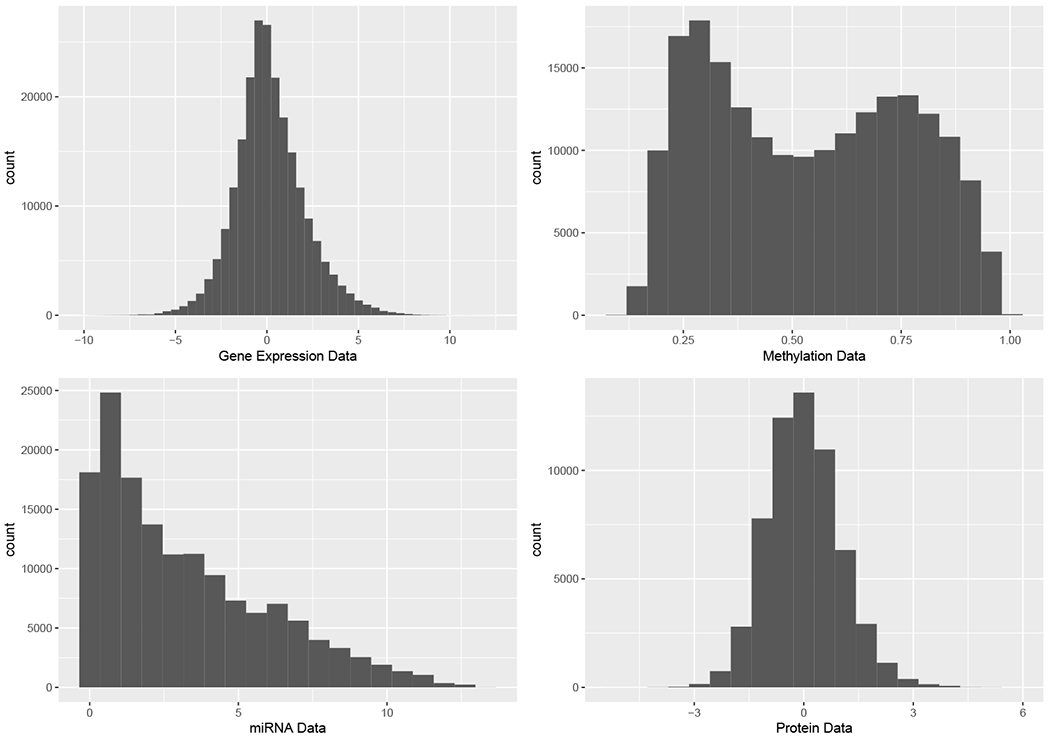
Histograms of data from different platforms for multi-omics TCGA data set. Both gene expression data and protein data appear Gaussian; methylation data is proportion-valued; miRNA data is highly-skewed.

We also visualize the resulting clusters from adaptive iGecco+. Figure 4 shows the cluster heatmap of multi-omics TCGA data with row orders determined by cluster assignments from iGecco+ and left bar corresponding to the integrated cluster labels from biologists. We see that there is a clear separation between groups and adaptive iGecco+ identifies meaningful subtypes. The black bars at the bottom of each data view correspond to the selected features in Table 11.

## 6. Discussion

In this paper, we develop a convex formulation of integrative clustering for high-dimensional mixed multi-view data. We propose a unified, elegant methodological solution to two critical issues for clustering and data integration: i) dealing with mixed types of data and ii) selecting sparse, interpretable features in high-dimensional settings. Specifically, we show that clustering for mixed, multi-view data can be achieved using different data-specific convex losses with a joint fusion penalty. We also introduce a shifted group-lasso penalty that shrinks noise features to their loss-specific centers, hence selecting features that play important roles in separating groups. In addition, we make an optimization contribution by proposing and proving the convergence of a new general multi-block ADMM algorithm with sub-problem approximations that efficiently solves our problem. Empirical studies show that iGecco+ outperforms existing clustering methods and selects sparse, interpretable features in separating clusters.

This paper focuses on the methodological development for integrative clustering and feature selection, but there are many possible avenues for future research related to this work. For example, we expect in future work to be able to show that our methods inherit the strong statistical and theoretical properties of other convex clustering approaches such as clustering consistency and prediction consistency. An important problem in practice is choosing which loss function is appropriate for a given data set. While this is beyond the scope of this paper, an interesting direction for future research would be to learn the appropriate convex loss function in a data-driven manner. Additionally, many have shown block missing structure is common in mixed data (Yu et al., 2019; Xiang et al., 2013). A potentially interesting direction for future work would be to develop an extension of iGecco+ that can appropriately handle block-missing multi-view data. Moreover, Weylandt et al. (2020) developed a fast algorithm to compute the entire convex clustering solution path and used this to visualize the results via a dendrogram and pathwise plot. In future work, we expect that algorithmic regularization path approaches can also be applied to our method to be able to represent our solution as a dendrogram and employ other dynamic visualizations. Finally, while we develop an efficient multi-block ADMM algorithm, there may be further room to speed up computation of iGecco+, potentially by using distributed optimization approaches.

In this paper, we demonstrate that our method can be applied to integrative genomics, yet it can be applied to other fields such as multi-modal imaging, national security, online advertising, and environmental studies where practitioners aim to find meaningful clusters and features at the same time. In conclusion, we introduce a principled, unified approach to a challenging problem that demonstrates strong empirical performance and opens many directions for future research.

Our method is implemented in MATLAB and available at https://github.com/DataSlingers/iGecco.

## Figures and Tables

**Table 1:** Different losses and their loss-specific centers. We provide all calculations associated with loss-specific centers in Appendix F. Note the Gecco problem with Hamming or Canberra distances is not convex. Though we discuss general convex losses in this paper, we list these non-convex losses for reference. For multinomial log-likelihood and multinomial deviance, we change Gecco formulation slightly to accommodate three indices; we provide a detailed formulation in Appendix E.

Data Type	Loss Type	Loss Function	Loss-specific Center $\tilde{x}$
Continuous	Euclidean (*ℓ*_2_)	$\frac{1}{2}{\left\|{\text{x}}_{i}-{\text{u}}_{i}\right\|}_{2}^{2}$	$\bar{x}$
Skewed continuous	Manhattan (*ℓ*_1_) Minkowski (*ℓ_q_*) Mahalanobis (weighted *ℓ*_2_) Chebychev (*ℓ*_∞_) Canberra (weighted *ℓ*_1_)	$\sum_{j=1}\left|x_{ij}-u_{ij}\right|$ $\sqrt[{q}]{\sum_{j=1}{\left|x_{ij}-u_{ij}\right|}^{q}}$ ${\left({\text{x}}_{i}-{\text{u}}_{i}\right)}^{T}{\text{C}}^{-1}\left({\text{x}}_{i}-u_{i}\right)$ $\max_{j}\left\{\left|x_{ij}-u_{ij}\right|\right\}$ $\sum_{j=1}\frac{\left|x_{ij}-u_{ij}\right|}{\left|x_{ij}\right|+\left|u_{ij}\right|}$	median(**x**) no closed form no closed form no closed form no closed form
Binary	Bernoulli log-likelihood Binomial deviance Hinge loss KL divergence Hamming (*ℓ*_0_)	$-x_{ij}u_{ij}+\log\left(1+e^{u_{ij}}\right)$ $-x_{ij}\log u_{ij}-\left(1-x_{ij}\right)\log\left(1-u_{ij}\right)$ $\max\left(0,1-u_{ij}x_{ij}\right)$ $-x_{ij}\log_{2}u_{ij}$ $\sum_{j}\#\left(x_{ij}\neq u_{ij}\right)/n$	logit($\bar{x}$) $\bar{x}$ mode(**x**) no closed form mode (**x**)
Count	Poisson log-likelihood Poisson deviance Negative binomial log-likelihood Negative binomial deviance Manhattan (*ℓ*_1_) Canberra (weighted *ℓ*_1_)	$-x_{ij}u_{ij}+\exp\left(u_{ij}\right)$ $-x_{ij}\log u_{ij}+u_{ij}$ $-x_{ij}u_{ij}+\left(x_{ij}+\frac{1}{\alpha }\right)\log\left(\frac{1}{\alpha }+e^{u_{ij}}\right)$ $x_{ij}\log\left(\frac{x_{ij}}{u_{ij}}\right)-\left(x_{ij}+\frac{1}{\alpha }\right)\log\left(\frac{1+\alpha x_{ij}}{1+\alpha u_{ij}}\right)$ $\sum_{j=1}\left|x_{ij}-u_{ij}\right|$ $\sum_{j=1}\frac{\left|x_{ij}-u_{ij}\right|}{\left|x_{ij}\right|+\left|u_{i}\right|}$	log($\bar{x}$) $\bar{x}$ log($\bar{x}$) $\bar{x}$ median(**x**) no closed form
Categorical	Multinomial log-likelihood Multinomial deviance	$\left\{\sum_{k=1}^{K}-x_{ijk}u_{ijk}+\log\left(\sum_{k=1}^{K}e^{u_{ijk}}\right)\right\}$ $\left\{\sum_{k=1}^{K}-x_{ijk}+\log\left(u_{ijk}\right)\right\},\sum_{k=1}^{K}u_{ijk}=1$	mlogit($\bar{x}$) $\bar{x}$

**Table 2:** Comparisons of F_1_ score for adaptive Gecco+ and sparse convex clustering

Method	Scenario 1 (A)	Scenario 2	Scenario 3
Sparse Convex Clustering	0.37 (3.1e-2)	0.25 (2.4e-2)	0.14 (7.2e-3)
Adaptive Gecco+	0.97 (1.9e-2)	0.99 (1.0e-2)	0.81 (8.0e-2)

**Table 3:** Comparisons of adjusted Rand index for mixed multi-view data

Method	Scenario 1	Scenario 2

Hclust: **X**_1_	0.35 (2.9e-2)	0.53 (2.3e-2)
Hclust: **X**_2_	0.53 (4.6e-2)	0.65 (1.8e-2)
Hclust: **X**_3_	0.52 (2.2e-2)	0.70 (2.4e-2)
Hclust: [**X**_1_**X**_2_**X**_3_] - Euclidean	0.68 (4.7e-2)	0.63 (3.3e-2)
Hclust: [**X**_1_**X**_2_**X**_3_] - Gower	0.86 (1.5e-2)	0.83 (7.3e-2)
iCluster+ with λ = 0	0.90 (1.6e-2)	0.71 (1.6e-2)
Bayesian Consensus Clustering	**0.95 (1.2e-2)**	0.63 (1.1e-2)
iGecco	0.93 (5.0e-3)	**0.98 (2.2e-2)**

**Table 4:** Comparisons of adjusted Rand index for high-dimensional mixed multi-view data

Method	Scenario 3	Scenario 4	Scenario 5	Scenario 6

Hclust: **X**_1_	0.42 (2.3e-2)	0.56 (2.5e-2)	0.43 (2.5e-2)	0.51 (2.7e-2)
Hclust: **X**_2_	0.23 (2.8e-2)	0.29 (3.4e-2)	0.51 (2.6e-2)	0.49 (2.1e-2)
Hclust: **X**_3_	0.25 (3.1e-2)	0.27 (3.1e-2)	0.55 (2.6e-2)	0.48 (1.9e-2)
Hclust: [**X**_1_**X**_2_**X**_3_] - Euclidean	0.40 (3.7e-2)	0.57 (3.3e-2)	0.55 (2.5e-2)	0.52 (2.1e-2)
Hclust: [**X**_1_**X**_2_**X**_3_] - Gower	0.68 (3.4e-2)	0.58 (6.3e-2)	0.58 (3.2e-2)	0.58 (3.0e-2)
iCluster+	0.57 (6.5e-2)	0.77 (2.7e-2)	0.61 (2.4e-2)	0.62 (1.6e-2)
Bayesian Consensus Clustering	0.35 (1.1e-1)	0.64 (1.0e-1)	0.59 (1.2e-2)	0.63 (6.6e-3)
iGecco	0.00 (6.7e-4)	0.06 (5.0e-2)	0.39 (4.5e-2)	0.23 (6.9e-2)
iGecco+	0.12 (3.3e-2)	0.16 (7.1e-2)	0.44 (3.6e-2)	0.39 (3.8e-2)
Adaptive iGecco+	**0.97 (7.8e-3)**	**0.99 (7.5e-3)**	**1.00 (0.0e-0)**	**1.00 (0.0e-0)**

**Table 5:** Comparisons of F_1_ score for adaptive iGecco+ and iClusterPlus

	Overall		Gaussian		Count		Binary	
	iCluster+	A iGecco+	iCluster+	A iGecco+	iCluster+	A iGecco+	iCluster+	A iGecco+
S3	0.81 (3.1e-2)	**0.94 (1.7e-2)**	0.84 (5.7e-2)	0.99 (6.3e-3)	0.73 (3.3e-2)	0.88 (3.5e-2)	0.85 (1.5e-2)	0.93 (2.1e-2)
S4	0.95 (9.9e-3)	**0.98 (1.3e-2)**	0.99 (6.7e-3)	0.99 (7.3e-3)	0.92 (1.3e-2)	0.97 (1.8e-2)	0.94 (1.9e-2)	0.97 (1.8e-2)
S5	0.94 (3.5e-2)	**1.00 (0.0e-0)**	0.95 (3.3e-2)	1.00 (0.0e-0)	0.91 (4.2e-2)	1.00 (0.0e-0)	0.95 (3.3e-2)	1.00 (0.0e-0)
S6	0.92 (3.3e-2)	**1.00 (3.3e-3)**	0.97 (2.1e-2)	1.00 (0.0e-0)	0.84 (4.5e-2)	0.99 (1.0e-2)	0.95 (3.3e-2)	1.00 (0.0e-0)

**Table 6:** Adjusted Rand index of different methods for authors data set

Method	Adjusted Rand Index

K-means	0.74
Hierarchical Clustering	0.73
Sparse Convex Clustering	0.50
Manhattan Gecco+	0.96
Poisson LL Gecco+	0.96
Poisson Deviance Gecco+	0.96

**Table 7:** Features selected by different Gecco+ methods for authors data set

Method	Features
Manhattan Gecco+	“be” ,“had” ,“her”, “the” ,“to”, “was”
Poisson LL Gecco+	“an” , “her” , “our”, “your”
Poisson Deviance Gecco+	“an”, “be” , “had”, “her”, “is”, “my” , “the”, “was”

**Table 8:** Adjusted Rand index of different methods for TCGA data set

Method	Adjusted Rand Index

K-means	0.40
Hierarchical Clustering	0.37
Sparse Convex Clustering	0.01
Manhattan Gecco+	0.76
Poisson LL Gecco+	0.72
Poisson Deviance Gecco+	0.72

**Table 9:** Features selected by different Gecco+ methods for TCGA data set

Method	Features
Manhattan Gecco+	“BCL2” , “ERBB2” ,“GATA3” “HMGA1”, “IL6ST”
Poisson LL Gecco+	“ERBB2” “FOXA1” “GATA3”
Poisson Deviance Gecco+	“ERBB2” , “FOXA1”, “GATA3” “RET”, “SLC34A2”

**Table 10:** Adjusted Rand index of different methods for multi-omics TCGA data set

Method	Adjusted Rand Index

Hclust: **X**_1_ GE	0.51
Hclust: **X**_2_ Meth	0.39
Hclust: **X**_3_ miRNA	0.21
Hclust: **X**_4_ Protein	0.24
Hclust: [**X**_1_**X**_2_**X**_3_**X**_4_] - Euclidean	0.51
Hclust: [**X**_1_**X**_2_**X**_3_**X**_4_] - Gower	0.40
iCluster+	0.36
Bayesian Consensus Clustering	0.35
Adaptive iGecco+	**0.71**

**Table 11:** Features selected by adaptive iGecco+ methods for multi-omics TCGA data set

Data view	Features
Gene Expression	“AGR3”, “FOXA1”, “AGR2”, “ROPN1”, “ROPN1B”, “ESR1”, “C1orf64”, “ART3”,“FSIP1”
miRNA	“hsa-mir-135b”, “hsa-mir-190b”, “hsa-mir-577”, “hsa-mir-934”
Methylation	“cg08047457”, “cg08097882”, “cg00117172”, “cg12265829”
Protein	“ER.alpha”, “GATA3”, “AR”, “CyclimE1”

**Figure 5:**
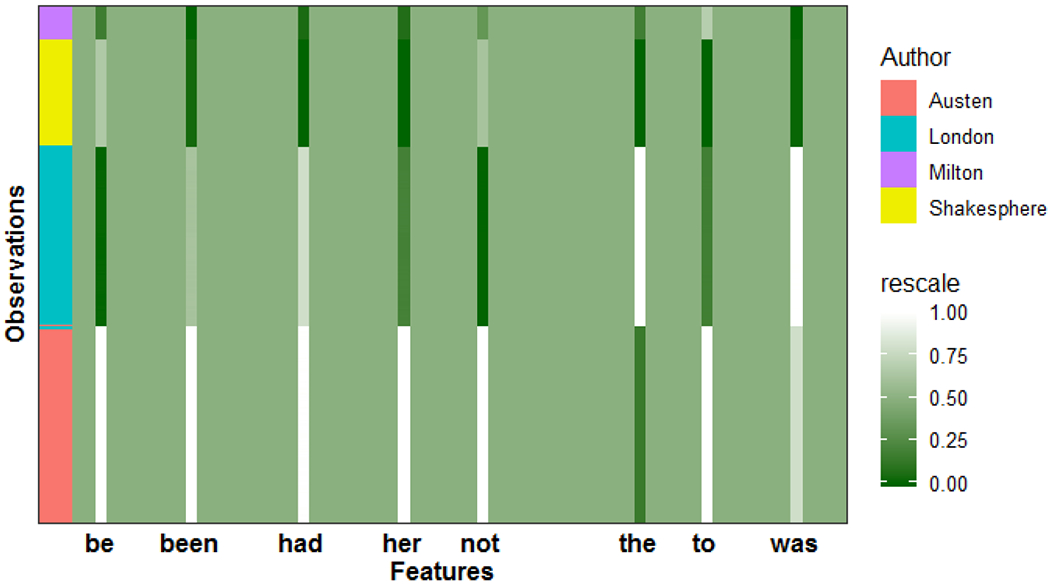
Cluster heatmap of Gecco+ solution $\hat{U}(\gamma ,\alpha )$ for authors data set with *α* = 15 and *γ* = 10^5^. The left bar refers to the true author label. We highlight selected features at the bottom. Gecco+ selects informative features that separate groups.

## Appendix A. Proof of Propositions

Proposition 1 and 2 are direct extensions from Proposition 2.1 of Chi and Lange (2015). Notice they proved the solution path depends continuously on the tuning parameter *γ* and the weight matrix **w**. It follows that the argument can be also applied to tuning parameter *α*, the loss weight *π_k_*, and feature weight $\zeta_{j}^{\left(k\right)}$. Also it is obvious that the loss ℓ(⋅) is continuous with respect to the data, **X**.

We show in detail how to prove Proposition 3 in the following. First we rewrite the objective $F_{\gamma ,\alpha }\left(U\right)$ as:

$$\begin{array}{l} F_{\gamma ,\alpha }\left(U\right)=\sum_{k=1}^{K}\pi_{k}\ell_{k}\left(X^{\left(k\right)},U^{\left(k\right)}\right)+\gamma \sum_{i<i^{\prime }}w_{ii^{\prime }}\sqrt{\sum_{k=1}^{K}{\left\|U_{i.}^{\left(k\right)}-U_{i^{\prime }.}^{\left(k\right)}\right\|}_{2}^{2}}+\alpha \sum_{k=1}^{K}\sum_{j=1}^{p_{k}}\zeta_{j}^{\left(k\right)}{\left\|U_{.j}^{\left(k\right)}-{\tilde{x}}_{j}^{\left(k\right)}\cdot 1_{n}\right\|}_{2}\\ \;\;\;=\sum_{k=1}^{K}\sum_{i=1}^{n}\pi_{k}\ell_{k}\left(X_{i.}^{\left(k\right)},U_{i.}^{\left(k\right)}\right)+\gamma \sum_{i<i^{\prime }}w_{ii^{\prime }}\sqrt{\sum_{k=1}^{K}{\left\|U_{i.}^{\left(k\right)}-U_{i^{\prime }.}^{\left(k\right)}\right\|}_{2}^{2}}+\alpha \sum_{k=1}^{K}\sum_{j=1}^{p_{k}}\zeta_{j}^{\left(k\right)}{\left\|U_{.j}^{\left(k\right)}-{\tilde{x}}_{j}^{\left(k\right)}\cdot 1_{n}\right\|}_{2}. \end{array}$$

By definition, loss-specific cluster center is ${\tilde{x}}^{\left(k\right)}=\underset{u}{\mathrm{argmin}}\sum_{i=1}^{n}\ell_{k}\left(X_{i.}^{\left(k\right)},u\right)$. Since *ℓ_k_* is convex, it is equivalent to **u** such that $\partial \sum_{i}\ell_{k}\left(X_{i.}^{\left(k\right)},u\right)=0$. Hence, $\partial \sum_{i}\ell_{k}\left(X_{i.}^{\left(k\right)},{\tilde{x}}^{\left(k\right)}\right)=0$.

We use the similar proof approach of Chi and Lange (2015). A point **X** furnishes a global minimum of the convex function F(X) if and only if all forward directional derivatives $d_{\Theta }F\left(X\right)$ at **X** are nonnegative. Here $\Theta =\left\{\Theta^{\left(1\right)},\cdots ,\Theta^{\left(K\right)}\right\}$ represents a direction in the space of possible concatenated centroids, where $\Theta^{\left(k\right)}\in R^{n\times p_{k}}$. We calculate the directional derivatives:

$$\begin{array}{l} d_{\Theta }F_{\gamma ,\alpha }\left(\tilde{X}\right)=\sum_{k=1}^{K}\sum_{i=1}^{n}\pi_{k}\langle \partial \ell_{k}\left(X_{i.}^{\left(k\right)},{\tilde{x}}^{\left(k\right)}\right),\Theta_{i.}^{\left(k\right)}\rangle +\gamma \sum_{i<i^{\prime }}w_{ii^{\prime }}\sqrt{\sum_{k=1}^{K}{\left\|\Theta_{i.}^{\left(k\right)}-\Theta_{i^{\prime }.}^{\left(k\right)}\right\|}_{2}^{2}}\\ \;\;\;\;+\alpha \sum_{k=1}^{K}\sum_{j=1}^{p_{k}}\zeta_{j}^{\left(k\right)}{\left\|\Theta_{.j}^{\left(k\right)}-{\tilde{x}}_{j}^{\left(k\right)}\cdot 1_{n}\right\|}_{2}. \end{array}$$

Note $\sum_{i=1}^{n}\langle \partial \ell_{k}\left(X_{i.}^{\left(k\right)},{\tilde{x}}^{\left(k\right)}\right),\Theta_{i^{\prime }.}^{\left(k\right)}\rangle =0$. The generalized Cauchy-Schwartz inequality therefore implies:

$$\begin{array}{l} \sum_{i=1}^{n}\langle \partial \ell_{k}\left(X_{i.}^{\left(k\right)},{\tilde{x}}^{\left(k\right)}\right),\Theta_{i.}^{\left(k\right)}\rangle =\frac{1}{n}\sum_{i=1}^{n}\sum_{i^{\prime }=1}^{n}\langle \partial \ell_{k}\left(X_{i.}^{\left(k\right)},{\tilde{x}}^{\left(k\right)}\right),\Theta_{i.}^{\left(k\right)}\rangle \\ \;\;\;\;\;\;\;\;\;\;\;\;=\frac{1}{n}\sum_{i=1}^{n}\sum_{i^{\prime }=1}^{n}\langle \partial \ell_{k}\left(X_{i.}^{\left(k\right)},{\tilde{x}}^{\left(k\right)}\right),\Theta_{i.}^{\left(k\right)}-\Theta_{i^{\prime }.}^{\left(k\right)}\rangle \\ \;\;\;\;\;\;\;\;\;\;\;\;\geq -\frac{2}{n}\sum_{i<i^{\prime }}{\left\|\partial \ell_{k}\left(X_{i.}^{\left(k\right)},{\tilde{x}}^{\left(k\right)}\right)\right\|}_{2}\cdot {\left\|\Theta_{i.}^{\left(k\right)}-\Theta_{i^{\prime }.}^{\left(k\right)}\right\|}_{2}\\ \;\;\;\;\;\;\;\;\;\;\;\;\geq -\frac{2}{n}\sum_{i<i^{\prime }}{\left\|\partial \ell_{k}\left(X_{i.}^{\left(k\right)},{\tilde{x}}^{\left(k\right)}\right)\right\|}_{2}\cdot \sqrt{\sum_{k=1}^{K}{\left\|\Theta_{i.}^{\left(k\right)}-\Theta_{i^{\prime }.}^{\left(k\right)}\right\|}_{2}^{2}}. \end{array}$$

Hence, we have:

$$\sum_{k=1}^{K}\sum_{i=1}^{n}\pi_{k}\langle \partial \ell_{k}\left(X_{i.}^{\left(k\right)},{\tilde{x}}^{\left(k\right)}\right),\Theta_{i.}^{\left(k\right)}\rangle \geq -\frac{2}{n}\sum_{k=1}^{K}\sum_{i<i^{\prime }}\pi_{k}{\left\|\partial \ell_{k}\left(X_{i.}^{\left(k\right)},{\tilde{x}}^{\left(k\right)}\right)\right\|}_{2}\cdot \sqrt{\sum_{k=1}^{K}{\left\|\Theta_{i.}^{\left(k\right)}-\Theta_{i^{\prime }.}^{\left(k\right)}\right\|}_{2}^{2}}.$$

Take *γ* sufficiently large such that:

$$\gamma \sum_{i<i^{\prime }}w_{ii^{\prime }}\sqrt{\sum_{k=1}^{K}{\left\|\Theta_{i.}^{\left(k\right)}-\Theta_{i^{\prime }.}^{\left(k\right)}\right\|}_{2}^{2}}\geq -\frac{2}{n}\sum_{k=1}^{K}\sum_{i<i^{\prime }}\pi_{k}{\left\|\partial \ell_{k}\left(X_{i.}^{\left(k\right)},{\tilde{x}}^{\left(k\right)}\right)\right\|}_{2}\cdot \sqrt{\sum_{k=1}^{K}{\left\|\Theta_{i.}^{\left(k\right)}-\Theta_{i^{\prime }.}^{\left(k\right)}\right\|}_{2}^{2}}.$$

When all $w_{ii^{\prime }}>0$, one can take any *γ* that exceeds

$$K\cdot \frac{2}{n}\underset{i,i^{\prime },k}{\max}\frac{\pi_{k}{\left\|\partial \ell_{k}\left(X_{i.}^{\left(k\right)},{\tilde{x}}^{\left(k\right)}\right)\right\|}_{2}}{w_{ii^{\prime }}}.$$

In general set

$$\beta =K\cdot \frac{2}{n\;\min_{w_{ii^{\prime }}>0}w_{ii^{\prime }}}\underset{i,i^{\prime },k}{\max}\pi_{k}{\left\|\partial \ell_{k}\left(X_{i}^{\left(k\right)},{\tilde{x}}^{\left(k\right)}\right)\right\|}_{2}.$$

For any pair *i* and *i′* there exists a path $i\to k\to \cdots \to l\to i^{\prime }$ along which the weights are positive. It follows that

$$\frac{2}{n}\sum_{k=1}^{K}\pi_{k}{\left\|\partial \ell_{k}\left(X_{i.}^{\left(k\right)},{\tilde{x}}^{\left(k\right)}\right)\right\|}_{2}\cdot \sqrt{\sum_{k=1}^{K}{\left\|\Theta_{i.}^{\left(k\right)}-\Theta_{i^{\prime }.}^{\left(k\right)}\right\|}_{2}^{2}}\leq \beta \sum_{i<i^{\prime }}w_{ii^{\prime }}\sqrt{\sum_{k=1}^{K}{\left\|\Theta_{i.}^{\left(k\right)}-\Theta_{i^{\prime }.}^{\left(k\right)}\right\|}_{2}^{2}}.$$

We have

$$\frac{2}{n}\sum_{k=1}^{K}\sum_{i<i^{\prime }}\pi_{k}{\left\|\partial \ell_{k}\left(X_{i.}^{\left(k\right)},{\tilde{x}}^{\left(k\right)}\right)\right\|}_{2}\cdot \sqrt{\sum_{k=1}^{K}{\left\|\Theta_{i.}^{\left(k\right)}-\Theta_{i^{\prime }.}^{\left(k\right)}\right\|}_{2}^{2}}\leq \left(\begin{array}{c} n\\ 2 \end{array}\right)\beta \sum_{i<i^{\prime }}w_{ii^{\prime }}\sqrt{\sum_{k=1}^{K}{\left\|\Theta_{i.}^{\left(k\right)}-\Theta_{i^{\prime }.}^{\left(k\right)}\right\|}_{2}^{2}}.$$

Hence the forward directional derivative test is satisfied for any $\gamma \geq \left(\begin{array}{c} n\\ 2 \end{array}\right)\beta$.

On the other hand, for fixed *γ*, the generalized Cauchy-Schwartz inequality implies:

$$\begin{array}{l} \sum_{i=1}^{n}\langle \partial \ell_{k}\left(X_{i.}^{\left(k\right)},{\tilde{x}}^{\left(k\right)}\right),\Theta_{i.}^{\left(k\right)}\rangle =\sum_{i=1}^{n}\langle \partial \ell_{k}\left(X_{i.}^{\left(k\right)},{\tilde{x}}^{\left(k\right)}\right),\Theta_{i.}^{\left(k\right)}-{\tilde{x}}^{\left(k\right)}\rangle \\ \;\;\;\;\;\;\;\;\;\;\;\;=\sum_{j=1}^{p_{k}}\langle \partial \ell_{k}\left(X_{.j}^{\left(k\right)},{\tilde{x}}_{j}^{\left(k\right)}\cdot 1_{n}\right),\Theta_{.j}^{\left(k\right)}-{\tilde{x}}_{j}^{\left(k\right)}\cdot 1_{n}\rangle \\ \;\;\;\;\;\;\;\;\;\;\;\;\geq -\sum_{j=1}^{p_{k}}{\left\|\partial \ell_{k}\left(X_{.j}^{\left(k\right)},{\tilde{x}}_{j}^{\left(k\right)}\cdot 1_{n}\right)\right\|}_{2}\cdot {\left\|\Theta_{.j}^{\left(k\right)}-{\tilde{x}}_{j}^{\left(k\right)}\cdot 1_{n}\right\|}_{2}. \end{array}$$

Hence, we have:

$$\sum_{k=1}^{K}\sum_{i=1}^{n}\pi_{k}\langle \partial \ell_{k}\left(X_{i.}^{\left(k\right)},{\tilde{x}}^{\left(k\right)}\right),\Theta_{i.}^{\left(k\right)}\rangle \geq -\sum_{k=1}^{K}\sum_{j=1}^{p_{k}}\pi_{k}{\left\|\partial \ell_{k}\left(X_{.j}^{\left(k\right)},{\tilde{x}}_{j}^{\left(k\right)}\cdot 1_{n}\right)\right\|}_{2}\cdot {\left\|\Theta_{.j}^{\left(k\right)}-{\tilde{x}}_{j}^{\left(k\right)}\cdot 1_{n}\right\|}_{2}.$$

Take *α* sufficiently large so that

$$\alpha \sum_{k=1}^{K}\sum_{j=1}^{p_{k}}\zeta_{j}^{\left(k\right)}{\left\|\Theta_{.j}^{\left(k\right)}-{\tilde{x}}_{j}^{\left(k\right)}\cdot 1_{n}\right\|}_{2}\geq -\sum_{k=1}^{K}\sum_{j=1}^{p_{k}}\pi_{k}{\left\|\partial \ell_{k}\left(X_{.j}^{\left(k\right)},{\tilde{x}}_{j}^{\left(k\right)}\cdot 1_{n}\right)\right\|}_{2}\cdot {\left\|\Theta_{.j}^{\left(k\right)}-{\tilde{x}}_{j}^{\left(k\right)}\cdot 1_{n}\right\|}_{2}.$$

When all $\zeta_{j}^{\left(k\right)}>0$, one can take any *α* that exceeds

$$\underset{j,k}{\max}\frac{\pi_{k}{\left\|\partial \ell_{k}\left(X_{.j}^{\left(k\right)},{\tilde{x}}_{j}^{\left(k\right)}\cdot 1_{n}\right)\right\|}_{2}}{\zeta_{j}^{\left(k\right)}}.$$

In general, set $\alpha \geq \frac{1}{\min_{\zeta_{j}^{\left(k\right)}>0}\zeta_{j}^{\left(k\right)}}\cdot \max_{j,k}\pi_{k}{\left\|\partial \ell_{k}\left(X_{.j}^{\left(k\right)},{\tilde{x}}_{j}^{\left(k\right)}\cdot 1_{n}\right)\right\|}_{2}$. It is easy to check the forward directional derivative test is satisfied. ■

## Appendix B. Proof of Theorem 4

Recall the iGecco+ problem is:

$$\begin{array}{l} \underset{U^{\left(k\right)}}{\min}\sum_{k=1}^{K}\pi_{k}\ell_{k}\left(X^{\left(k\right)},U^{\left(k\right)}\right)+\gamma \sum_{i<i^{\prime }}w_{ii^{\prime }}\sqrt{\sum_{k=1}^{K}{\left\|U_{i.}^{\left(k\right)}-U_{i^{\prime }.}^{\left(k\right)}\right\|}_{2}^{2}}\\ \;\;+\alpha \sum_{k=1}^{K}\sum_{j=1}^{p_{k}}\zeta_{j}^{\left(k\right)}{\left\|U_{.j}^{\left(k\right)}-{\tilde{x}}_{j}^{\left(k\right)}\cdot 1_{n}\right\|}_{2}. \end{array}$$

We can recast the original iGecco+ problem as the equivalent constrained problem:

(2)
$$\begin{array}{l} \underset{U^{\left(k\right)},V}{\mathrm{minimize}}\sum_{k=1}^{K}\pi_{k}\ell_{k}\left(X^{\left(k\right)},U^{\left(k\right)}\right)+\gamma \underset{P_{1}\left(V;w\right)}{\underbrace{\left(\sum_{l\in \varepsilon }w_{l}{\left\|V_{l.}\right\|}_{2}\right)}}\\ \;\;\;\;+\alpha \sum_{k=1}^{K}\sum_{j=1}^{p_{k}}\zeta_{j}^{\left(k\right)}{\left\|U_{.j}^{\left(k\right)}-{\tilde{x}}_{j}^{\left(k\right)}\cdot 1_{n}\right\|}_{2}+\sum_{k^{\prime }=1}^{K}\pi_{k^{\prime }}f_{k^{\prime }}\left(Z^{\left(k^{\prime }\right)}\right)+\alpha \sum_{k^{\prime }=1}^{K}\underset{P_{{}_{2}}\left(R^{\left(k^{\prime }\right)};\zeta^{\left(k^{\prime }\right)}\right)}{\underbrace{\left(\sum_{j=1}^{{p^{\prime }}_{k}}\zeta_{j}^{\left(k^{\prime }\right)}{\left\|r_{j}^{\left(k^{\prime }\right)}\right\|}_{2}\right)}}\\ \text{subject to}\;{\text{DU}}^{\left(k\right)}-V^{\left(k\right)}=0,\;DU^{\left(k^{\prime }\right)}-V^{\left(k^{\prime }\right)}=0,\;X^{\left(k^{\prime }\right)}-U^{\left(k^{\prime }\right)}=Z^{\left(k^{\prime }\right)},U^{\left(k^{\prime }\right)}-{\tilde{X}}^{\left(k^{\prime }\right)}=R^{\left(k^{\prime }\right)}\;, \end{array}$$

where *ℓ_k_* refers to the differentiable losses and *ℓ_k′_* refers to the non-differentiable losses. We use multi-block ADMM algorithm (Algorithm 6) to solve the problem above.

To prove convergence of Algorithm 5, we first show that multi-block ADMM Algorithm 6 converges to a global minimum. Then we show that we can proximal-linearize the sub-problems in the primal updates of Algorithm 6 with proved convergence and this is equivalent to Algorithm 5. Without loss of generality, we assume we have one differentiable loss $\ell_{1}\left(\cdot \right)$ and one non-differentiable distance-based loss $\ell_{2}\left(\cdot \right)$.

**Algorithm 6:** Multi-block ADMM to solve iGecco+

**while** not converged **do**
**for** all k=1,⋯,K **do**
$U^{\left(k\right)}=\underset{U}{\mathrm{argmin}}\;\pi_{k}\ell_{k}\left(X^{\left(k\right)},U\right)+\frac{\rho }{2}{\left\|DU-V^{\left(k\right)}+\Lambda^{\left(k\right)}\right\|}_{F}^{2}+\alpha \sum_{j=1}^{p_{k}}\zeta_{j}^{\left(k\right)}{\left\|U_{.j}-{\tilde{x}}_{j}^{\left(k\right)}\cdot 1_{n}\right\|}_{2}$
$U^{\left(k^{\prime }\right)}=\underset{U}{\mathrm{argmin}}\;\frac{\rho }{2}{\left\|X^{\left(k^{\prime }\right)}-U+Z^{\left(k^{\prime }\right)}+\Psi^{\left(k^{\prime }\right)}\right\|}_{F}^{2}+\frac{\rho }{2}{\left\|U-{\tilde{X}}^{\left(k^{\prime }\right)}-R^{\left(k^{\prime }\right)}+N^{\left(k^{\prime }\right)}\right\|}_{F}^{2}+\frac{\rho }{2}{\left\|DU-V^{\left(k^{\prime }\right)}+\Lambda^{\left(k^{\prime }\right)}\right\|}_{F}^{2}$
$Z^{\left(k^{\prime }\right)}=\underset{Z}{\mathrm{argmin}}\;\pi_{k^{\prime }}f_{k^{\prime }}\left(Z\right)+\frac{\rho }{2}{\left\|X^{\left(k^{\prime }\right)}-U^{\left(k^{\prime }\right)}-Z+\Psi^{\left(k^{\prime }\right)}\right\|}_{F}^{2}$
$R^{\left(k^{\prime }\right)}=\underset{R}{\mathrm{argmin}}\;\alpha \sum_{j=1}^{p_{k^{\prime }}}\zeta_{j}^{\left(k^{\prime }\right)}{\left\|U_{.j}^{\left(k^{\prime }\right)}-{\tilde{x}}_{j}^{\left(k^{\prime }\right)}\cdot 1_{n}\right\|}_{2}+\frac{\rho }{2}{\left\|U^{\left(k^{\prime }\right)}-{\tilde{X}}^{\left(k^{\prime }\right)}-R+N^{\left(k^{\prime }\right)}\right\|}_{F}^{2}$
$\Psi^{\left(k^{\prime }\right)}=\Psi^{\left(k^{\prime }\right)}+\left(X^{\left(k^{\prime }\right)}-U^{\left(k^{\prime }\right)}-Z^{\left(k^{\prime }\right)}\right)$
$N^{\left(k^{\prime }\right)}=N^{\left(k^{\prime }\right)}+\left(U^{\left(k^{\prime }\right)}-{\tilde{X}}^{\left(k^{\prime }\right)}-R^{\left(k^{\prime }\right)}\right)$
**end for**
$V=\underset{V}{\mathrm{argmin}}\frac{\rho }{2}{\left\|DU^{\left(k\right)}-V^{\left(k\right)}+\Lambda^{\left(k\right)}\right\|}_{F}^{2}+\frac{\rho }{2}{\left\|DU^{\left(k^{\prime }\right)}-V^{\left(k^{\prime }\right)}+\Lambda^{\left(k^{\prime }\right)}\right\|}_{F}^{2}+\gamma \left(\sum_{l\in \varepsilon }w_{l}{\left\|V_{l.}\right\|}_{2}\right)$
$\Lambda^{\left(k\right)}=\Lambda^{\left(k\right)}+\left(DU^{\left(k\right)}-V^{\left(k\right)}\right)$ for all *k* and *k′*
**end while**

To prove convergence of Algorithm 6, we first propose a sufficient condition for the convergence of four-block ADMM and prove it holds true. This is an extension of the convergence results in Section 2 of the work by Chen et al. (2016). Suppose the convex optimization problem with linear constraints we want to minimize is

(3)
$$\begin{array}{l} \min\;\theta_{1}\left(x_{1}\right)+\theta_{2}\left(x_{2}\right)+\theta_{3}\left(x_{3}\right)+\theta_{4}\left(x_{4}\right)\\ \;\text{s.t.}\;A_{1}x_{1}+A_{2}x_{2}+A_{3}x_{3}+A_{4}x_{4}=b. \end{array}$$

The multi-block ADMM has the following form. Note here, the superscript in $x_{i}^{\left(k+1\right)}$ refers to the (*k* + 1)^*th*^ iteration in the ADMM updates. We have:

(4)
$$\{\begin{array}{l} x_{1}^{\left(k+1\right)}=\mathrm{argmin}\left\{L_{A}\left(x_{1},x_{2}^{\left(k\right)},x_{3}^{\left(k\right)},x_{4}^{\left(k\right)},\lambda^{\left(k\right)}\right)\right\}\\ x_{2}^{\left(k+1\right)}=\mathrm{argmin}\left\{L_{A}\left(x_{1}^{\left(k+1\right)},x_{2},x_{3}^{\left(k\right)},x_{4}^{\left(k\right)},\lambda^{\left(k\right)}\right)\right\}\\ x_{3}^{\left(k+1\right)}=\mathrm{argmin}\left\{L_{A}\left(x_{1}^{\left(k+1\right)},x_{2}^{\left(k+1\right)},x_{3},x_{4}^{\left(k\right)},\lambda^{\left(k\right)}\right)\right\}\\ x_{4}^{\left(k+1\right)}=\mathrm{argmin}\left\{L_{A}\left(x_{1}^{\left(k+1\right)},x_{2}^{\left(k+1\right)},x_{3}^{\left(k+1\right)},x_{4},\lambda^{\left(k\right)}\right)\right\}\\ \lambda^{\left(k+1\right)}=\lambda^{\left(k\right)}-\left(A_{1}x_{1}^{\left(k+1\right)}+A_{2}x_{2}^{\left(k+1\right)}+A_{3}x_{3}^{\left(k+1\right)}+A_{4}x_{4}^{\left(k+1\right)}-b\right)\;, \end{array}$$

where

$$\begin{array}{l} L_{A}=\sum_{i=1}^{4}\theta_{i}\left(x_{i}\right)-\lambda^{T}\left(A_{1}x_{1}+A_{2}x_{2}+A_{3}x_{3}+A_{4}x_{4}-b\right)\\ \;\;\;\;\;\;\;+\frac{1}{2}{\left\|A_{1}x_{1}+A_{2}x_{2}+A_{3}x_{3}+A_{4}x_{4}-b\right\|}_{2}^{2}. \end{array}$$

We establish Lemma 5, a sufficient condition for convergence of four-block ADMM:

**Lemma 5** *(Sufficient Condition for Convergence of Four-block ADMM) A sufficient condition ensuring the convergence of* (4) *to a global solution of* (3) *is:* $A_{2}^{T}A_{3}=0$, $A_{2}^{T}A_{4}=0$, $A_{3}^{T}A_{4}=0$.

We prove Lemma 5 at the end of this section. Note that Lemma 5 is stated in vector form and therefore we need to transform the constraints in the original iGecco+ problem (2) from matrix form to vector form in order to apply Lemma 5. Note that $DU^{\left(k\right)}=V^{\left(k\right)}\Leftrightarrow {U^{\left(k\right)}}^{T}D^{T}=V^{{\left(k\right)}^{T}}\Leftrightarrow \left(D⊗I_{p_{{}_{k}}}\right)\text{vec}\left(U^{{\left(k\right)}^{T}}\right)=\text{vec}\left(V^{{\left(k\right)}^{T}}\right)$. Hence we can write the constraints in (2) as:

$$\left(\begin{array}{cc} {\text{A}}_{1} & 0\\ 0 & \text{I}\\ 0 & {\text{A}}_{2}\\ 0 & \text{I} \end{array}\right)\text{u}+\left(\begin{array}{c} 0\\ \text{I}\\ 0\\ 0 \end{array}\right)\text{z}+\left(\begin{array}{c} 0\\ 0\\ 0\\ -\text{I} \end{array}\right)\text{r}+\left(\begin{array}{cc} -I & 0\\ 0 & 0\\ 0 & -\text{I}\\ 0 & 0 \end{array}\right)\text{v}=\text{b}\;,$$

where $u=\left(\begin{array}{c} u_{1}\\ u_{2} \end{array}\right)=\left(\begin{array}{c} \text{vec}\left(U^{{\left(1\right)}^{T}}\right)\\ \text{vec}\left(U^{{\left(2\right)}^{T}}\right) \end{array}\right)$, $A_{1}=D⊗I_{p_{1}}$, $A_{2}=D⊗I_{p_{2}}$, $z=\text{vec}\left(Z^{T}\right)$, $\text{r}=\text{vec}\left(R^{T}\right)$, $\text{v}=\text{vec}\left(V^{T}\right)=\left(\begin{array}{c} \text{vec}\left(V^{{\left(1\right)}^{T}}\right)\\ \text{vec}\left(V^{{\left(2\right)}^{T}}\right) \end{array}\right)$, $\text{b}=\left(\begin{array}{c} 0_{p_{1}\times \left|\varepsilon \right|}\\ \text{vec}\left(X^{{\left(2\right)}^{T}}\right)\\ 0_{p_{2}\times \left|\varepsilon \right|}\\ {\tilde{x}}^{\left(2\right)}\\ \vdots \\ {\tilde{x}}^{\left(2\right)} \end{array}\right).\tilde{x}\in R^{p_{2}}$ is a column vector consisting of all ${\tilde{x}}_{j}^{2}$ and is repeated *n* times in **b**.

Next we show that the constraints in (2) for our problem satisfy the condition in Lemma 5 and hence the multi-block ADMM Algorithm 6 converges.

By construction, $E_{2}=\left(\begin{array}{c} 0\\ \text{I}\\ 0\\ 0 \end{array}\right)$, $E_{3}=\left(\begin{array}{c} 0\\ 0\\ 0\\ -\text{I} \end{array}\right)$ and $E_{4}=\left(\begin{array}{cc} -\text{I} & 0\\ 0 & 0\\ 0 & -\text{I}\\ 0 & 0 \end{array}\right)$. It is easy to verify that: ${\text{E}}_{2}^{T}E_{3}=0$, ${\text{E}}_{2}^{T}E_{4}=0$, ${\text{E}}_{3}^{T}E_{4}=0$. Hence our setup satisfies the sufficient condition in Lemma 5 and the multi-block ADMM Algorithm 6 converges.

Next, we see that each primal update in Algorithm 5 is equivalent to the primal update by applying proximal linearized ADMM to the sub-problems in Algorithm 6. (We will show this in detail in Theorem 6.) It is easy to show that these updates with closed-form solutions are special cases of proximal-linearizing the sub-problems. Meanwhile, Lu et al. (2016); Liu et al. (2013) showed the convergence of proximal linearized multi-block ADMM. Hence Algorithm 5 converges to a global minimum if *ℓ_k_* is convex for all *k* and has Lipschitz gradient when it is differentiable. Further, if each *ℓ_k_* is strictly convex, it converges to the unique global solution. ■

Proof of Lemma 5:

According to the first-order optimality conditions of the minimization problems in (4), we have:

$$\{\begin{array}{l} \theta_{1}\left({\text{x}}_{1}\right)-\theta_{1}\left({\text{x}}_{1}^{\left(k+1\right)}\right)+{\left({\text{x}}_{1}-{\text{x}}_{1}^{\left(k+1\right)}\right)}^{T}\left\{-A_{1}^{T}\left[\lambda^{\left(k\right)}-\left(A_{1}{\text{x}}_{1}^{\left(k+1\right)}+A_{2}{\text{x}}_{2}^{\left(k\right)}+A_{3}{\text{x}}_{3}^{\left(k\right)}+A_{4}{\text{x}}_{4}^{\left(k\right)}-\text{b}\right)\right]\right\}\geq 0\\ \theta_{2}\left({\text{x}}_{2}\right)-\theta_{2}\left({\text{x}}_{2}^{\left(k+1\right)}\right)+{\left({\text{x}}_{2}-{\text{x}}_{2}^{\left(k+1\right)}\right)}^{T}\left\{-A_{2}^{T}\left[\lambda^{\left(k\right)}-\left(A_{1}{\text{x}}_{1}^{\left(k+1\right)}+A_{2}{\text{x}}_{2}^{\left(k+1\right)}+A_{3}{\text{x}}_{3}^{\left(k\right)}+A_{4}{\text{x}}_{4}^{\left(k\right)}-\text{b}\right)\right]\right\}\geq 0\\ \theta_{3}\left({\text{x}}_{3}\right)-\theta_{3}\left({\text{x}}_{3}^{\left(k+1\right)}\right)+{\left({\text{x}}_{3}-{\text{x}}_{3}^{\left(k+1\right)}\right)}^{T}\left\{-A_{3}^{T}\left[\lambda^{\left(k\right)}-\left(A_{1}{\text{x}}_{1}^{\left(k+1\right)}+A_{2}{\text{x}}_{2}^{\left(k+1\right)}+A_{3}{\text{x}}_{3}^{\left(k+1\right)}+A_{4}{\text{x}}_{4}^{\left(k\right)}-\text{b}\right)\right]\right\}\geq 0\\ \theta_{4}\left({\text{x}}_{4}\right)-\theta_{4}\left({\text{x}}_{4}^{\left(k+1\right)}\right)+{\left({\text{x}}_{4}-{\text{x}}_{4}^{\left(k+1\right)}\right)}^{T}\left\{-A_{4}^{T}\left[\lambda^{\left(k\right)}-\left(A_{1}{\text{x}}_{1}^{\left(k+1\right)}+A_{2}{\text{x}}_{2}^{\left(k+1\right)}+A_{3}{\text{x}}_{3}^{\left(k+1\right)}+A_{4}{\text{x}}_{4}^{\left(k+1\right)}-\text{b}\right)\right]\right\}\geq 0. \end{array}$$

Since ${\text{A}}_{2}^{T}A_{3}=0$, ${\text{A}}_{2}^{T}A_{4}=0$, ${\text{A}}_{3}^{T}A_{4}=0$, we have:

$$\{\begin{array}{l} \theta_{1}\left({\text{x}}_{1}\right)-\theta_{1}\left({\text{x}}_{1}^{\left(k+1\right)}\right)+{\left({\text{x}}_{1}-{\text{x}}_{1}^{\left(k+1\right)}\right)}^{T}\left\{-A_{1}^{T}\left[\lambda^{\left(k\right)}-\left(A_{1}{\text{x}}_{1}^{\left(k+1\right)}+A_{2}{\text{x}}_{2}^{\left(k\right)}+A_{3}{\text{x}}_{3}^{\left(k\right)}+A_{4}{\text{x}}_{4}^{\left(k\right)}-\text{b}\right)\right]\right\}\geq 0\\ \theta_{2}\left({\text{x}}_{2}\right)-\theta_{2}\left({\text{x}}_{2}^{\left(k+1\right)}\right)+{\left({\text{x}}_{2}-{\text{x}}_{2}^{\left(k+1\right)}\right)}^{T}\left\{-A_{2}^{T}\left[\lambda^{\left(k\right)}-\left(A_{1}{\text{x}}_{1}^{\left(k+1\right)}+A_{2}{\text{x}}_{2}^{\left(k+1\right)}-\text{b}\right)\right]\right\}\geq 0\\ \theta_{3}\left({\text{x}}_{3}\right)-\theta_{3}\left({\text{x}}_{3}^{\left(k+1\right)}\right)+{\left({\text{x}}_{3}-{\text{x}}_{3}^{\left(k+1\right)}\right)}^{T}\left\{-A_{3}^{T}\left[\lambda^{\left(k\right)}-\left(A_{1}{\text{x}}_{1}^{\left(k+1\right)}+A_{3}{\text{x}}_{3}^{\left(k+1\right)}-\text{b}\right)\right]\right\}\geq 0\\ \theta_{4}\left({\text{x}}_{4}\right)-\theta_{4}\left({\text{x}}_{4}^{\left(k+1\right)}\right)+{\left({\text{x}}_{4}-{\text{x}}_{4}^{\left(k+1\right)}\right)}^{T}\left\{-A_{4}^{T}\left[\lambda^{\left(k\right)}-\left(A_{1}{\text{x}}_{1}^{\left(k+1\right)}+A_{4}{\text{x}}_{4}^{\left(k+1\right)}-\text{b}\right)\right]\right\}\geq 0, \end{array}$$

which is also the first-order optimality condition of the scheme:

(5)
$$\{\begin{array}{l} {\text{x}}_{1}^{\left(k+1\right)}=\mathrm{argmin}\left\{\theta_{1}\left({\text{x}}_{1}\right)-{\left(\lambda^{\left(k\right)}\right)}^{T}\left(A_{1}{\text{x}}_{1}\right)+\frac{1}{2}{\left\|A_{1}{\text{x}}_{1}+A_{2}{\text{x}}_{2}^{\left(k\right)}+A_{3}{\text{x}}_{3}^{\left(k\right)}+A_{4}{\text{x}}_{4}^{\left(k\right)}-\text{b}\right\|}_{2}^{2}\right\}\\ \left({\text{x}}_{2}^{\left(k+1\right)},{\text{x}}_{3}^{\left(k+1\right)},{\text{x}}_{4}^{\left(k+1\right)}\right)=\mathrm{argmin}\{\theta_{2}\left({\text{x}}_{2}\right)+\theta_{3}\left({\text{x}}_{3}\right)+\theta_{4}\left({\text{x}}_{4}\right)-{\left(\lambda^{\left(k\right)}\right)}^{T}\left({\text{A}}_{\text{2}}{\text{x}}_{\text{2}}+{\text{A}}_{3}{\text{x}}_{3}+{\text{A}}_{4}{\text{x}}_{4}\right)\\ \;\;\;\;\;\;\;\;+\frac{1}{2}{\left\|{\text{A}}_{1}{\text{x}}_{1}^{\left(k+1\right)}+{\text{A}}_{\text{2}}{\text{x}}_{\text{2}}+{\text{A}}_{3}{\text{x}}_{3}+{\text{A}}_{4}{\text{x}}_{4}-\text{b}\right\|}_{2}^{2}\}\\ \lambda^{\left(k+1\right)}=\lambda^{\left(k\right)}-({\text{A}}_{1}{\text{x}}_{1}^{\left(k+1\right)}+{\text{A}}_{2}{\text{x}}_{2}^{\left(k+1\right)}+{\text{A}}_{3}{\text{x}}_{3}^{\left(k+1\right)}+{\text{A}}_{4}{\text{x}}_{4}^{\left(k+1\right)}-\text{b})\;. \end{array}$$

Clearly, (5) is a specific application of the original two-block ADMM to (3) by regarding (**x**_2_, **x**_3_, **x**_4_) as one variable, [**A**_2_, **A**_3_, **A**_4_] as one matrix and *θ*_2_(**x**_2_) + *θ*_3_(**x**_3_) + *θ*_4_(**x**_4_) as one function. ■

## Appendix C. Gecco+ for Differentiable Losses

In this section, we propose algorithms to solve Gecco+ when the loss ***ℓ*** is differentiable and gradient is Lipschitz continuous. In this case, we develop a fast two-block ADMM algorithm without fully solving the **U** sub-problem. Our result is closely related to the proximal linearized ADMM literature (Liu et al., 2013; Lu et al., 2016). Also solving the sub-problem approximately is closely connected with the generalized ADMM literature (Deng and Yin, 2016).

In the following sections, we discuss algorithms to solve Gecco+ instead of iGecco+ for notation purposes as we would like to include iteration counter indices in the algorithm for illustrating backtracking; but we can easily extend the algorithm to solve iGecco+. To begin with, we clarify different notations in Gecco+ and iGecco+: the superscript in **U**^(*k*)^ in iGecco+ refers to the *k^th^* data view while **U**^(*k*)^ in Gecco+ refers to the *k^th^* iteration counter in the ADMM updates. We omit iteration counter indices in all iGecco+ algorithm for notation purposes and use the most recent values of the updates.

### C.1 Two-block ADMM in Matrix Form

Suppose the loss ℓ(X,U) is differentiable. Similar to the formulation in convex clustering, we can recast the Gecco+ problem as the equivalent constrained problem:

$$\begin{array}{l} \underset{U,V}{\mathrm{minimize}}\;\ell \left(X,U\right)+\gamma \underset{P_{1}\left(V;w\right)}{\underbrace{\left(\sum_{l\in \varepsilon }w_{l}{\left\|V_{l.}\right\|}_{2}\right)}}+\alpha \sum_{j=1}^{p}\zeta_{j}{\left\|U_{.j}-{\tilde{x}}_{j}\cdot 1_{n}\right\|}_{2}\\ \text{subject to}\;\mathrm{DU}-V=0\;. \end{array}$$

Like in convex clustering (Chi and Lange, 2015; Weylandt et al., 2020), we index a centroid pair by $l=\left(l_{1},l_{2}\right)$ with $l_{1}<l_{2}$, define the set of edges over the non-zero weights $\varepsilon =\left\{l=\left(l_{1},l_{2}\right):w_{l}>0\right\}$, and introduce a new variable ${\text{V}}_{l.}={\text{U}}_{l_{1.}}-{\text{U}}_{l_{2.}}$ to account for the difference between the two centroids. Hence **V** is a matrix containing the pairwise differences between connected rows of **U**. Also **D** is the directed difference matrix corresponding to the non-zero fusion weights defined in the work of Weylandt et al. (2020).

We can show that the augmented Lagrangian in scaled form is equal to:

$$L\left(U,V,\Lambda \right)=\ell \left(X,U\right)+\frac{\rho }{2}{\left\|\mathrm{DU}-V+\Lambda \right\|}_{F}^{2}+\alpha \sum_{j=1}^{p}\zeta_{j}{\left\|{\text{U}}_{.j}-{\tilde{x}}_{j}\cdot 1_{n}\right\|}_{2}+\gamma \sum_{l\in \varepsilon }w_{l}{\left\|{\text{V}}_{l.}\right\|}_{2}\;,$$

where the dual variable is denoted by **Λ**.

To update **U**, we need to solve the following sub-problem:

$$\underset{U}{\mathrm{minimize}}\;\ell \left(X,U\right)+\frac{\rho }{2}{\left\|\mathrm{DU}-V+\Lambda \right\|}_{2}^{2}+\alpha \sum_{j=1}^{p}\zeta_{j}{\left\|{\text{U}}_{.j}-{\tilde{x}}_{j}\cdot 1_{n}\right\|}_{2}\;.$$

Let $\tilde{U}=U-\tilde{X}$. The sub-problem becomes:

$$\underset{U}{\mathrm{minimize}}\;\ell \left(\text{X},\tilde{U}+\tilde{X}\right)+\frac{\rho }{2}{\left\|\text{D}\left(\tilde{U}+\tilde{X}\right)-\text{V}+\Lambda \right\|}_{2}^{2}+\alpha \sum_{j=1}^{p}\zeta_{j}{\left\|{\tilde{u}}_{j}\right\|}_{2}\;,$$

where ${\tilde{u}}_{j}$ is the *j^th^* column of $\tilde{U}$. For each ADMM iterate, we have:

$${\tilde{U}}^{\left(k\right)}=\underset{\tilde{U}}{\mathrm{argmin}}\;\ell \left(X,\tilde{U}+\tilde{X}\right)+\frac{\rho }{2}{\left\|\text{D}\left(\tilde{U}+\tilde{X}\right){-V}^{\left(k-1\right)}+\Lambda^{\left(k-1\right)}\right\|}_{2}^{2}+\alpha \underset{P_{2}\left(\tilde{U};\zeta \right)}{\underbrace{\sum_{j=1}^{p}\zeta_{j}{\left\|{\tilde{u}}_{j}\right\|}_{2}}}\;.$$

This can be solved by running iterative proximal gradient to full convergence:

$${\tilde{U}}^{\left(k,m\right)}=\underset{s_{k}\cdot \alpha P_{2}\left(\cdot ;\zeta \right)}{\text{prox}}\;\left({\tilde{U}}^{\left(k,m-1\right)}-s_{k}\cdot \left[\nabla \ell \left({X,\tilde{U}}^{\left(k,m-1\right)}+\tilde{X}\right){+}_{\rho }{\text{D}}^{T}\left(\text{D}\left({\tilde{U}}^{\left(k,m-1\right)}+\tilde{X}\right)-{\text{V}}^{\left(k-1\right)}+\Lambda^{\left(k-1\right)}\right)\right]\right)\;,$$

which is equivalent to:

$${\text{U}}^{\left(k,m\right)}=\underset{s_{k}\cdot \alpha P_{2}\left(\cdot ;\zeta \right)}{\text{prox}}\;\left({\text{U}}^{\left(k,m-1\right)}-\tilde{X}-s_{k}\cdot \left[\nabla \ell \left({X,U}^{\left(k,m-1\right)}\right){+}_{\rho }{\text{D}}^{T}\left({\text{DU}}^{\left(k,m-1\right)}-{\text{V}}^{\left(k-1\right)}+\Lambda^{\left(k-1\right)}\right)\right]\right)+\tilde{X}\;.$$

Here **U**^(*k,m*)^ refers to the *m^th^* inner iteration counter in the **U** sub-problem out of the *k^th^* outer iteration counter of the ADMM update. It is straightforward that this is computationally expensive. To address this, we propose to solve the **U** sub-problem approximately using just a one-step proximal gradient update and prove convergence in the next section. This approach is based on proximal linearized ADMM (Liu et al., 2013; Lu et al., 2016), which solves the sub-problems efficiently by linearizing the differentiable part and then applying proximal gradient due to the non-differentiable part. To ensure convergence, the algorithm requires that gradient should be Lipschitz continuous. The **V** and **Λ** updates are just the same as in regular convex clustering.

We adopt such an approach and develop the proximal linearized 2-block ADMM (Algorithm 7) to solve Gecco+ when the loss is differentiable and gradient is Lipschitz continuous.

**Algorithm 7:** Proximal linearized 2-block ADMM when the loss is differentiable and gradient is Lipschitz continuous — matrix form

**while** not converged **do**
${\text{U}}^{\left(k\right)}={\text{prox}}_{s_{k}\cdot \alpha P_{2}\left(\cdot ;\zeta \right)}\left({\text{U}}^{\left(k-1\right)}-\tilde{X}-s_{k}\cdot \left[\nabla \ell \left({\text{X},\text{U}}^{\left(k-1\right)}\right){+}_{\rho }{\text{D}}^{T}\left({\text{DU}}^{\left(k-1\right)}-{\text{V}}^{\left(k-1\right)}+\Lambda^{\left(k-1\right)}\right)\right]\right)+\tilde{X}$
${\text{V}}^{\left(k\right)}={\text{prox}}_{\gamma /\rho P_{1}\left(\cdot ;\text{w}\right)}\left({\text{DU}}^{\left(k\right)}+\Lambda^{\left(k-1\right)}\right)$
$\Lambda^{\left(k\right)}=\Lambda^{\left(k-1\right)}+\left({\text{DU}}^{\left(k\right)}-{\text{V}}^{\left(k\right)}\right)$
**end while**

Further, if the **U** sub-problem can be decomposed to *p* separate **U**_.*j*_ sub-problems where the augmented Lagrangian for each now is a sum of a differentiable loss, a quadratic term and a sparse group-lasso penalty, we propose to use proximal gradient descent for each separate **U**_.*j*_ sub-problem. In this way, we yield adaptive step size for each **U**_.*j*_ sub-problem and hence our algorithm enjoys better convergence property than updating **U**’s all together. (In the latter case, the step size becomes fairly small as we are moving all **U** to some magnitude in the direction of negative gradient.) To achieve this, we assume that the loss is elementwise, which means we can write the loss function as a sum of *p* terms. (The loss can be written as $\sum_{i}\ell \left({\text{X}}_{i.},{\text{U}}_{i.}\right)=\sum_{j}\ell \left({\text{X}}_{.j},{\text{U}}_{.j}\right)=\sum_{i}\sum_{j}q\left(x_{ij},u_{ij}\right)$ where *q*(·) is the element-wise version of the loss while *ℓ*(·) is the vector-wise version of the loss.) We see that every deviance-based loss satisfies this assumption. Moreover, by decomposing to *p* sub-problems, we can solve each in parallel which saves computation cost. We describe in detail how to solve each **U**_.*j*_ sub-problem in the next subsection.

### C.2 Two-block ADMM in Vector Form in Parallel

Suppose the **U** sub-problem can be decomposed to *p* separate **U**_.*j*_ sub-problems mentioned above. The augmented Lagrangian now becomes:

$$\begin{array}{l} L\left(U,V,\Lambda \right)=\sum_{j=1}^{p}\ell \left({\text{X}}_{.j},{\text{U}}_{.j}\right)+\frac{\rho }{2}\sum_{j=1}^{p}{\left\|{\text{DU}}_{.j}-{\text{V}}_{.j}+\Lambda_{.j}\right\|}_{2}^{2}\\ \;\;\;\;+\alpha \sum_{j=1}^{p}\zeta_{j}{\left\|{\text{U}}_{.j}-{\tilde{x}}_{j}\cdot 1_{n}\right\|}_{2}+\gamma \sum_{l\in \varepsilon }w_{l}{\left\|{\text{V}}_{l.}\right\|}_{2}\;. \end{array}$$

In this way we can perform block-wise minimization. Now minimizing the augmented Lagrangian over **U** is equivalent to minimizing over each ${\text{U}}_{.j},j=1,\cdots ,p$:

$$\underset{{\text{U}}_{.j}}{\mathrm{minimize}}\;\ell \left({\text{X}}_{.j},{\text{U}}_{.j}\right)+\frac{\rho }{2}{\left\|{\text{DU}}_{.j}-{\text{V}}_{.j}+\Lambda_{.j}\right\|}_{2}^{2}+\alpha \zeta_{j}{\left\|{\text{U}}_{.j}-{\tilde{x}}_{j}\cdot 1_{n}\right\|}_{2}.$$

Let ${\tilde{u}}_{j}={\text{U}}_{.j}-{\tilde{x}}_{j}\cdot 1_{n}$. The problem above becomes:

$$\underset{{\tilde{u}}_{j}}{\mathrm{minimize}}\;\ell \left({\text{X}}_{.j},{\tilde{u}}_{j}+{\tilde{x}}_{j}\cdot 1_{n}\right)+\frac{\rho }{2}{\left\|\text{D}\left({\tilde{u}}_{j}+{\tilde{x}}_{j}\cdot 1_{n}\right)-{\text{V}}_{.j}+\Lambda_{.j}\right\|}_{2}^{2}+\alpha \zeta_{j}{\left\|{\tilde{u}}_{j}\right\|}_{2}\;.$$

Similarly, this can be solved by running iterative proximal gradient to full convergence. However, as mentioned above, we propose to solve the **U** sub-problem approximately by taking just a one-step proximal gradient update and prove convergence. Still this approach is based on proximal linearized ADMM (Liu et al., 2013; Lu et al., 2016), which solves the sub-problems efficiently by linearizing the differentiable part and then applying proximal gradient due to the non-differentiable part. To ensure convergence, the algorithm requires that gradient should be Lipschitz continuous.

We propose Algorithm 8 to solve Gecco+ when *ℓ* is differentiable and gradient is Lipschitz continuous in vector form. Note the **U**-update in Algorithm 8 is a just a vectorized version of that in Algorithm 7 if we use fixed step size *s_k_* for each feature *j*. We use the vector form update here since it enjoys better convergence property mentioned above and we use this form to prove convergence. Next we prove the convergence of Algorithm 8.

**Algorithm 8:** Proximal linearized 2-block ADMM when the loss is differentiable and gradient is Lipschitz continuous — vector form in parallel

**Input:** X,γ,w,α,ζ
**Initialize:** ${\text{U}}^{\left(0\right)},{\text{V}}^{\left(0\right)},\Lambda^{\left(0\right)}$
**Precompute:** Difference matrix ${\text{D},\tilde{x}}_{j}$
**while** not converged **do**
**for** *j* = 1 **to** *p* **do**
$\begin{array}{l} {\text{U}}_{.j}^{\left(k\right)}={\text{prox}}_{s_{k}\cdot \alpha \zeta_{j}{\left\|\cdot \right\|}_{2}}({\text{U}}_{.j}^{\left(k-1\right)}-{\tilde{x}}_{j}\cdot 1_{n}-s_{k}\cdot [\nabla \ell \left({\text{X}}_{.j},{\text{U}}_{.j}^{\left(k-1\right)}\right)+\rho {\text{D}}^{T}({\text{DU}}_{.j}^{\left(k-1\right)}-{\text{V}}_{.j}^{\left(k-1\right)}+\\ \;\;\;\Lambda_{.j}^{\left(k-1\right)})])+{\tilde{x}}_{j}\cdot 1_{n} \end{array}$
**end for**
${\text{V}}^{\left(k\right)}={\text{prox}}_{\gamma /\rho P_{1}\left(\cdot ;\text{w}\right)}\left({\text{DU}}^{\left(k\right)}+\Lambda^{\left(k-1\right)}\right)$
$\Lambda^{\left(k\right)}=\Lambda^{\left(k-1\right)}+\left({\text{DU}}^{\left(k\right)}-{\text{V}}^{\left(k\right)}\right)$
**end while**
**Output: U**^(*k*)^.

### C.3 Proof of Convergence

**Theorem 6** *If ℓ is convex and differrentiable and ∇ℓ is Lipschitz continuous, then* Algorithm 8 *converges to a global solution. Further, if ℓ is strictly convex, it converges to the unique global solution*.

**Proof:** We will show that the **U**-update in Algorithm 8 is equivalent to linearizing the **U** sub-problem and then applying a proximal operator, which is proximal linearized ADMM.

Note that each **U**_.*j*_ sub-problem is:

$${\text{U}}_{.j}^{\left(k+1\right)}=\underset{{\text{U}}_{.j}}{\mathrm{argmin}}\;\ell \left({\text{X}}_{.j},{\text{U}}_{.j}\right)+\frac{\rho }{2}{\left\|{\text{DU}}_{.j}-{\text{V}}_{.j}^{\left(k\right)}+\Lambda_{.j}^{\left(k\right)}\right\|}_{2}^{2}+\alpha \zeta_{j}{\left\|{\text{U}}_{.j}-{\tilde{x}}_{j}\cdot 1_{n}\right\|}_{2}\;.$$

For simplicity of notation, we replace **U**_.*j*_ with **u**_*j*_ in the following:

$${\text{u}}_{j}^{\left(k+1\right)}=\underset{{\text{u}}_{j}}{\mathrm{argmin}}\;\ell \left({\text{X}}_{.j},{\text{u}}_{j}\right)+\frac{\rho }{2}{\left\|\text{D}{\text{u}}_{j}-{\text{V}}_{.j}^{\left(k\right)}+\Lambda_{.j}^{\left(k\right)}\right\|}_{2}^{2}+\alpha \zeta_{j}{\left\|{\text{u}}_{j}-{\tilde{x}}_{j}\cdot 1_{n}\right\|}_{2}\;.$$

Rearranging terms, we have:

$${\text{u}}_{j}^{\left(k+1\right)}=\underset{{\text{u}}_{j}}{\mathrm{argmin}}\;\ell \left({\text{X}}_{.j},{\text{u}}_{j}\right)+\frac{\rho }{2}{\left\|\text{D}{\text{u}}_{j}-{\text{V}}_{.j}^{\left(k\right)}\right\|}_{2}^{2}+\rho \Lambda_{.j}^{{\left(k\right)}^{T}}\left(\text{D}{\text{u}}_{j}-{\text{V}}_{.j}^{\left(k\right)}\right)+\alpha \zeta_{j}{\left\|{\text{u}}_{j}-{\tilde{x}}_{j}\cdot 1_{n}\right\|}_{2}\;.$$

According to the proximal linearized ADMM with parallel splitting algorithm (see Algorithm 3 of Liu et al. (2013) and Equation (14) of Lu et al. (2016)), we can linearize the first two terms and add a quadratic term in the objective:

$$\begin{array}{l} {\text{u}}_{j}^{\left(k+1\right)}=\underset{{\text{u}}_{j}}{\mathrm{argmin}}\;\ell \left({\text{X}}_{.j},{\text{u}}_{j}^{\left(k\right)}\right)+\langle \nabla \ell \left({\text{X}}_{.j},{\text{u}}_{j}^{\left(k\right)}\right),{\text{u}}_{j}-{\text{u}}_{j}^{\left(k\right)}\rangle \\ \;\;\;\;\;\;\;\;+\langle \rho {\text{D}}^{T}\left(\text{D}{\text{u}}_{j}^{\left(k\right)}-{\text{V}}_{.j}^{\left(k\right)}\right),{\text{u}}_{j}-{\text{u}}_{j}^{\left(k\right)}\rangle \\ \;\;\;\;\;\;\;\;+\rho \Lambda_{.j}^{{\left(k\right)}^{T}}\left(\text{D}{\text{u}}_{j}-{\text{V}}_{.j}^{\left(k\right)}\right)+\alpha \zeta_{j}{\left\|{\text{u}}_{j}-{\tilde{x}}_{j}\cdot 1_{n}\right\|}_{2}+\frac{1}{2s_{k}}{\left\|{\text{u}}_{j}-{\text{u}}_{j}^{\left(k\right)}\right\|}_{2}^{2}\;. \end{array}$$

Rearranging terms and removing irrelevant terms, we have:

$${\text{u}}_{j}^{\left(k+1\right)}=\underset{{\text{u}}_{j}}{\mathrm{argmin}}\;{\left(\nabla g\left({\text{u}}_{j}^{\left(k\right)}\right)\right)}^{T}\left({\text{u}}_{j}-{\text{u}}_{j}^{\left(k\right)}\right)+\frac{1}{2s_{k}}{\left\|{\text{u}}_{j}-{\text{u}}_{j}^{\left(k\right)}\right\|}_{2}^{2}+\alpha \zeta_{j}{\left\|{\text{u}}_{j}-{\tilde{x}}_{j}\cdot 1_{n}\right\|}_{2}\;,$$

where $\nabla g\left({\text{u}}_{j}^{\left(k\right)}\right)=\nabla \ell \left({\text{X}}_{.j},{\text{u}}_{j}^{\left(k\right)}\right)+\rho {\text{D}}^{T}\left(\text{D}{\text{u}}_{j}^{\left(k\right)}-{\text{V}}_{.j}^{\left(k\right)}+\Lambda_{.j}^{\left(k\right)}\right))$.

Let ${\hat{u}}_{j}={\text{u}}_{j}-{\tilde{x}}_{j}\cdot 1_{n}$. We have:

$${\hat{u}}_{j}^{\left(k+1\right)}=\underset{{\hat{u}}_{j}}{\mathrm{argmin}}\;{\left(\nabla g\left({\text{u}}_{j}^{\left(k\right)}\right)\right)}^{T}\left({\hat{u}}_{j}+{\tilde{x}}_{j}\cdot 1_{n}-{\text{u}}_{j}^{\left(k\right)}\right)+\frac{1}{2s_{k}}{\left\|{\hat{u}}_{j}{+\tilde{\text{x}}}_{j}\cdot 1_{n}-{\text{u}}_{j}^{\left(k\right)}\right\|}_{2}^{2}+\alpha \zeta_{j}{\left\|{\hat{u}}_{j}\right\|}_{2}\;.$$

Recall the definition of proximal operator:

$$\begin{array}{l} {\text{x}}^{\left(k+1\right)}=\underset{th}{\text{prox}}\left({\text{x}}^{\left(k\right)}-t\nabla g\left({\text{x}}^{\left(k\right)}\right)\right)\\ \;\;\;=\underset{\text{u}}{\mathrm{argmin}}\left(h\left(\text{u}\right)+g\left({\text{x}}^{\left(k\right)}\right)+\nabla g{\left({\text{x}}^{\left(k\right)}\right)}^{T}\left(\text{u}-{\text{x}}^{\left(k\right)}\right)+\frac{1}{2t}{\left\|\text{u}-{\text{x}}^{\left(k\right)}\right\|}_{2}^{2}\right)\;. \end{array}$$

Therefore, the ${\hat{u}}_{j}$ update is just a proximal gradient descent update:

$${\hat{u}}_{j}^{\left(k+1\right)}=\underset{s_{k}\cdot \alpha \zeta_{j}{\left\|\cdot \right\|}_{2}}{\text{prox}}\;\left({\text{u}}_{j}^{\left(k\right)}-{\tilde{x}}_{j}\cdot 1_{n}-s_{k}\cdot \left[\nabla \ell \left({\text{X}}_{.j},{\text{u}}_{j}^{\left(k\right)}\right)+\rho {\text{D}}^{T}\left(\text{D}{\text{u}}_{j}^{\left(k\right)}-{\text{V}}_{.j}^{\left(k\right)}+\Lambda_{.j}^{\left(k\right)}\right)\right]\right)\;.$$

Now we plug back and get the ${\text{u}}_{j}$, i.e., ${\text{U}}_{.j}$ update:

$$\begin{array}{l} {\text{U}}_{.j}^{\left(k\right)}=\underset{s_{k}\cdot \alpha \zeta_{j}{\left\|\cdot \right\|}_{2}}{\text{prox}}\;\left({\text{U}}_{.j}^{\left(k-1\right)}-{\tilde{x}}_{j}\cdot 1_{n}-s_{k}\cdot \left[\nabla \ell \left({\text{X}}_{.j},{\text{U}}_{.j}^{\left(k-1\right)}\right)+\rho {\text{D}}^{T}\left(\text{D}{\text{U}}_{.j}^{\left(k-1\right)}-{\text{V}}_{.j}^{\left(k-1\right)}+\Lambda_{.j}^{\left(k-1\right)}\right)\right]\right)\\ \;\;\;\;\;\;\;\;+{\tilde{x}}_{j}\cdot 1_{n}\;, \end{array}$$

which is equivalent to the **U**_.*j*_ update in Algorithm 8.

The **V** and **Λ** update is just the same as the one in convex clustering. Hence Algorithm 8 is equivalent to the form of proximal linearized ADMM by Liu et al. (2013); Lu et al. (2016) and hence converges to a global solution as long as ∇*ℓ* is Lipschitz continuous. Note that Algorithm 7 is equivalent to Algorithm 8 with a fixed step size *s_k_*. (We can choose *s_k_* to be the minimum step size *s_k_* over all features.) Therefore, Algorithm 7 also converges to a global solution as long as ∇*ℓ* is Lipschitz continuous. Further, if *ℓ* is strictly convex, the optimization problem has unique minimum and hence Algorithm 7 and 8 converges to the global solution. ■

Note:

- In Liu et al. (2013); Lu et al. (2016), the algorithm requires that ∇*ℓ* is Lipschitz continuous to guarantee convergence. We know that ∇*ℓ* being Lipschitz continuous is equivalent to *ℓ* being strongly smooth. It is easy to show that the Hessian of log-likelihood of exponential family and GLM deviance is upper bounded since in (generalized) convex clustering, the value of **U** is bounded as it moves along the regularization path from **X** to the loss-specific center; also to avoid numerical issues, we add trivial constraint that *u_ij_* > 0 when zero is not defined in the log-likelihood/deviance. Hence the condition for convergence of proximal linearized ADMM is satisfied.
- To obtain a reasonable step size *s_k_*, we need to compute the Lipschitz constant. However, it is non-trivial to calculate the Lipschitz constant for most of our general losses. Instead, we suggest using backtracking line search procedure proposed by Beck and Teboulle (2009); Parikh et al. (2014), which is a common way to determine step size with guaranteed convergence in optimization. Empirical studies show that choosing step size with backtracking in our framework also ensures convergence. The details for backtracking procedure are discussed below.
- For proximal linearized ADMM, Liu et al. (2013); Lu et al. (2016) established convergence rate of *O*(1/*K*). An interesting future direction might be establishing the linear convergence rate of proximal linearized ADMM when the objective is strongly convex.

### C.4 Backtracking Criterion

In this section we discuss how to choose the step size *s_k_* in Algorithm 8. As mentioned, usually we employ a fixed step size by computing the Lipschitz constant as in the squared error loss case; but in our method, it is hard to compute the Lipschitz constant for most of our general losses. Instead, we propose using backtracking line search procedure proposed by Beck and Teboulle (2009); Parikh et al. (2014), which is a common way to determine step size with guaranteed convergence in optimization.

Recall the objective function we want to minimize in the **U** sub-problem is:

$$f\left({\tilde{u}}_{j}\right)=\ell \left({\text{X}}_{.j},{\tilde{u}}_{j}+{\tilde{x}}_{j}\cdot 1_{n}\right)+\frac{\rho }{2}{\left\|\text{D}\left({\tilde{u}}_{j}+{\tilde{x}}_{j}\cdot 1_{n}\right)-{\text{V}}_{.j}+\Lambda_{.j}\right\|}_{2}^{2}+\alpha \zeta_{j}{\left\|{\tilde{u}}_{j}\right\|}_{2}\;,$$

where ${\tilde{u}}_{j}={\text{U}}_{.j}-{\tilde{x}}_{j}\cdot 1_{n}$.

Define:

$$\begin{array}{l} g\left({\tilde{u}}_{j}\right)=\ell \left({\text{X}}_{.j},{\tilde{u}}_{j}+{\tilde{x}}_{j}\cdot 1_{n}\right)+\frac{\rho }{2}{\left\|\text{D}\left({\tilde{u}}_{j}+{\tilde{x}}_{j}\cdot 1_{n}\right)-{\text{V}}_{.j}+\Lambda_{.j}\right\|}_{2}^{2}\\ h\left({\tilde{u}}_{j}\right)=\alpha \zeta_{j}{\left\|{\tilde{u}}_{j}\right\|}_{2}\\ G_{t}\left({\tilde{u}}_{j}\right)=\frac{{\tilde{u}}_{j}-{\text{prox}}_{t\cdot \alpha \zeta_{j}{\left\|\cdot \right\|}_{2}}\left({\tilde{u}}_{j}-t\nabla g\left({\tilde{u}}_{j}\right)\right)}{t}. \end{array}$$

We adopt the backtracking line search procedure proposed by Beck and Teboulle (2009); Parikh et al. (2014). At each iteration, while

$$\begin{array}{l} \;\;\;g\left({\tilde{u}}_{j}-tG_{t}\left({\tilde{u}}_{j}\right)\right)>g\left({\tilde{u}}_{j}\right)-t\nabla g{\left({\tilde{u}}_{j}\right)}^{T}G_{t}\left({\tilde{u}}_{j}\right)+\frac{t}{2}{\left\|G_{t}\left({\tilde{u}}_{j}\right)\right\|}_{2}^{2}\;\;\mathrm{i.e.},\\ g\left(\underset{t}{\text{prox}}\left({\tilde{u}}_{j}-t\nabla g\left({\tilde{u}}_{j}\right)\right)\right)>g\left({\tilde{u}}_{j}\right)-\nabla g{\left({\tilde{u}}_{j}\right)}^{T}\left({\tilde{u}}_{j}-\underset{t}{\text{prox}}\left({\tilde{u}}_{j}-t\nabla g\left({\tilde{u}}_{j}\right)\right)\right)\\ \;\;\;\;\;\;\;\;\;+\frac{1}{2t}{\left\|{\tilde{u}}_{j}-\underset{t}{\text{prox}}\left({\tilde{u}}_{j}-t\nabla g\left({\tilde{u}}_{j}\right)\right)\right\|}_{2}^{2} \end{array}$$

shrink *t* = *βt*.

We still adopt the one-step approximation and hence suggest taking a one-step proximal update with backtracking to solve the **U** sub-problem. To summarize, we propose Algorithm 9, which uses proximal linearized 2-block ADMM with backtracking when the loss is differentiable and gradient is Lipschitz continuous.

### C.5 Alternative Algorithm for Differentiable Losses

It should be pointed out that there are many other methods to solve the **U** sub-problem when the loss *ℓ* is differentiable. We choose to use proximal gradient descent algorithm as there is existing literature on approximately solving the sub-problem using proximal gradient under ADMM with proved convergence (Liu et al., 2013; Lu et al., 2016). But there are many other optimization techniques to solve the **U** sub-problem such as ADMM.

In this subsection, we show how to apply ADMM to solve the **U** sub-problem and specify under which conditions this method is more favorable. Recall to update **U**, we need to solve the following sub-problem:

$$\underset{\text{U}}{\mathrm{minimize}}\;\ell \left(X,U\right)+\frac{\rho }{2}{\left\|\mathrm{DU}-V+\Lambda \right\|}_{F}^{2}+\alpha \sum_{j=1}^{p}\zeta_{j}{\left\|{\text{U}}_{.j}-{\tilde{x}}_{j}\cdot 1_{n}\right\|}_{2}\;.$$

We use ADMM to solve this minimization problem and can now recast the problem above as the equivalent constrained problem:

$$\begin{array}{l} \underset{U,V,\Lambda ,R}{\mathrm{minimize}}\;\sum_{j=1}^{p}\ell \left({\text{X}}_{.j},{\text{U}}_{.j}\right)+\frac{\rho }{2}{\left\|\mathrm{DU}-V+\Lambda \right\|}_{F}^{2}+\underset{P_{2}\left(R;\zeta \right)}{\underbrace{\alpha (\sum_{j=1}^{p}\zeta_{j}{\left\|{\text{r}}_{j}\right\|}_{2})}}\\ \text{subject to}\;\text{U}-\tilde{X}=R\;. \end{array}$$

**Algorithm 9:** Proximal linearized 2-block ADMM with backtracking when the loss is differentiable and gradient is Lipschitz continuous

**Input:** X,γ,w,α,ζ
**Initialize: U**^(0)^, **V**^(0)^, **Λ**^(0)^, *t*
**Precompute:** Difference matrix **D**, ${\tilde{x}}_{j}$
**while** not converged **do**
**for** *j* = 1 **to** *p* **do**
*t* = 1
${\tilde{u}}_{j}^{\left(k-1\right)}={\text{U}}_{.j}^{\left(k-1\right)}-{\tilde{x}}_{j}\cdot 1_{n}$
$\nabla g\left({\tilde{u}}_{j}^{\left(k-1\right)}\right)=\nabla \ell \left({\text{X}}_{.j},{\tilde{u}}_{j}^{\left(k-1\right)}+{\tilde{x}}_{j}\cdot 1_{n}\right)+\rho {\text{D}}^{T}\left(\text{D}\left({\tilde{u}}_{j}^{\left(k-1\right)}+{\tilde{x}}_{j}\cdot 1_{n}\right)-{\text{V}}_{.j}^{\left(k-1\right)}+\Lambda_{.j}^{\left(k-1\right)}\right)$
$z={\text{prox}}_{t\alpha \zeta_{j}{\left\|\cdot \right\|}_{2}}\left({\tilde{u}}_{j}^{\left(k-1\right)}-t\nabla g\left({\tilde{u}}_{j}^{\left(k-1\right)}\right)\right)$
**while** $g\left(z\right)>g({\tilde{u}}_{j}^{\left(k-1\right)}-\nabla g{\left({\tilde{u}}_{j}^{\left(k-1\right)}\right)}^{T}\left({\tilde{u}}_{j}^{\left(k-1\right)}-z\right)+\frac{1}{2t}{\left\|z-{\tilde{u}}_{j}^{\left(k-1\right)}\right\|}_{2}^{2}\mathrm{do}$
*t* = *βt*
$z={\text{prox}}_{t\alpha \zeta_{j}{\left\|\cdot \right\|}_{2}}\left({\tilde{u}}_{j}^{\left(k-1\right)}-t\nabla g\left({\tilde{u}}_{j}^{\left(k-1\right)}\right)\right)$
**end while**
${\text{U}}_{.j}^{\left(k\right)}=z+{\tilde{x}}_{j}\cdot 1_{n}$
**end for**
${\text{V}}^{\left(k\right)}={\text{prox}}_{\gamma /\rho P_{1}\left(\cdot ;\text{w}\right)}\left({\text{DU}}^{\left(k\right)}+\Lambda^{\left(k-1\right)}\right)$
$\Lambda^{\left(k\right)}=\Lambda^{\left(k-1\right)}+\left({\text{DU}}^{\left(k\right)}-{\text{V}}^{\left(k\right)}\right)$
**end while**
**Output: U**^(*k*)^.

The augmented Lagrangian in scaled form is:

$$\begin{array}{l} L\left(U,V,R,\Lambda ,N\right)=\sum_{j=1}^{p}\ell \left({\text{X}}_{.j},{\text{U}}_{.j}\right)+\frac{\rho }{2}{\left\|\mathrm{DU}-V+\Lambda \right\|}_{F}^{2}+\frac{\rho }{2}{\left\|\left(U-\tilde{X}\right)-R+N\right\|}_{F}^{2}\\ \;\;\;\;\;\;\;+\alpha \sum_{j=1}^{p}\zeta_{j}{\left\|{\text{r}}_{j}\right\|}_{2}\;, \end{array}$$

where the dual variable for **V** is denoted by **Λ**; the dual variable for **R** is denoted by **N**.

The **U**_.*j*_ sub-problem in the inner nested ADMM is:

$$\begin{array}{l} {\text{U}}_{.j}^{\left(k\right)}=\underset{{\text{U}}_{.j}}{\mathrm{argmin}}\;\ell \left({\text{X}}_{.j},{\text{U}}_{.j}\right)+\frac{\rho }{2}{\left\|{\text{DU}}_{.j}-{\text{V}}_{.j}^{\left(k-1\right)}+\Lambda_{.j}^{\left(k-1\right)}\right\|}_{F}^{2}\\ \;\;\;\;\;\;\;\;\;\;\;+\frac{\rho }{2}{\left\|\left({\text{U}}_{.j}-{\tilde{x}}_{j}\cdot 1_{n}\right)-{\text{r}}_{j}^{\left(k-1\right)}+{\text{N}}_{.j}^{\left(k-1\right)}\right\|}_{F}^{2}. \end{array}$$

Now, we are minimizing a sum of a differentiable loss *ℓ* and two quadratic terms which are all smooth. Still the **U**_.*j*_ sub-problem does not have a closed-form solution for general convex losses and we need to run an iterative descent algorithm (such as gradient descent, Newton method) to full convergence to solve the problem. Similarly, to reduce computation cost, we take a one-step update by applying linearized ADMM (Lin et al., 2011) to the **U**_.*j*_

**Algorithm 10:** Full-step multi-block algorithm for Gecco+ with Bernoulli log-likelihood *ℓ_k_*:

**Precompute:** Difference matrix $D,M_{1}={\left(\frac{1}{4}\text{I}+\rho {\text{D}}^{T}\text{D}+\rho \text{I}\right)}^{-1}$.
**while** not converged **do**
**while** not converged **do**
${\text{U}}^{\left(k\right)}={\text{U}}^{\left(k-1\right)}-{\text{M}}_{1}\left[\nabla \ell \left({\text{X},\text{U}}^{\left(k-1\right)}\right)+\rho {\text{D}}^{T}\left({\text{DU}}^{\left(k-1\right)}-{\text{V}}^{\left(k-1\right)}+\Lambda^{\left(k-1\right)}\right)+\rho ({\text{U}}^{\left(k-1\right)}-\tilde{X}-R^{\left(k-1\right)}+{\text{N}}^{\left(k-1\right)})\right]$
${\text{R}}^{\left(k\right)}={\text{prox}}_{\alpha /\rho P_{2}\left(\cdot ;\zeta \right)}\left({\text{U}}^{\left(k\right)}-\tilde{X}+N^{\left(k-1\right)}\right)$
${\text{N}}^{\left(k\right)}=N^{\left(k-1\right)}+\left({\text{U}}^{\left(k\right)}-\tilde{X}-R^{\left(k\right)}\right)$
**end while**
${\text{V}}^{\left(k\right)}={\text{prox}}_{\gamma /\rho P_{1}\left(\cdot ;\text{w}\right)}\left({\text{DU}}^{\left(k\right)}+\Lambda^{\left(k-1\right)}\right)$
$\Lambda^{\left(k\right)}=\Lambda^{\left(k-1\right)}+\left({\text{DU}}^{\left(k\right)}-{\text{V}}^{\left(k\right)}\right)$
**end while**

sub-problem. The **U**_.*j*_ update in the inner ADMM now becomes:

$$\begin{array}{l} {\text{U}}_{.j}^{\left(k\right)}={\text{U}}_{.j}^{\left(k-1\right)}-s_{k}[\nabla \ell \left({\text{X}}_{.j},{\text{U}}_{.j}^{\left(k-1\right)}\right)+\rho {\text{D}}^{T}\left({\text{DU}}_{.j}^{\left(k-1\right)}-{\text{V}}_{.j}^{\left(k-1\right)}+\Lambda_{.j}^{\left(k-1\right)}\right)\\ \;\;\;\;\;\;\;\;\;\;\;\;\;\;\;+\rho \left({\text{U}}_{.j}^{\left(k-1\right)}-{\tilde{x}}_{j}\cdot 1_{n}-{\text{r}}_{j}^{\left(k-1\right)}+{\text{N}}_{.j}^{\left(k-1\right)}\right)]\;. \end{array}$$

In this case, empirical studies show that taking a one-step Newton update is favored than a one-step gradient descent update as the former enjoys better convergence properties and generally avoids backtracking. However, inverting a Hessian matrix is computationally burdensome at each iteration when *n* is large. Exceptions are for Euclidean distances case where there is a closed-form solution for the **U**_.*j*_ update and for Bernoulli log-likelihood case where the Hessian of the loss can be upper bounded by a fixed matrix. In the latter case, we propose to pre-compute the inverse of that fixed matrix instead of inverting a Hessian matrix at each iteration. To illustrate this, we write out the **U**_.*j*_ sub-problem of Gecco+ with Bernoulli log-likelihood:

$$\begin{array}{l} {\text{U}}_{.j}=\underset{{\text{U}}_{.j}}{\mathrm{argmin}}\left(\sum_{i=1}^{n}-x_{ij}u_{ij}+\log\left(1+e^{u_{ij}}\right)\right)+\frac{\rho }{2}{\left\|{\text{DU}}_{.j}-{\text{V}}_{.j}+\Lambda_{.j}\right\|}_{F}^{2}\\ \;\;\;\;\;\;\;\;\;\;\;\;\;\;\;\;\;\;+\frac{\rho }{2}{\left\|\left({\text{U}}_{.j}-{\tilde{x}}_{j}\cdot 1_{n}\right)-{\text{r}}_{j}+{\text{N}}_{.j}\right\|}_{F}^{2}\;. \end{array}$$

The Hessian is $\text{diag}\left\{\frac{e^{u_{ij}}}{{\left(1+e^{u_{ij}}\right)}^{2}}\right\}+\rho {\text{D}}^{T}\text{D}+\rho I$ which can be upper bounded by $\frac{1}{4}I+\rho {\text{D}}^{T}\text{D}+\rho I$. We propose to replace the Hessian with this fixed matrix in Newton method and use its inverse. This is closely related to the approximate Hessian literature (Krishnapuram et al., 2005; Simon et al., 2013). In this way, we just pre-compute this inverse matrix instead of inverting the Hessian matrix at each iteration, which dramatically saves computation. We give Algorithm 10 to solve Gecco+ for Bernoulli log-likelihood with a one-step update to solve the **U** sub-problem. Empirical studies show that this is faster than taking the inner nested proximal gradient approach as we generally don’t need to perform the backtracking step.

Yet, Algorithm 10 is slow as we need to run iterative inner nested ADMM updates to full convergence. To address this, as mentioned, we can take a one-step update of the inner nested iterative ADMM algorithm. To see this, we can recast the original Gecco+ problem as:

$$\begin{array}{l} \underset{U^{\left(k\right)},V}{\mathrm{minimize}}\;\ell \left(X,U\right)+\underset{P_{1}\left(V;w\right)}{\underbrace{\gamma \left(\sum_{l\in \varepsilon }w_{l}{\left\|V_{l.}\right\|}_{2}\right)}}+\alpha \underset{P_{2}\left(R\text{;}\zeta \right)}{\underbrace{\left(\sum_{j=1}^{p}\zeta_{j}{\left\|{\text{r}}_{j}\right\|}_{2}\right)}}\\ \text{subject to}\;\text{DU}-\text{V}=0,\;U-\tilde{X}=R\;. \end{array}$$

We apply multi-block ADMM to solve this optimization problem and hence get Algorithm 11. As discussed above, we take a one-step update to solve the **U** sub-problem with linearized ADMM and use the inverse of fixed approximate Hessian matrix, **M**_1_.

**Algorithm 11:** One-step inexact multi-block algorithm for Gecco+ with Bernoulli log-likelihood *ℓ_k_*:

**Precompute**: Difference matrix $\text{D},{\text{M}}_{1}={\left(\frac{1}{4}\text{I}+\rho {\text{D}}^{T}\text{D}+\rho \text{I}\right)}^{-1}$.
**while** not converged **do**
${\text{U}}^{\left(k\right)}={\text{U}}^{\left(k-1\right)}-{\text{M}}_{1}[\nabla \ell \left({\text{X},\text{U}}^{\left(k-1\right)}\right)+\rho {\text{D}}^{T}\left({\text{DU}}^{\left(k-1\right)}-{\text{V}}^{\left(k-1\right)}+\Lambda^{\left(k-1\right)}\right)+\rho \left({\text{U}}^{\left(k-1\right)}\right)-\tilde{X}-R^{\left(k-1\right)}+{\text{N}}^{\left(k-1\right)})]$
${\text{R}}^{\left(k\right)}={\text{prox}}_{\alpha /\rho P_{2}\left(\cdot ;\zeta \right)}\left({\text{U}}^{\left(k\right)}-\tilde{X}+N^{\left(k-1\right)}\right)$
${\text{N}}^{\left(k\right)}=N^{\left(k-1\right)}+\left({\text{U}}^{\left(k\right)}-\tilde{X}-R^{\left(k\right)}\right)$
${\text{V}}^{\left(k\right)}={\text{prox}}_{\gamma /\rho P_{1}\left(\cdot ;\text{w}\right)}\left({\text{DU}}^{\left(k\right)}+\Lambda^{\left(k-1\right)}\right)$
$\Lambda^{\left(k\right)}=\Lambda^{\left(k-1\right)}+\left({\text{DU}}^{\left(k\right)}-{\text{V}}^{\left(k\right)}\right)$
**end while**

Similarly, we adopt this approach to solve Gecco+ with Euclidean distances (sparse convex clustering). We first recast the original problem as:

$$\begin{array}{l} \underset{U^{\left(k\right)},V}{\mathrm{minimize}}\;\frac{1}{2}{\left\|X-U\right\|}_{2}^{2}+\gamma \underset{P_{1}\left(V;w\right)}{\underbrace{\left(\sum_{l\in \varepsilon }w_{l}{\left\|V_{l.}\right\|}_{2}\right)}}+\alpha \underset{P_{2}\left(R\text{;}\zeta \right)}{\underbrace{\left(\sum_{j=1}^{p}\zeta_{j}{\left\|{\text{r}}_{j}\right\|}_{2}\right)}}\\ \text{subject to}\;\text{DU}-\text{V}=0,\;\text{U}-\tilde{X}=R\;. \end{array}$$

Still, we use multi-block ADMM to solve this optimization problem and hence get Algorithm 12. Note that **U** sub-problem now has a closed-form solution. Typically, we do not have an approximate Hessian matrix or a closed-form solution to the sub-problem for general losses and we have to use one-step gradient descent with backtracking to solve the **U** sub-problem. Empirical study shows that this approach converges slower than Algorithm 9 which uses one-step proximal gradient descent with backtracking.

## Appendix D. Gecco+ for Non-Differentiable Losses

In this section, we propose an algorithm to solve Gecco+ when the loss *ℓ* is non-differentiable. In this case, we develop a multi-block ADMM algorithm to solve Gecco+ and prove its algorithmic convergence.

**Algorithm 12:** One-step inexact multi-block algorithm for Gecco+ with Euclidean distances *ℓ_k_*:

**Precompute:** Difference matrix $\text{D},{\text{M}}_{2}={\left(\text{I}+\rho {\text{D}}^{T}\text{D}+\rho \text{I}\right)}^{-1}$.
**while** not converged **do**
${\text{U}}^{\left(k\right)}={\text{M}}_{2}\left[\text{X}+\rho {\text{D}}^{T}\left({\text{V}}^{\left(k-1\right)}-\Lambda^{\left(k-1\right)}\right)+\rho \left(\tilde{X}+R^{\left(k-1\right)}-{\text{N}}^{\left(k-1\right)}\right)\right]$
${\text{R}}^{\left(k\right)}={\text{prox}}_{\alpha /\rho P_{2}\left(\cdot ;\zeta \right)}\left({\text{U}}^{\left(k\right)}-\tilde{X}+N^{\left(k-1\right)}\right)$
${\text{N}}^{\left(k\right)}=N^{\left(k-1\right)}+\left({\text{U}}^{\left(k\right)}-\tilde{X}-R^{\left(k\right)}\right)$
${\text{V}}^{\left(k\right)}={\text{prox}}_{\gamma /\rho P_{1}\left(\cdot ;\text{w}\right)}\left({\text{DU}}^{\left(k\right)}+\Lambda^{\left(k-1\right)}\right)$
$\Lambda^{\left(k\right)}=\Lambda^{\left(k-1\right)}+\left({\text{DU}}^{\left(k\right)}-{\text{V}}^{\left(k\right)}\right)$
**end while**

### D.1 Gecco+ Algorithm for Non-Differentiable Losses

Suppose the non-differentiable loss *ℓ* can be expressed as *ℓ*(**X**, **U**) = *f*(*g*(**X**, **U**)) where *f* is convex but non-differentiable and *g* is affine. This expression is reasonable as it satisfies the affine composition of a convex function. For example, for the least absolute loss, $f\left(Z\right)=\sum_{j=1}^{p}{\left\|{\text{z}}_{j}\right\|}_{1}={\left\|\text{vec}\left(Z\right)\right\|}_{1}$ and g(X,U)=X−U. We specify the affine function *g* as we want to augment the non-differentiable term in the loss function *ℓ*.

We can rewrite the problem as:

$$\underset{\text{U}}{\mathrm{minimize}}\;f\left(g\left(X,U\right)\right)+\gamma \sum_{1\leq i\leq i^{\prime }\leq n}w_{ii^{\prime }}{\left\|{\text{U}}_{i.}-{\text{U}}_{i^{\prime }.}\right\|}_{2}+\alpha \sum_{j=1}^{p}\zeta_{j}{\left\|{\text{U}}_{.j}-{\tilde{x}}_{j}\cdot 1_{n}\right\|}_{2}\;.$$

We can now recast the problem above as the equivalent constrained problem:

$$\begin{array}{l} \underset{U,V,Z,R}{\mathrm{minimize}}\;f\left(Z\right)+\gamma \underset{P_{1}\left(V;w\right)}{\underbrace{\left(\sum_{l\in \varepsilon }w_{l}{\left\|V_{l.}\right\|}_{2}\right)}}+\alpha \underset{P_{2}\left(R\text{;}\zeta \right)}{\underbrace{\left(\sum_{j=1}^{p}\zeta_{j}{\left\|{\text{r}}_{j}\right\|}_{2}\right)}}\\ \text{subject to}\;g\left(X,U\right)=Z\\ \;\;\;\;\;\;\text{DU}-\text{V}=0\\ \;\;\;\;\;\;U-\tilde{X}=R\;, \end{array}$$

where $\tilde{X}$ is an *n* × *p* matrix with the *j^th^* column equal to scalar ${\tilde{x}}_{j}$.

The augmented Lagrangian in scaled form is:

$$\begin{array}{l} L\left(U,V,Z,R,\Lambda ,N,\Psi \right)=\frac{\rho }{2}{\left\|\mathrm{DU}-V+\Lambda \right\|}_{F}^{2}+\frac{\rho }{2}{\left\|\left(U-\tilde{X}\right)-R+N\right\|}_{F}^{2}\\ \;\;\;\;\;\;\;\;\;+\frac{\rho }{2}{\left\|g\left(X,U\right)-Z+\Psi \right\|}_{F}^{2}+f\left(Z\right)+\gamma \sum_{l\in \varepsilon }w_{l}{\left\|{\text{V}}_{l.}\right\|}_{2}+\alpha \sum_{j=1}^{p}\zeta_{j}{\left\|{\text{r}}_{j}\right\|}_{2}\;, \end{array}$$

where the dual variable for **V** is denoted by **Λ**; the dual variable for **Z** is denoted by **Ψ**; the dual variable for **R** is denoted by **N**.

Since we assume *g* to be affine, i.e., *g*(**X**, **U**) = **AX** + **BU** + **C**, the augmented Lagrangian in scaled form can be written as:

$$\begin{array}{l} L\left(U,V,Z,R,\Lambda ,N,\Psi \right)=\frac{\rho }{2}{\left\|\mathrm{DU}-V+\Lambda \right\|}_{F}^{2}+\frac{\rho }{2}{\left\|\left(U-\tilde{X}\right)-R+N\right\|}_{F}^{2}\\ \;\;\;\;\;\;\;\;\;+\frac{\rho }{2}{\left\|\mathrm{AX}+\mathrm{BU}+C-Z+\Psi \right\|}_{F}^{2}+f\left(Z\right)+\gamma \sum_{l\in \varepsilon }\omega_{l}{\left\|V_{l\cdot }\right\|}_{2}+\alpha \sum_{j=1}^{p}\zeta_{j}{\left\|r_{j}\right\|}_{2}\;. \end{array}$$

It can be shown that the **U** sub-problem has a closed-form solution.

Note that, hinge loss is also non-differentiable and we can write *g*(**X**, **U**) = 1 − **U** ο **X** where **1** is a matrix of all one and “ο” is the Hadamard product. In this case, the **U** sub-problem does not have a closed-form solution and we will discuss how to solve this problem in the next section.

For distance-based losses, the loss function can always be written as: *ℓ*(**X**, **U**) = *f*(**X** − **U**), which means *g*(**X**, **U**) = **X** − **U**. Then the augmented Lagrangian in scaled form can be simplified as:

$$\begin{array}{l} L\left(U,V,Z,R,\Lambda ,N,\Psi \right)=\frac{\rho }{2}{\left\|\mathrm{DU}-V+\Lambda \right\|}_{F}^{2}+\frac{\rho }{2}{\left\|\left(U-\tilde{X}\right)-R+N\right\|}_{F}^{2}\\ \;\;\;\;\;\;\;\;\;+\frac{\rho }{2}{\left\|X-U-Z+\Psi \right\|}_{F}^{2}+f\left(Z\right)+\gamma \sum_{l\in \varepsilon }\omega_{l}{\left\|V_{l\cdot }\right\|}_{2}+\alpha \sum_{j=1}^{p}\zeta_{j}{\left\|r_{j}\right\|}_{2}\;. \end{array}$$

Now the **U** sub-problem has a closed-form solution:

$${\text{U}}^{\left(k\right)}={\left({\text{D}}^{T}\text{D}+2\text{I}\right)}^{-1}\left({\text{D}}^{T}\left({\text{V}}^{\left(k-1\right)}{-\text{Λ}}^{\left(k-1\right)}\right){+\tilde{X}+\text{R}}^{\left(k-1\right)}-{\text{N}}^{\left(k-1\right)}+X-{\text{Z}}^{\left(k-1\right)}+{\text{Ψ}}^{\left(k-1\right)}\right)\;.$$

This gives us Algorithm 13 to solve Gecco+ for non-differentiable distance-based loss.

**Algorithm 13:** Multi-block ADMM for non-differentiable distance-based loss

**Precompute:** Difference matrix $\text{D},\text{M}={\left({\text{D}}^{T}\text{D}+2\text{I}\right)}^{-1}$.
**while** not converged **do**
${\text{U}}^{\left(k\right)}=\text{M}\left({\text{D}}^{T}\left({\text{V}}^{\left(k-1\right)}-\Lambda^{\left(k-1\right)}\right)+\tilde{X}+R^{\left(k-1\right)}-{\text{N}}^{\left(k-1\right)}+\text{X}-Z^{\left(k-1\right)}+\Psi^{\left(k-1\right)}\right)$
$Z^{\left(k\right)}={\text{prox}}_{f/p}\left(X-U^{\left(k\right)}+\Psi^{\left(k-1\right)}\right)$
${\text{R}}^{\left(k\right)}={\text{prox}}_{\alpha /\rho P_{2}\left(\cdot ;\zeta \right)}\left({\text{U}}^{\left(k\right)}-\tilde{X}+{\text{N}}^{\left(k-1\right)}\right)$
*$\Psi^{\left(k\right)}=\Psi^{\left(k-1\right)}+\left(X-U^{\left(k\right)}-Z^{\left(k\right)}\right)$*
${\text{N}}^{\left(k\right)}=N^{\left(k-1\right)}+\left({\text{U}}^{\left(k\right)}-\tilde{X}-R^{\left(k\right)}\right)$
${\text{V}}^{\left(k\right)}={\text{prox}}_{\gamma /\rho P_{1}\left(\cdot ;\text{w}\right)}\left({\text{DU}}^{\left(k\right)}+\Lambda^{\left(k-1\right)}\right)$
$\Lambda^{\left(k\right)}=\Lambda^{\left(k-1\right)}+\left({\text{DU}}^{\left(k\right)}-{\text{V}}^{\left(k\right)}\right)$
**end while**

Algorithm 13 can be used to solve Gecco+ problem with various distances such as Manhattan, Minkowski and Chebychev distances by applying the corresponding proximal operator in the **Z**-update. For example, for Gecco+ with Manhattan distances, the **Z**-update is just applying element-wise soft-thresholding operator. For Gecco+ with Chebychev distances, the proximal operator in the **Z**-update can be computed separately across the rows of its argument and reduces to applying row-wise proximal operator of the infinity-norm. For Gecco+ with Minkowski distances, we similarly apply row-wise proximal operator of the *ℓ_q_*-norm.

Next we prove the convergence of Algorithm 13.

### D.2 Proof of Convergence for Algorithm 13

**Theorem 7** *If ℓ is convex*, Algorithm 13 *converges to a global minimum*.

**Proof:** Note we have provided a sufficient condition for the convergence of four-block ADMM in Lemma 5. Next we show that the constraint set in our problem satisfies the condition in Lemma 5 and hence the multi-block ADMM Algorithm 13 converges. Recall our problem is:

$$\begin{array}{l} \underset{U,V,Z,R}{\text{minimize}}\;f\left(Z\right)+\gamma \sum_{l\in \varepsilon }w_{l}{\left\|V_{l\cdot }\right\|}_{2}+\alpha \sum_{j=1}^{p}\zeta_{j}{\left\|r_{j}\right\|}_{2}\\ \text{subject to}\;X-U=Z\\ \;\;\;\;\;\mathrm{DU}-V=0\\ \;\;\;\;\;\text{U}-\tilde{X}=\text{R}\;. \end{array}$$

Note that Lemma 5 is stated in vector form. Hence we transform the constraints above from matrix form to vector form. Note that $DU=V\Leftrightarrow U^{T}D^{T}=V^{T}\Leftrightarrow \left(D⊗I_{p}\right)\text{vec}\left(U^{T}\right)=\text{vec}\left({\text{V}}^{T}\right)$. Hence we can write the constraints as:

$$\left(\begin{array}{c} \text{I}\\ \text{A}\\ \text{I} \end{array}\right)\text{u}+\left(\begin{array}{c} \text{I}\\ 0\\ 0 \end{array}\right)\text{z}+\left(\begin{array}{c} 0\\ 0\\ -\text{I} \end{array}\right)\text{r}+\left(\begin{array}{c} 0\\ -\text{I}\\ 0 \end{array}\right)\text{v}=\text{b}\;,$$

where $\text{u}=\text{vec}\left(U^{T}\right)$, $A=D⊗I_{p}$, $z=\text{vec}\left(Z^{T}\right)$, $\text{r}=\text{vec}\left(R^{T}\right)$, $v=\text{vec}\left(V^{T}\right)$, $b=\left(\begin{array}{c} \text{vec}\left(X^{T}\right)\\ 0_{p\times \left|\varepsilon \right|}\\ \tilde{x}\\ \vdots \\ \tilde{x} \end{array}\right)$, $\tilde{x}\in R^{p}$ is a column vector consisting of all ${\tilde{x}}_{j}$ and is repeated *n* times in **b**.

By construction, $A_{2}=\left(\begin{array}{c} I\\ 0\\ 0 \end{array}\right)$, $A_{3}=\left(\begin{array}{c} 0\\ 0\\ -I \end{array}\right)$, and $A_{4}=\left(\begin{array}{c} 0\\ -I\\ 0 \end{array}\right)$. It is easy to verify that: $A_{2}^{T}A_{3}=0$, $A_{2}^{T}A_{4}=0$, $A_{3}^{T}A_{4}=0$. Hence our setup satisfies the sufficient condition in Lemma 5 and the multi-block ADMM Algorithm 13 converges. ■

### D.3 Special Case: Gecco+ with Hinge Losses

As mentioned, we cannot directly apply Algorithm 13 to solve Gecco+ with hinge losses as the function *g*(**X**, **U**) in this case is not the same as the one in distance-based losses. Recall Gecco+ with hinge losses is:

$$\underset{U}{\text{minimize}}\;\sum_{i=1}^{n}\sum_{j=1}^{p}\max\left(0,1-u_{ij}x_{ij}\right)+\gamma \sum_{1\leq i<i^{\prime }\leq n}w_{ii^{\prime }}{\left\|U_{i_{.}}-U_{i^{\prime }.}\right\|}_{2}+\alpha \sum_{j=2}^{p}\zeta_{j}{\left\|U_{.j}-{\tilde{x}}_{j}\cdot 1_{n}\right\|}_{2}\;.$$

Like before, we can rewrite the problem as:

$$\underset{\text{U}}{\text{minimize}}\;f\left(g\left(X,U\right)\right)+\gamma \sum_{1\leq i<i^{\prime }\leq n}w_{ii^{\prime }}{\left\|U_{i\cdot }-U_{i^{\prime }\cdot }\right\|}_{2}+\alpha \sum_{j=1}^{p}\zeta_{j}{\left\|U_{\cdot j}-{\tilde{x}}_{j}\cdot 1_{n}\right\|}_{2}\;.$$

We can now recast the problem above as the equivalent constrained problem:

$$\begin{array}{l} \underset{U,V,Z,R}{\text{minimize}}\;f\left(Z\right)+\gamma \underset{P_{1}\left(V;w\right)}{\underbrace{\left(\sum_{l\in \varepsilon }w_{l}{\left\|V_{l\cdot }\right\|}_{2}\right)}}+\alpha \underset{P_{2}\left(R;\zeta \right)}{\underbrace{\left(\sum_{j=1}^{p}\zeta_{j}{\left\|r_{j}\right\|}_{2}\right)}}\\ \text{subject to}\;1-UοX=Z\\ \;\;\;\;\;\mathrm{DU}-V=0\\ \;\;\;\;\;\text{U}-\tilde{X}=\text{R}\;. \end{array}$$

Here, *f*(**Z**) = max(0, **Z**). With a slight abuse of notation, we refer *f* to applying element-wise maximum to all entries in the matrix. We set *g*(**X**, **U**) = 1 − **U** ο **X** where **1** is a matrix of all one and “ο” is the Hadamard product. $\tilde{X}$ is an *n* × *p* matrix with the *j^th^* column equal to scalar ${\tilde{x}}_{j}$.

The augmented Lagrangian in scaled form is:

$$\begin{array}{l} L\left(U,V,Z,R,\Lambda ,N,\Psi \right)=\frac{\rho }{2}{\left\|\mathrm{DU}-V+\Lambda \right\|}_{F}^{2}+\frac{\rho }{2}{\left\|\left(U-\tilde{X}\right)-R+N\right\|}_{F}^{2}\\ \;\;\;\;\;\;\;\;\;+\frac{\rho }{2}{\left\|1-UοX-Z+\Psi \right\|}_{F}^{2}+f\left(Z\right)+\gamma \sum_{l\in \varepsilon }w_{l}{\left\|{\text{V}}_{l.}\right\|}_{2}+\alpha \sum_{j=1}^{p}\zeta_{j}{\left\|{\text{r}}_{j}\right\|}_{2}\;. \end{array}$$

The **U** sub-problem now becomes:

$$\begin{array}{l} {\text{U}}^{\left(k+1\right)}=\underset{\text{U}}{\mathrm{argmin}}\;{\left\|\mathrm{DU}-V^{\left(k\right)}+\Lambda^{\left(k\right)}\right\|}_{F}^{2}+{\left\|U-\tilde{X}-R^{\left(k\right)}+N^{\left(k\right)}\right\|}_{F}^{2}\\ \;\;\;\;\;\;\;\;+{\left\|1-UοX-Z^{\left(k\right)}+\Psi^{\left(k\right)}\right\|}_{F}^{2}\;. \end{array}$$

The first-order optimality condition is:

$${\text{D}}^{T}\left(\mathrm{DU}-V^{\left(k\right)}+\Lambda^{\left(k\right)}\right)+U-\tilde{X}-R^{\left(k\right)}+N^{\left(k\right)}+Xο\left(UοX+Z^{\left(k\right)}-1-\Psi^{\left(k\right)}\right)=0\;,$$

which can be written as:

$$\begin{array}{l} \left(XοX\right)οU+Xο\left(Z^{\left(k\right)}-1-\Psi^{\left(k\right)}\right)+D^{T}DU-D^{T}\left(V^{\left(k\right)}-\Lambda^{\left(k\right)}\right)+U-\tilde{X}-R^{\left(k\right)}+N^{\left(k\right)}=0\\ \left(XοX\right)οU+\left(D^{T}DU+I\right)U=D^{T}\left(V^{\left(k\right)}-\Lambda^{\left(k\right)}\right)+\tilde{X}+R^{\left(k\right)}-N^{\left(k\right)}+Xο\left(1+\Psi^{\left(k\right)}-Z^{\left(k\right)}\right)\;. \end{array}$$

To solve **U** from the above equation, one way is to first find the SVD of the leading coefficient:

$$XοX=\sum_{k}\sigma_{k}{\tilde{u}}_{k}{\tilde{v}}_{k}=\sum_{k}{\tilde{w}}_{k}{\tilde{v}}_{k}\;.$$

From this decomposition we create two sets of diagonal matrices:

$$\begin{array}{l} {\tilde{W}}_{k}=\text{Diag}\left({\tilde{w}}_{k}\right)\\ {\tilde{V}}_{k}=\text{Diag}\left({\tilde{v}}_{k}\right)\;. \end{array}$$

The Hadamard product can now be replaced by a sum

$$\sum_{k}{\tilde{W}}_{k}{U\tilde{V}}_{k}+\left(D^{T}D+I\right)U=C\;,$$

where $C=D^{T}\left(V^{\left(k\right)}-\Lambda^{\left(k\right)}\right)+\tilde{X}+R^{\left(k\right)}-N^{\left(k\right)}+Xο\left(1+\Psi^{\left(k\right)}-Z^{\left(k\right)}\right)$.

Now we can solve this equation via vectorization:

$$\begin{array}{l} \text{vec}\left(C\right)=\left(I⊗\left(D^{T}D+I\right)+\sum_{k}{\tilde{V}}_{k}⊗{\tilde{W}}_{k}\right)\text{vec}\left(\text{U}\right)\\ \text{vec}\left(U\right)={\left(I⊗\left(D^{T}D+I\right)+\sum_{k}{\tilde{V}}_{k}⊗{\tilde{W}}_{k}\right)}^{+}\text{vec}\left(\text{C}\right)\\ \;\;\text{U}=\text{Mat}\left({\left(I⊗\left(D^{T}D+I\right)+\sum_{k}{\tilde{V}}_{k}⊗{\tilde{W}}_{k}\right)}^{+}\text{vec}\left(C\right)\right)\;, \end{array}$$

where **B**^+^ denotes the pseudo-inverse of **B**, and Mat() is the inverse of the vec() operation.

Here we have to compute the pseudo-inverse of a matrix which is computationally expensive in practice. To avoid this, we adopt generalized ADMM approach proposed by Deng and Yin (2016) where the **U** sub-problem is augmented by a positive semi-definite quadratic operator. In our case, our modified **U** sub-problem becomes:

$$\begin{array}{l} \underset{\text{U}}{\mathrm{argmin}}\;{\left\|\mathrm{DU}-{\text{V}}^{\left(k\right)}+\Lambda^{\left(k\right)}\right\|}_{F}^{2}+{\left\|\left(U-\tilde{X}\right)-R^{\left(k\right)}+N^{\left(k\right)}\right\|}_{F}^{2}+{\left\|1-UοX-Z^{\left(k\right)}+\Psi^{\left(k\right)}\right\|}_{F}^{2}\\ \;\;\;\;\;\;\;\;\;\;\;\;+{\left\|\left(1-XοX\right)ο\left(U-U^{\left(k\right)}\right)\right\|}_{F}^{2}\;. \end{array}$$

The first-order optimality condition now becomes:

$$\begin{array}{l} D^{T}\left(\mathrm{DU}-V^{\left(k\right)}+\Lambda^{\left(k\right)}\right)U-\tilde{X}-R^{\left(k\right)}+N^{\left(k\right)}+Xο\left(UοX+Z^{\left(k\right)}-1-\Psi^{\left(k\right)}\right)\\ +\left(1-XοX\right)ο\left(U-U^{\left(k\right)}\right)=0\;. \end{array}$$

We have:

$$\mathrm{HU}=D^{T}\left(V^{\left(k\right)}-\Lambda^{\left(k\right)}\right)+\tilde{X}+R^{\left(k\right)}-N^{\left(k\right)}-Xο\left(Z^{\left(k\right)}-1-\Psi^{\left(k\right)}\right)+\left(1-XοX\right)οU^{\left(k\right)}\;,$$

where **H** = (**D**^*T*^**D** + **I** + **1**). Hence we have analytical update:

$${\text{U}}^{\left(k+1\right)}={\text{H}}^{-1}\left(D^{T}\left(V^{\left(k\right)}-\Lambda^{\left(k\right)}\right)+\tilde{X}+R^{\left(k\right)}-N^{\left(k\right)}-Xο\left(Z^{\left(k\right)}-1-\Psi^{\left(k\right)}\right)+\left(1-XοX\right)ο{\text{U}}^{\left(k\right)}\right)\;.$$

It is easy to see that the **V**, **Z** and **R** updates all have closed-form solutions.

## Appendix E. Multinomial Gecco+

In this section, we briefly demonstrate how Gecco+ with multinomial losses is formulated, which is slightly different from the original Gecco+ problems. Suppose we observe categorical data as follows (*K* = 3):

$$X_{n\times p}=\left(\begin{array}{ccc} 1 & 1 & 1\\ 2 & 2 & 2\\ 3 & 3 & 3 \end{array}\right)\;.$$

We can get the indicator matrix **X**^(*k*)^ for each class *k* as:

$$X^{\left(1\right)}=\left(\begin{array}{ccc} 1 & 1 & 1\\ 0 & 0 & 0\\ 0 & 0 & 0 \end{array}\right)\;{\text{X}}^{\left(2\right)}=\left(\begin{array}{ccc} 0 & 0 & 0\\ 1 & 1 & 1\\ 0 & 0 & 0 \end{array}\right)\;{\text{X}}^{\left(3\right)}=\left(\begin{array}{ccc} 0 & 0 & 0\\ 0 & 0 & 0\\ 1 & 1 & 1 \end{array}\right)\;.$$

Then we concatenate **X**^(1)^, **X**^(2)^, **X**^(3)^ and get ${\hat{X}}_{n\times \left(p*K\right)}=\left(\begin{array}{ccc} X^{\left(1\right)} & X^{\left(2\right)} & X^{\left(3\right)} \end{array}\right)$. This is equivalent to the dummy coding of the categorical matrix $\tilde{X}$ after some row/column shuffle:

$$\tilde{X}={\left(\begin{array}{ccccccccc} 1 & 0 & 0 & 1 & 0 & 0 & 1 & 0 & 0\\ 0 & 1 & 0 & 0 & 1 & 0 & 0 & 1 & 0\\ 0 & 0 & 1 & 0 & 0 & 1 & 0 & 0 & 1 \end{array}\right)}_{n\times \left(p*K\right)}\;.$$

It is obvious that measuring the difference of two observations by comparing rows of $\hat{X}$ is better than simply comparing the Euclidean distances of rows of original data matrix **X**. Also parameterizing in $\hat{X}$ is beneficial for computing the multinomial log-likelihood or deviance. Hence we concatenate all columns of **X**^(*k*)^ as input data. Similarly, we concatenate all columns of the corresponding **U**^(*k*)^ and then fuse $\hat{U}$ in a row-wise way.

### E.1 Gecco with Multinomial Log-likelihood

Gecco with multinomial log-likelihood can be formulated as:

$$\underset{\text{U}}{\text{minimize}}\;\sum_{i}\sum_{j}\left\{\sum_{k=1}^{K}-x_{ijk}u_{ijk}+\log\left(\sum_{k=1}^{K}e^{u_{ijk}}\right)\right\}+\gamma \sum_{i<i^{\prime }}w_{ii^{\prime }}{\left\|\left[\begin{array}{c} {\text{U}}_{i\cdot }^{\left(1\right)}\\ \vdots \\ {\text{U}}_{i\cdot }^{\left(1\right)} \end{array}\right]-\left[\begin{array}{c} {\text{U}}_{i^{\prime }\cdot }^{\left(1\right)}\\ \vdots \\ {\text{U}}_{i^{\prime }\cdot }^{\left(K\right)} \end{array}\right]\right\|}_{2}\;,$$

where $x_{ijk}$ refers to the elements of indicator matrix $X_{ij}^{\left(k\right)}$ discussed previously and $U_{i.}^{\left(k\right)}=\left[\begin{array}{c} u_{i1k}\\ u_{i2k}\\ \vdots \\ u_{ipk} \end{array}\right]$ .

### E.2 Gecco+ with Multinomial Log-likelihood

Gecco+ with multinomial log-likelihood can be formulated as:

$$\begin{array}{l} \underset{\text{U}}{\text{minimize}}\;\sum_{i}\sum_{j}\left\{\sum_{k=1}^{K}-x_{ijk}u_{ijk}+\log\left(\sum_{k=1}^{K}e^{u_{ijk}}\right)\right\}+\gamma \sum_{i<i^{\prime }}w_{ii^{\prime }}{\left\|\left[\begin{array}{c} {\text{U}}_{i\cdot }^{\left(1\right)}\\ \vdots \\ {\text{U}}_{i\cdot }^{\left(K\right)} \end{array}\right]-\left[\begin{array}{c} {\text{U}}_{i^{\prime }\cdot }^{\left(1\right)}\\ \vdots \\ {\text{U}}_{i^{\prime }\cdot }^{\left(K\right)} \end{array}\right]\right\|}_{2}\\ \;\;\;\;\;\;\;+\alpha \sum_{j=1}^{p}\sum_{k=1}^{K}{\left\|U_{\cdot j}^{\left(k\right)}-{\tilde{x}}_{j}^{\left(k\right)}\cdot 1_{n}\right\|}_{2}\;, \end{array}$$

where $U_{i.}^{\left(k\right)}=\left[\begin{array}{c} u_{i1k}\\ u_{i2k}\\ \vdots \\ u_{ipk} \end{array}\right]$, $U_{.j}^{\left(k\right)}=\left[\begin{array}{c} u_{1jk}\\ u_{2jk}\\ \vdots \\ u_{njk} \end{array}\right]$ and ${\tilde{x}}_{j}^{\left(k\right)}$ is the loss-specific center for *j* variable in *k^th^* class.

## Appendix F. Loss-specific Center Calculation

In this section, we show how to calculate the loss-specific center in Table 1.

### F.1 Continuous Data

For continuous data, we consider Gecco with Euclidean distances.

#### F.1.1 Euclidean Distance

$$\underset{\text{U}}{\text{minimize}}\;\frac{1}{2}\sum_{i=1}^{n}{\left\|X_{i.}-U_{i.}\right\|}_{2}^{2}+\gamma \sum_{i<i^{\prime }}w_{ii^{\prime }}{\left\|{\text{U}}_{i.}-U_{i^{\prime }.}\right\|}_{2}$$

When total fusion occurs, $U_{i.}=U_{i^{\prime }.}$, $\forall i\neq i^{\prime }$. Let $U_{i.}=U_{i^{\prime }.}=\text{u}$. The problem above becomes:

$$\underset{\text{u}}{\text{minimize}}\;\frac{1}{2}\sum_{i=1}^{n}{\left\|X_{i.}-u\right\|}_{2}^{2}\;.$$

Taking derivative, we get:

$$\sum_{i=1}^{n}\left(X_{i.}-u\right)=0\Rightarrow u=\bar{x}\;.$$

### F.2 Count Data

For count-valued data, we consider Gecco with Poisson log-likelihood/deviance, negative binomial log-likelihood/deviance and Manhattan distances.

#### F.2.1 Poisson log-likelihood

$$\underset{\text{U}}{\text{minimize}}\;\sum_{i}^{n}\sum_{j}^{p}-x_{ij}u_{ij}+\exp\left(u_{ij}\right)+\gamma \sum_{i<i^{\prime }}w_{ii^{\prime }}{\left\|{\text{U}}_{i.}-{\text{U}}_{i^{\prime }.}\right\|}_{2}$$

When total fusion occurs, ${\text{u}}_{ij}={\text{u}}_{i^{\prime }j}$, $\forall i\neq i^{\prime }$. Let **u** be the fusion vector and $\text{u}=\left(u_{1},\cdots ,u_{p}\right)$. The problem above becomes:

$$\underset{\text{U}}{\text{minimize}}\;\sum_{i}^{n}\sum_{j}^{p}-x_{ij}u_{j}+\exp\left(u_{j}\right)\;.$$

Taking derivative, we get:

$$\sum_{i=1}^{n}-x_{ij}+n\exp\left(u_{j}\right)=0\Rightarrow \exp\left(u_{j}\right)={\bar{x}}_{j}\Rightarrow u_{j}=\log\left({\bar{x}}_{j}\right)\Rightarrow \text{u}=\text{log}\left(\tilde{x}\right)\;.$$

#### F.2.2 Poisson Deviance

$$\underset{\text{U}}{\text{minimize}}\;\sum_{i}^{n}\sum_{j}^{p}-x_{ij}\log u_{ij}+u_{ij}+\gamma \sum_{i<i^{\prime }}w_{ii^{\prime }}{\left\|{\text{U}}_{i.}-{\text{U}}_{i^{\prime }.}\right\|}_{2}$$

Let **u** be the fusion vector and **u** = (*u*_1_, ⋯ , *u_p_*). The problem above becomes:

$$\underset{\text{U}}{\text{minimize}}\;\sum_{i}^{n}\sum_{j}^{p}-x_{ij}\log u_{j}+u_{j}\;.$$

Taking derivative, we get:

$$\sum_{i=1}^{n}\frac{-x_{ij}}{u_{j}}+n=0\Rightarrow u_{j}={\bar{x}}_{j}\Rightarrow \text{u}=\tilde{x}\;.$$

#### F.2.3 Negative Binomial log-likelihood

$$\underset{\text{U}}{\text{minimize}}\;\sum_{i}^{n}\sum_{j}^{p}-x_{ij}u_{ij}+\left(x_{ij}+\frac{1}{\alpha }\right)\log\left(\frac{1}{\alpha }+e^{u_{ij}}\right)+\gamma \sum_{i<i^{\prime }}{\left\|{\text{U}}_{i.}-{\text{U}}_{i^{\prime }.}\right\|}_{2}$$

When total fusion occurs, ${\text{u}}_{ij}={\text{u}}_{i^{\prime }j}$, $\forall i\neq i^{\prime }$. Let **u** be the fusion vector and $\text{u}=\left(u_{1},\cdots ,u_{p}\right)$. The problem above becomes:

$$\underset{\text{U}}{\text{minimize}}\;\sum_{i}^{n}\sum_{j}^{p}-x_{ij}u_{j}+\left(x_{ij}+\frac{1}{\alpha }\right)\log\left(\frac{1}{\alpha }+e^{u_{j}}\right)\;.$$

Taking derivative, we get:

$$\begin{array}{l} \sum_{i=i}^{n}-x_{ij}+\left(x_{ij}+\frac{1}{\alpha }\right)\frac{e^{u_{j}}}{\frac{1}{\alpha }+e^{u_{j}}}=0\\ \;\;\;\;\;\;\;\;\sum_{i=i}^{n}x_{ij}=\sum_{i=i}^{n}\left(x_{ij}+\frac{1}{\alpha }\right)\frac{e^{u_{j}}}{\frac{1}{\alpha }+e^{u_{j}}}\\ \;\sum_{i=i}^{n}x_{ij}\cdot \frac{1}{\alpha }+\sum_{i=i}^{n}x_{ij}\cdot e^{u_{j}}=\sum_{i=i}^{n}x_{ij}\cdot e^{u_{j}}+\frac{n}{\alpha }e^{u_{j}}\\ \;\;\;\;\;\;\;\;\;e^{u_{j}}=\sum_{i=1}^{n}x_{ij}/n\\ \;\;\;\;\;\;\;\;\exp\left(u_{j}\right)={\bar{x}}_{j}\Rightarrow u_{j}=\log\left({\bar{x}}_{j}\right)\Rightarrow \text{u}=\text{log}\left(\bar{x}\right)\;. \end{array}$$

#### F.2.4 Negative Binomial Deviance

$$\underset{\text{U}}{\text{minimize}}\;\sum_{i}^{n}\sum_{j}^{p}x_{ij}\log\left(\frac{x_{ij}}{u_{ij}}\right)-\left(x_{ij}+\frac{1}{\alpha }\right)\log\left(\frac{1+\alpha x_{ij}}{1+\alpha u_{ij}}\right)+\gamma \sum_{i<i^{\prime }}w_{ii^{\prime }}{\left\|U_{i.}-U_{i^{\prime }.}\right\|}_{2}$$

The formulation above is equivalent to:

$$\underset{\text{U}}{\text{minimize}}\;\sum_{i}^{n}\sum_{j}^{p}-x_{ij}\log u_{ij}+\left(x_{ij}+\frac{1}{\alpha }\right)\log\left(1+\alpha x_{ij}\right)+\gamma \sum_{i<i^{\prime }}w_{ii^{\prime }}{\left\|U_{i.}-U_{i^{\prime }.}\right\|}_{2}\;.$$

Let **u** be the fusion vector and $\text{u}=\left(u_{1},\cdots ,u_{p}\right)$. The problem above becomes:

$$\underset{\text{U}}{\text{minimize}}\;\sum_{i}^{n}\sum_{j}^{p}-x_{ij}\log u_{j}+\left(x_{ij}+\frac{1}{\alpha }\right)\log\left(1+\alpha u_{j}\right)\;.$$

Taking derivative, we get:

$$\begin{array}{l} \sum_{i=1}^{n}\frac{-x_{ij}}{u_{j}}+\frac{x_{ij}+\frac{1}{\alpha }}{1+\alpha u_{j}}\cdot \alpha =0\\ \sum_{i}^{n}x_{ij}+\alpha u_{j}\sum_{i=1}^{n}x_{ij}=nu_{j}+\alpha u_{j}\sum_{i=1}^{n}x_{ij}\\ u_{j}={\bar{x}}_{j}\Rightarrow \text{u}=\bar{\text{x}}\;. \end{array}$$

#### F.2.5 Manhattan Distance

$$\underset{\text{U}}{\text{minimize}}\;\sum_{i=1}^{n}{\left\|{\text{x}}_{i}-u_{i}\right\|}_{1}+\gamma \sum_{i<i^{\prime }}w_{ii^{\prime }}{\left\|U_{i.}-U_{i^{\prime }.}\right\|}_{2}$$

When total fusion occurs, ${\text{U}}_{i.}={\text{U}}_{i^{\prime }.}$, $\forall i\neq i^{\prime }$. Let $U_{i.}=U_{i^{\prime }.}=\text{u}$. We have:

$$\underset{\text{U}}{\text{minimize}}\;\sum_{i=1}^{n}{\left\|{\text{x}}_{i}-\text{u}\right\|}_{1}\;.$$

For each *j*, we have:

$$\underset{u_{j}}{\mathrm{minimize}}\;\sum_{i=1}^{n}{\left\|x_{ij}-u_{j}\right\|}_{1}\;.$$

We know that the optimal *u_j_* is just the median of *x_ij_* for each *j*.

### F.3 Binary Data

For binary data, we consider Gecco with Bernoulli log-likelihood, binomial deviance and hinge loss.

#### F.3.1 Bernoulli log-likelihood

$$\underset{\text{U}}{\mathrm{minimize}}\;\sum_{i}^{n}\sum_{j}^{p}-x_{ij}u_{ij}+\log\left(1+e^{u_{ij}}\right)+\gamma \sum_{i<i^{\prime }}w_{ii^{\prime }}{\left\|U_{i.}-U_{i^{\prime }.}\right\|}_{2}$$

When total fusion occurs, ${\text{u}}_{ij}={\text{u}}_{i^{\prime }j}$, $\forall i\neq i^{\prime }$. Let **u** be the fusion vector and $\text{u}=\left(u_{1},\cdots ,u_{p}\right)$. The problem above becomes:

$$\underset{U}{\mathrm{minimize}}\;\sum_{i}^{n}\sum_{j}^{p}-x_{ij}u_{j}+\log\left(1+e^{u_{i}}\right)\;.$$

Taking derivative, we get:

$$\begin{array}{l} \sum_{i=1}^{n}-x_{ij}+n\frac{\exp\left(u_{j}\right)}{1+\exp\left(u_{j}\right)}=0\Rightarrow \frac{\exp\left(u_{j}\right)}{1+\exp\left(u_{j}\right)}={\bar{x}}_{j}\\ \;\;\;\;\;\;\;\;\;\;\;\Rightarrow {\mathrm{logit}}^{-1}\left(u_{j}\right)={\bar{x}}_{j}\Rightarrow u_{j}=\mathrm{logit}\left({\bar{x}}_{j}\right)\Rightarrow u=\mathrm{logit}\left(\text{x}\right)\;. \end{array}$$

#### F.3.2 Binomial Deviance

$$\underset{U}{\mathrm{minimize}}\;\sum_{i}^{n}\sum_{j}^{p}-x_{ij}\log u_{ij}-\left(1-x_{ij}\right)\log\left(1-u_{ij}\right)+\gamma \sum_{i<i^{\prime }}w_{ii^{\prime }}{\left\|U_{i.}-U_{i^{\prime }.}\right\|}_{2}$$

Let **u** be the fusion vector and $\text{u}=\left(u_{1},\cdots ,u_{p}\right)$. The problem above becomes:

$$\underset{U}{\mathrm{minimize}}\;\sum_{i}^{n}\sum_{j}^{p}-x_{ij}\log u_{j}-\left(1-x_{ij}\right)\log\left(1-u_{j}\right)\;.$$

Taking derivative, we get:

$$\begin{array}{l} \sum_{i=1}^{n}\frac{-x_{ij}}{u_{j}}+\frac{1-x_{ij}}{1-u_{j}}=0\Rightarrow \sum_{i}^{n}-x_{ij}\left(1-u_{j}\right)+\left(1-x_{ij}\right)u_{j}=0\\ \;\;\;\;\;\;\;\;\;\Rightarrow \sum_{i}^{n}-x_{ij}+u_{j}=0\Rightarrow u_{j}={\bar{x}}_{j}\Rightarrow \text{u}=\bar{x}\;. \end{array}$$

#### F.3.3 Hinge Loss

$$\begin{array}{cc} \underset{\text{U}}{\text{minimize}} & \sum_{i=1}^{n}\sum_{j=1}^{p}\max\left(0,1-u_{ij}x_{ij}\right)+\gamma \sum_{i<i'}\omega_{ii'}{\left\|{\text{U}}_{i.}-{\text{U}}_{i'.}\right\|}_{2} \end{array}$$

Let **u** be the fusion vector and $\text{u}=\left(u_{1},\cdots ,u_{p}\right)$. The problem above becomes:

$$\begin{array}{cc} \underset{\text{U}}{\text{minimize}} & \sum_{i=1}^{n}\sum_{j=1}^{p}\max\left(0,1-u_{j}x_{ij}\right) \end{array}\;.$$

For each feature *j*, the problem becomes:

$$\begin{array}{cc} \underset{u_{j}}{\text{minimize}} & \sum_{i=1}^{n}\max\left(0,1-u_{j}x_{ij}\right) \end{array}\;.$$

Note in hinge loss, $x_{ij}\in \left\{-1,1\right\}$. Suppose we have *n*_1_ observations for class “1” and *n*_2_ observations for class “−1”. The problem now becomes:

$$\begin{array}{cc} \underset{u_{j}}{\text{minimize}} & n_{1}\max\left(0,1-u_{j}\right)+n_{2}\max\left(0,1+u_{j}\right) \end{array}\;.$$

Define $h\left(t\right)=n_{1}\max\left(0,1-t\right)+n_{2}\max\left(0,1+t\right)$. We have:

$$h\left(t\right)=\{\begin{array}{lll} n_{1}\left(1-t\right) & \text{if} & t\leq -1\\ n_{1}\left(1-t\right)+n_{2}\left(1+t\right) & \text{if} & -1<t<1\\ n_{2}\left(1+t\right) & \text{if} & t\geq 1\;. \end{array}$$

Clearly, if *n*_2_ > *n*_1_ (more “−1”), *h*(*t*) is minimized by *t* = −1; if *n*_1_ > *n*_2_ (more “1”), *h*(*t*) is minimized by *t* = 1; if *n*_1_ = *n*_2_, *h*(*t*) is minimized by any *t* between [−1, 1]. Therefore, *u_j_* should be the mode of all observations for feature *j*:

$$u_{j}={\text{mode}}_{i}\left(x_{ij}\right)\;.$$

### F.4 Categorical Data

For categorical data, we consider Gecco with multinomial log-likelihood and deviance.

#### F.4.1 Multinomial log-likelihood

$$\begin{array}{cc} \underset{\text{U}}{\text{minimize}} & \sum_{i}^{n}\sum_{j}^{p}\left\{\sum_{k=1}^{K}-x_{ijk}u_{ijk}+\log\left(\sum_{k=1}^{K}e^{u_{ijk}}\right)\right\} \end{array}+\gamma \sum_{i<i\prime }\omega_{ii\prime }{\left\|\left[\begin{array}{c} {\text{U}}_{i.}^{\left(1\right)}\\ \vdots \\ {\text{U}}_{i.}^{\left(K\right)} \end{array}\right]-\left[\begin{array}{c} {\text{U}}_{i\prime .}^{\left(1\right)}\\ \vdots \\ {\text{U}}_{i\prime .}^{\left(K\right)} \end{array}\right]\right\|}_{2}$$

When total fusion occurs, ${\text{u}}_{ijk}={\text{u}}_{i^{\prime }jk}$, $\forall i\neq i^{\prime }$. Let **u** be the fusion vector and ${\text{u}}_{k}=\left(u_{1k},\cdots ,u_{pk}\right)$. The problem above becomes:

$$\begin{array}{cc} \underset{\text{U}}{\text{minimize}} & \sum_{i}^{n}\sum_{j}^{p}\left\{\sum_{k}^{K}-x_{ijk}u_{jk}+\log\left(\sum_{k}^{K}e^{u_{jk}}\right)\right\} \end{array}\;.$$

Taking derivative with respect to $u_{jk}$, we get:

$$\begin{array}{l} \sum_{i=1}^{n}-x_{ijk}+n\frac{\exp\left(u_{jk}\right)}{\sum_{k}^{K}\exp\left(u_{jk}\right)}=0\\ \;\;\;\;u_{jk}=\log\frac{{\bar{x}}_{.jk}}{\sum_{k}{\bar{x}}_{.jk}}=\text{mlogit}\left({\bar{x}}_{.jk}\right)\;. \end{array}$$

#### F.4.2 Multinomial Deviance

$$\begin{array}{l} \begin{array}{cc} \underset{\text{U}}{\text{minimize}} & \sum_{i}^{n}\sum_{j}^{p}\left\{\sum_{k}^{K}-x_{ijk}\log\left(u_{ijk}\right)\right\} \end{array}+\gamma {\left\|\left[\begin{array}{c} {\text{U}}_{i.}^{\left(1\right)}\\ \vdots \\ {\text{U}}_{i.}^{\left(K\right)} \end{array}\right]-\left[\begin{array}{c} {\text{U}}_{i'.}^{\left(1\right)}\\ \vdots \\ {\text{U}}_{i'.}^{\left(K\right)} \end{array}\right]\right\|}_{2}\\ \begin{array}{cc} \text{subject to} & \sum_{k=1}^{K}u_{ijk}=1 \end{array} \end{array}$$

When total fusion occurs, ${\text{u}}_{ijk}={\text{u}}_{i^{\prime }jk}$, $\forall i\neq i^{\prime }$. Let **u** be the fusion vector and ${\text{u}}_{k}=\left(u_{1k},\cdots ,u_{pk}\right)$. The problem above becomes:

$$\begin{array}{l} \begin{array}{cc} \underset{\text{U}}{\text{minimize}} & \sum_{i}^{n}\sum_{j}^{p}\sum_{k}^{K}-x_{ijk} \end{array}\log u_{jk}\\ \begin{array}{cc} \text{subject to} & \sum_{k=1}^{K}u_{jk}=1 \end{array}\;. \end{array}$$

We can write the constraint in Lagrangian form:

$$\begin{array}{cc} \underset{\text{U}}{\text{minimize}} & \sum_{i}^{n}\sum_{j}^{p}\sum_{k}^{K}-x_{ijk} \end{array}\log u_{jk}+\lambda \left(\sum_{k=1}^{K}u_{jk}-1\right)\;.$$

Taking derivative with respect to $u_{jk}$, we get:

$$\begin{array}{l} \;\;\sum_{i=1}^{n}\frac{-x_{ijk}}{u_{jk}}+\lambda =0\\ \sum_{i=1}^{n}x_{ijk}=\lambda u_{jk}\;. \end{array}$$

We have:

$$n=\sum_{i=1}^{n}\sum_{k=1}^{K}x_{ijk}=\sum_{k=1}^{K}\sum_{i=1}^{n}x_{ijk}=\sum_{k=1}^{K}\lambda u_{jk}=\lambda \;.$$

Therefore,

$$u_{jk}=\sum_{i=1}^{n}x_{ijk}/n\;.$$

## Appendix G. Visualization of Gecco+ for Authors Data

Figure 5 illustrates selected features and cluster assignment for authors data set with one combination of *α* and *γ*. We select meaningful features and achieve satisfactory clustering results. We have already discussed the results and interpretation in detail in Section 5.1.

## Appendix H. Multi-omics Data

In this section, we show the distribution of data from different platforms in Section 5.3. We see that both gene expression data and protein data appear Gaussian; methylation data is between [0, 1]; miRNA data is highly-skewed.

## Appendix I. Null Deviance

In this appendix, we list the null deviance *D* for some common losses used in iGecco. Recall in the iGecco formulation, *π_k_*, which are set inversely proportional to the null deviance evaluated at the loss-specific center, are scaling factors to ensure that the losses are measured at the same scale in the objective function.

By definition, the null deviance *D* evaluated at the loss-specific center, refers to $\ell (X,\tilde{X})$ where each *j^th^* column of $\tilde{X}$ is the loss-specific center ${\tilde{x}}_{j}^{\left(k\right)}$ for that column/feature. Table 12 shows the null deviance *D* for some common losses.

**Table 12:** Null deviance for common losses used in iGecco

Loss Function	Null Deviance *D*

Euclidean distance	${\left\|X-\bar{X}\right\|}_{2}^{2}=\sum_{i}\sum_{j}{\left(x_{ij}-{\bar{x}}_{j}\right)}^{2}$
Manhattan distance	$\sum_{i}\sum_{j}\left|x_{ij}-\mathrm{median}\left(x_{j}\right)\right|$
Bernoulli log-likelihood	$2\left[\sum_{i}\sum_{j}-x_{ij}\mathrm{logit}\left({\bar{x}}_{j}\right)+\log\left(1+e^{\mathrm{logit}\left({\bar{x}}_{j}\right)}\right)\right]$
Binomial deviance	$2\left[\sum_{i}\sum_{j}-x_{ij}\log{\bar{x}}_{j}-\left(1-x_{ij}\right)\log\left(1-{\bar{x}}_{j}\right)\right]$

Here ${\bar{x}}_{j}$ refers to the mean for the *j^th^* column/feature while median(*x_j_*) refers to the median for the *j^th^* column. Simple calculation shows that the Bernoulli log-likelihood is equivalent to binomial deviance by noting $\mathrm{logit}\left({\bar{x}}_{j}\right)=\log\frac{{\bar{x}}_{j}}{1-{\bar{x}}_{j}}$.

## Appendix J. Choice of Tuning Parameters

In this appendix, we propose two different approaches to select tuning parameters *γ* and *α* in the iGecco+ problem; *γ* controls the number of clusters while *α* controls the number of features selected. We first consider stability selection based approach, which has been shown to enjoy nice statistical properties such as selection consistency (Wang, 2010; Fang and Wang, 2012) and been adopted in practice with strong empirical performance (Wang et al., 2018). Next, to reduce computation, we consider information criterion based approaches. Finally, we demonstrate empirical results when the number of clusters and features are not fixed but estimated based on the data.

### J.1 Stability Selection Based Approach

We first adopt the stability selection based approach for tuning parameter selection and follow closely the approach described in the work of Wang (2010); Fang and Wang (2012). We choose the stability selection based approach because i) its selection consistency has been established and ii) Wang et al. (2018) adopted similar approach for tuning parameter selection and demonstrated strong empirical performance. The rationale behind stability selection is that a good clustering algorithm with optimal tuning parameter should produce clusterings that do not vary much with respect to a small perturbation to the training samples.

By Wang (2010); Fang and Wang (2012), a clustering *ψ*(*x*) is defined as a mapping $\psi :R^{p}\to \{1,\ldots ,q\}$ where *q* is the given number of clusters. Here, we use *q* as the number of clusters since we have already used *k* to represent the *k^th^* data view **X**^(*k*)^. A clustering algorithm **Ψ**(·; *q*) with a given number of clusters *q* ≥ 2 yields a clustering mapping *ψ*(*x*) when applied to a sample. Here, we choose the number of clusters *q*, which is equivalent to choosing optimal *γ* since we can yield the cluster assignment and corresponding *q* for each *γ*. To account for the tuning parameter *α* for feature selection, we further denote the clustering algorithm as **Ψ**(·; *q, α*). Then, in our case, for any given pair of *q* and *α*, two clustering results can be obtained from two sets of bootstrapped samples. Then the clustering distance *d*, defined by Fang and Wang (2012), can be computed to measure the dissimilarity between two clustering results. We repeat the procedure multiple times and the optimal tuning parameter pair $\left(\hat{q},\hat{\alpha }\right)$ is the one that minimizes the average clustering instability. This gives Algorithm 14, which uses stability selection to choose tuning parameters.

**Algorithm 14:** Choice of number of clusters *q* and feature penalty *γ* in Gecco+/iGecco+ using stability selection

1. Generate *B* independent bootstrap sample-pairs $\left(X_{b},{\tilde{X}}_{b}\right)$, b=1,⋯,B.
2. Construct $\Psi_{X_{b,q,\alpha }}$ and $\Psi_{{\tilde{X}}_{b,q,\alpha }}$ based on $\left(X_{b},{\tilde{X}}_{b}\right)$, b=1,⋯,B.
3. For each pair, $\Psi_{X_{b,q,\alpha }}$ and $\Psi_{{\tilde{X}}_{b,q,\alpha }}$, calculate their clustering distance $d\left(\Psi_{X_{b,q,\alpha }},\Psi_{{\tilde{X}}_{b,q,\alpha }}\right)$ defined by Fang and Wang (2012). Then the clustering instability S(Ψ,q,α) can be estimated by $${\hat{S}}_{B}\left(\Psi ,q,\alpha \right)=\frac{1}{B}\sum_{b=1}^{B}d\left(\Psi_{X_{b,q,\alpha }},\Psi_{{\tilde{X}}_{b,q,\alpha }}\right)\;.$$
4. Finally, the optimal number of clusters *q* and feature penalty *α* can be estimated by $\left(\hat{q},\hat{\alpha }\right)=\underset{2\leq q\leq Q,\alpha }{\mathrm{argmin}}{\hat{S}}_{B}\left(\Psi ,q,\alpha \right)\;.$

### J.2 Information Criterion Based Approach

While choosing number of clusters based on stability selection has been shown to enjoy nice properties such as selection consistency (Wang, 2010), such methods are always computationally burdensome. To address this, on the other hand, information criterion based approach has been proposed for tuning parameter selection in convex clustering (Tan and Witten, 2015). We adopt the similar approach and propose the Bayesian information criterion (BIC) based approach to choose optimal number of clusters and features; this gives Algorithm 15.

**Algorithm 15:** Choice of number of clusters *q* and feature penalty *α* in Gecco+/iGecco+ using BIC

Initialize: $\hat{s}=\sum_{k=1}^{K}p_{k}$, ${\hat{S}}_{k}=\left\{1,\cdots ,p_{k}\right\}$, $\hat{\alpha }=1$.
1. Fit iGecco+ with a sequence of *γ* and fixed $\hat{\alpha }$. Choose the number of clusters $\hat{q}$ using BIC. $$\hat{q}=\underset{q}{\mathrm{argmin}}\;n\cdot \sum_{k=1}^{K}\pi_{k,{\hat{S}}_{k}}\ell_{k}\left(X_{{\hat{S}}_{k}}^{\left(k\right)},{\hat{U}}_{{\hat{S}}_{k}}^{\left(k\right)}\right)+q\log\left(\hat{s}n\right)\;.$$
2. Fit iGecco+ with a sequence of *α* and fixed number of clusters $\hat{q}$. Choose number of features *s* using BIC. Get corresponding $\hat{\alpha }$, active set ${\hat{S}}_{k}$ and total number of selected features $\hat{s}=\sum_{k=1}^{K}\left|{\hat{S}}_{k}\right|$. $$\hat{s}=\underset{s}{\mathrm{argmin}}\;n\cdot \sum_{k=1}^{K}\sum_{j=1}^{p_{k}}\frac{\ell_{k}\left(X_{.j}^{\left(k\right)},{\hat{U}}_{.j}^{\left(k\right)}\right)}{\ell_{k}\left(X_{.j}^{\left(k\right)},{\hat{X}}_{.j}^{\left(k\right)}\right)}+s\hat{q}\log\left(n\right)\;.$$
3. Repeat Step 1 and 2 until $\hat{q}$ and $\hat{\alpha }$ stabilize.

Here, the submatrix $X_{{\hat{S}}_{k}}^{\left(k\right)}$ corresponds to the subset of features (columns) of **X**^(*k*)^ that are in the active set ${\hat{S}}_{k}$ selected by iGecco+. The quantity $\pi_{k,{\hat{S}}_{k}}$ refers to *π_k_* calculated on the set of selected features ${\hat{S}}_{k}$, i.e., $\frac{1}{\ell_{k}\left({\text{X}}_{{\hat{S}}_{k}}^{\left(k\right)},{\tilde{X}}_{{\hat{S}}_{k}}^{\left(k\right)}\right)}$.

In summary, in step 1, we choose number of clusters only based on the selected features; in step 2, we choose number of features with the optimal number of clusters $\hat{q}$ estimated from the previous step. Note in step 1, ${\hat{U}}_{{\hat{S}}_{k}}^{\left(k\right)}$ is a function of number of clusters *q* while in step 2, ${\hat{U}}_{.j}^{\left(k\right)}$ is a function of whether the feature is selected. Specifically, in step 1, since all features in ${\hat{S}}_{k}$ are selected, ${\hat{U}}_{{\hat{S}}_{k}}^{\left(k\right)}$ correspond to the cluster centroids for each cluster and change with respect to the number of clusters. In step 2, by iGecco+, if the *j^th^* feature in the *k^th^* data view is selected, ${\hat{U}}_{.j}^{\left(k\right)}$ still correspond to the cluster centroids which are different for observations across different clusters; if the *j^th^* feature is not selected, ${\hat{U}}_{.j}^{\left(k\right)}$ corresponds to the same constant, i.e., loss-specific center ${\hat{X}}_{.j}^{\left(k\right)}$ for that feature.

The criterion to minimize is inspired by the BIC approach for convex clustering proposed by Tan and Witten (2015). Notice that we use different criterion in step 1 and 2 to choose number of clusters and features respectively. When choosing the number of clusters, we use the same loss function as in iGecco since all the features are selected. When choosing the number of features, for the presence of noise features, we find that the weighted loss function with respect to each feature in step 2 works better, as the criterion in step 1 downweights the contribution of signal features by adding informative terms along with the noise terms in the denominator. (When a feature is not selected, $\ell_{k}\left(X_{.j}^{\left(k\right)},{\hat{U}}_{.j}^{\left(k\right)}\right)$ equals $\ell_{k}\left(X_{.j}^{\left(k\right)},{\tilde{X}}_{.j}^{\left(k\right)}\right)$; when a feature is selected, $\ell_{k}\left(X_{.j}^{\left(k\right)},{\hat{U}}_{.j}^{\left(k\right)}\right)$ is less than $\ell_{k}\left(X_{.j}^{\left(k\right)},{\tilde{X}}_{.j}^{\left(k\right)}\right)$; if we use the unweighted criterion in step 1, the noise terms will dominate this criterion given a large number of noise features.) The degrees of freedom for choosing number of clusters in step 1 is *q* while in step 2, the degrees of freedom is $s\hat{q}$ since we need to estimate $\hat{q}$ number of cluster centroids for each selected feature.

Stability selection is known to be more stable in terms of choosing number of clusters, with selection consistency theoretically established in literature. Meanwhile, BIC works better in choosing number of features in practice and saves much more computation compared with stability selection. Hence, to take full advantage of both approaches, we propose a sequential tuning parameter selection procedure, outlined in Algorithm 16. We demonstrate the clustering and variable selection accuracy results in Appendix J.3 using the proposed two tuning parameter selection approaches.

**Algorithm 16:** Choice of number of clusters *q* and feature penalty *α* in Gecco+/iGecco+ using stability selection + BIC

1. Choose the number of clusters $\hat{q}$ using Algorithm 14 with *α* = 1 (stability selection).
2. Fit iGecco+ with a sequence of *α* and fixed number of clusters $\hat{q}$. Choose *α* using BIC based on Step 2 of Algorithm 15.

Based on the algorithms above for tuning parameter selection and the adaptive iGecco+ Algorithm 1 (with oracle number of clusters and features) to choose the feature weights, we propose Algorithm 17, the alternative adaptive iGecco+ when the number of clusters or features are not known a priori. Notice that in step 2, we do not perform tuning parameter selection for number of clusters *q*, as we are only interested in some type of adaptive feature selection to weigh the features.

**Algorithm 17:** Adaptive iGecco+ when the number of clusters or features are not known a priori

1. Fit iGecco+ with *α* = 1, $\zeta_{j}^{\left(k\right)}=1$ and a sequence of *γ*.
2. Find *γ* which gives non-trivial number of clusters, say *q* = 2; Get the estimate ${\hat{U}}^{\left(k\right)}$.
3. Update the feature weights ${\hat{\zeta }}_{j}^{\left(k\right)}=1/{\left\|{\hat{U}}_{.j}^{\left(k\right)}-{\tilde{x}}_{j}^{\left(k\right)}\cdot 1_{n}\right\|}_{2}$ and fusion weights ${\hat{w}}_{ij}=I_{ij}^{k}\exp\left(-\phi \hat{d}\left(X_{i.},X_{i^{\prime }.}\right)\right)$, where $\hat{d}\left(X_{i.},X_{i^{\prime }.}\right)=\sum_{k=1}^{K}\sum_{j=1}^{p_{k}}\frac{{\left\|{\hat{U}}_{.j}^{\left(k\right)}-{\tilde{x}}_{j}^{\left(k\right)}\cdot 1_{n}\right\|}_{2}}{\max_{j}{\left\|{\hat{U}}_{.j}^{\left(k\right)}-{\tilde{x}}_{j}^{\left(k\right)}\cdot 1_{n}\right\|}_{2}}\cdot \frac{1}{\pi_{k}}\cdot d_{ii^{\prime }j}^{\left(k\right)}$.
Fit adaptive iGecco+ with $\hat{\zeta }$, $\hat{w}$ and a sequence of *γ* and *α*; Find optimal *γ* and *α* using the tuning parameter selection procedure in Algorithm 15 or 16.

### J.3 Empirical Results in Simulation and Real Data Example

In this section, we demonstrate the empirical results when the number of clusters and features are not fixed but estimated based on the data. Specifically, we apply the two tuning parameter selection schemes proposed above: i) BIC based approach (Algorithm 17 + 15) and ii) stability selection + BIC based approach (Algorithm 17 + 16). We show the results of the tables in Section 4 and 5 when the number of clusters and features are estimated and not fixed to be the oracle. For simulation studies, we show the results of iGecco and iGecco+; Gecco and Gecco+ are special cases of these two. Moreover, we apply the proposed two tuning parameter selection approaches to the three real data examples in Section 5.

For iGecco, the stability selection based approach simplifies to choosing the number of clusters *q* in Algorithm 14; the BIC based approach refers to using the criterion in step 1 of Algorithm 15 to choose the number of clusters.

For iGecco+, we show the overall F1-score and number of selected features across all three data views. Recall that each data view has 10 true features and hence there are 30 true features in total. Also, the optimal number of clusters is *q* = 3. We compare the results with iClusterPlus when the number of clusters and features are not fixed. Note we only include estimated number of clusters for iClusterPlus as there is no tuning parameter for the number of selected features. (The authors mentioned feature selection could be achieved by selecting the top features based on Lasso coefficient estimates.)

Table 13 and 14 show the results of Table 3, 4 and 5 in Section 4.2 when the number of clusters (and features in iGecco+) is not fixed but estimated based on the data. Table 13 and 14 suggest that our proposed BIC based approach selects the correct number of clusters and features most of the time. On the other hand, information criterion based approaches save much more computation than stability selection. Hence, we recommend the BIC based approach for choosing tuning parameters which demonstrates strong empirical performance and saves computation. Yet, one might have their own justified choice of approach or information criterion to select tuning parameters.

**Table 13:** Adjusted Rand index and estimated number of clusters of iGecco and iCluster for mixed multi-view data of Table 3 in Section 4.2 when the number of clusters is not fixed but estimated based on the data.

	S1		S2	
	ARI	# of Clusters	ARI	# of Clusters
iGecco with BIC	0.93 (5.2e-3)	3.10 (1.0e-1)	0.98 (2.2e-2)	3.00 (0.0e-0)
iGecco with Stability Selection	0.92 (8.1e-3)	3.20 (2.0e-1)	0.89 (4.1e-2)	4.00 (3.9e-1)
iCluster+ with λ = 0	0.92 (4.1e-2)	3.20 (2.0e-1)	0.67 (1.9e-2)	3.40 (1.6e-1)

**Table 14:** Adjusted Rand index, F_1_ score, along with estimated number of clusters and features of adaptive iGecco+ and iClusterPlus for high-dimensional mixed multi-view data of Table 4 and 5 in Section 4.2 when the number of clusters and features are not fixed but estimated based on the data. We only include estimated number of clusters for iClusterPlus as there is no tuning parameter for the number of selected features.

	S3		S4		S5		S6	
	ARI	# of Clusters	ARI	# of Clusters	ARI	# of Clusters	ARI	# of Clusters
Algorithm 17 + 15 (BIC)	0.97 (7.8e-3)	3.00 (0.0e-0)	0.97 (1.3e-2)	3.10 (1.0e-1)	1.00 (0.0e-0)	3.00 (0.0e-0)	1.00 (0.0e-0)	3.00 (0.0e-0)
Algorithm 17 + 16 (SS+BIC)	0.93 (4.1e-2)	2.90 (1.0e-1)	0.89 (5.5e-2)	2.80 (1.3e-1)	0.99 (1.2e-2)	3.10 (1.0e-1)	0.82 (8.1e-2)	4.90 (9.1e-1)
iCluster+	0.53 (8.1e-2)	2.70 (3.0e-1)	0.72 (5.3e-2)	3.50 (3.1e-1)	0.63 (2.4e-2)	3.50 (2.2e-1)	0.60 (1.4e-2)	3.30 (1.5e-1)

	S3		S4		S5		S6	
	F1-score	# of Features	F1-score	# of Features	F1-score	# of Features	F1-score	# of Features

Algorithm 17 + 15 (BIC)	0.93 (1.1e-2)	30.00 (1.1e-0)	0.95 (1.9e-2)	31.60 (7.2e-1)	0.99 (6.3e-3)	30.50 (4.0e-1)	0.99 (6.5e-3)	30.90 (4.1e-1)
Algorithm 17 + 16 (SS+BIC)	0.93 (1.2e-2)	29.60 (1.2e-0)	0.96 (1.5e-2)	29.40 (6.5e-1)	0.99 (6.3e-3)	30.50 (4.0e-1)	0.99 (6.0e-3)	30.10 (3.8e-1)

Also, we apply the proposed two tuning parameter selection approaches to the real data examples in Section 5. Table 15 shows the estimated number of clusters. Again, the BIC based approach selects the correct number of clusters for all three cases.

**Table 15:** Adjusted Rand index, along with estimated number of clusters using adaptive (i)Gecco+ for real data of Table 6, 8 and 10 in Section 5 when the number of clusters and features are not fixed but estimated based on the data. For the first two data set, we show the results of Manhattan Gecco+.

	Authors Data		TCGA Data		Multi-omics Data	
	ARI	# of Clusters	ARI	# of Clusters	ARI	# of Clusters
Algorithm 17 + 15 (BIC)	0.96	4.00	0.76	3.00	0.71	3.00
Algorithm 17 + 16 (SS+BIC)	0.96	4.00	0.59	2.00	0.52	2.00

## Appendix K. Stable to Perturbations of Data

In this appendix, we demonstrate that clustering assignments of iGecco+ are stable to perturbations in the data as shown in Proposition 2 of Section 2.4.

To show this, we include a simulation study similar to the one by Chi et al. (2017). We first apply adaptive iGecco+ on the original data to obtain baseline clustering. Then we add i.i.d. noise to each data view to create a perturbed data set on which we apply the same iGecco+ method. Specifically, for Gaussian data view, we add i.i.d. *N*(0, *σ*^2^) noise where *σ* = 0.5, 1.0, 1.5; for count data view, we add i.i.d. Poisson noise; for binary data view, we randomly shuffle a small proportion of the entries. We compute the adjusted Rand index between the baseline clustering and the one obtained on the perturbed data. We adopt the same approach for other existing methods. Table 16 shows the average adjusted Rand index of 10 replicates. For all values of *σ*, we see that iGecco+ tends to produce the most stable results.

**Table 16:** Stability and reproducibility of adaptive iGecco+ on simulated data. Adaptive iGecco+ and other existing methods are applied to the simulated data to obtain baseline clusterings. We then perturb the data by adding i.i.d. noise. Specifically, for Gaussian data view, we add i.i.d. *N*(0, *σ*^2^) noise where *σ* = 0.5, 1.0, 1.5; for count data view, we add i.i.d. Poisson noise; for binary data view, we randomly shuffle a small proportion of the entries. We compute the adjusted Rand index (ARI) between the baseline clustering and the one obtained on the perturbed data.

*σ*	A iGecco+	Hclust: Euclidean	Hclust: Gower	iCluster+	BCC
0.5	1.00 (0.0e-0)	0.85 (5.1e-2)	0.90 (1.4e-2)	0.69 (2.6e-2)	0.92 (1.6e-2)
1.0	1.00 (0.0e-0)	0.69 (8.3e-2)	0.89 (1.8e-2)	0.71 (3.0e-2)	0.90 (1.7e-2)
1.5	0.95 (3.4e-2)	0.66 (2.8e-2)	0.86 (2.0e-2)	0.70 (2.9e-2)	0.90 (2.0e-2)

## Appendix L. Noisy Data Sources

In this appendix, we show the performance of iGecco+ when a purely noisy data view is observed.

Often in real data, not all the data views observed contain clustering information, i.e., one or more data views might be pure noise. Our iGecco+ is able to filter out noisy data views by adaptively shrinking all the (noise) features in these data sets towards the loss-specific centers. We include a simulation study to demonstrate the performance of iGecco+ in the presence of some purely noisy data sets. Similar to the base simulation, each simulated data set consists of *n* = 120 observations with 3 clusters. Each cluster has an equal number of observations. Only the first data view contains clustering signal with the first 30 features being informative while the rest features being noisy; the rest two data views are pure noise.

Table 17 shows that iGecco+ still performs well in the presence of noise data sources by adaptively shrinking noise features.

**Table 17:** Adjusted Rand index of different methods in the presence of noisy data sources

Method	Adjusted Rand Index

Hclust: [**X**_1_**X**_2_**X**_3_] - Euclidean	0.40 (4.9e-2)
Hclust: [**X**_1_**X**_2_**X**_3_] - Gower	0.33 (4.8e-2)
iCluster+	0.88 (5.8e-2)
Bayesian Consensus Clustering	0.00 (2.8e-4)
Adaptive iGecco+	**0.99** (5.6e-3)

## Appendix M. Computation Time

In this section, we provide some computation run time results of iGecco(+) with different sample sizes and dimensions. We include results for both run times per iteration of the ADMM algorithm in Table 18 as well as the full training time for tuning parameter selection in Table 19. All timing results are run on a Dell XPS 15 with a 2.4 GHz Intel i5 processor and 8 GB of 2666 MHz DDR4 memory.

For training, we use BIC based approach to select tuning parameters for both iGecco and iGecco+ as it works well in practice and saves computation. It takes more time to train iGecco+ than iGecco as iGecco+ needs to select two tuning parameters (the number of clusters and features). Yet, our BIC based approach selects optimal tuning parameters in a reasonable amount of time. Note, for sample size *n* = 120 and feature size *p*_1_ = 200, *p*_2_ = 100, *p*_3_ = 50, it takes iClusterPlus hours for tuning parameter selection (using tune.iClusterPlus function in R).

**Table 18:** Run time results per iteration of iGecco(+) with different sample sizes and dimensions; the first three experiments use iGecco while the rest use iGecco+

	Sample Size *n*	*p*_1_	*p*_2_	*p*_3_	Computation Time (in seconds)
iGecco	120	10	10	10	0.0018
	300	10	10	10	0.0044
	120	30	30	30	0.0038

iGecco+	120	200	100	50	0.0041
	300	200	100	50	0.0105
	120	300	200	100	0.0055
	120	400	200	100	0.0061

**Table 19:** Training run time results of iGecco(+) with different sample sizes and dimensions; the first three experiments use iGecco with BIC to choose number of clusters while the rest use adaptive iGecco+ with BIC to choose number of clusters and features.

	Sample Size *n*	*p*_1_	*p*_2_	*p*_3_	Computation Time (in seconds)
iGecco	120	10	10	10	0.89
	300	10	10	10	2.58
	120	30	30	30	2.55

iGecco+	120	200	100	50	63.72
	300	200	100	50	117.94
	120	300	200	100	90.77
	120	400	200	100	104.04
